# Overcoming the Usual Reactivity of β-Nitroenones: Synthesis of Polyfunctionalized Homoallylic Alcohols and Conjugated Nitrotriene Systems

**DOI:** 10.1021/acs.joc.2c02669

**Published:** 2023-03-16

**Authors:** Lixia Yuan, Liudmila Kachalova, Muhammad E. I. Khan, Roberto Ballini, Marino Petrini, Alessandro Palmieri

**Affiliations:** ‡ Green Chemistry Group, School of Sciences and Technology, Chemistry Division, ChIP Research Center, University of Camerino, Via Madonna delle Carceri, Camerino, Macerata 62032, Italy; † Institute of BioPharmaceutical Research, Liaocheng University, Liaocheng 252059, People’s Republic of China

## Abstract

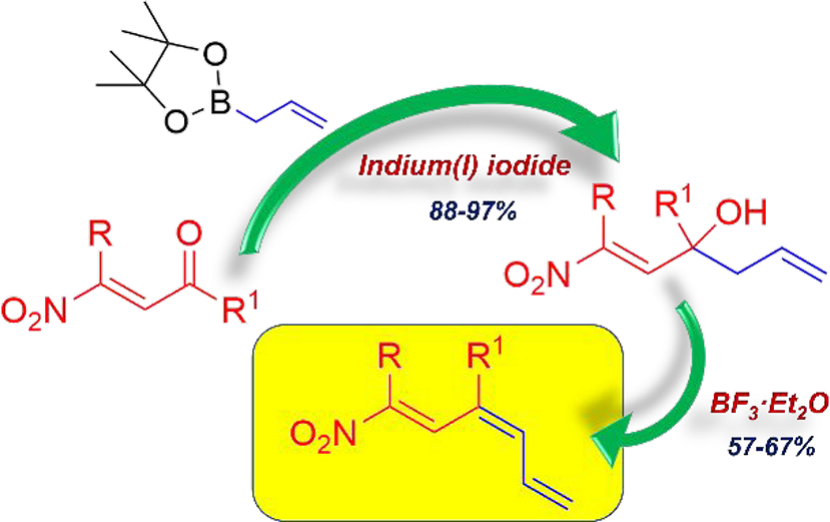

Herein, we report a new application of β-nitroenones as valuable building blocks for the preparation of polyfunctionalized homoallylic alcohols; they can be used as key precursors of conjugated nitrotriene systems. The synthesis of homoallylic alcohols was performed exploiting the chemoselective addition of metal allylating agents to the ketone moiety vs the nitroalkenyl group. The conversion of alcohols into nitrotrienes was achieved under Lewis-acid-promoted conditions. Both classes of compounds were obtained in good to excellent yields.

β-Nitroenones **1** represent a valuable class of nitroolefins characterized by the presence of nitro and ketone functionalities in the α and β positions to a double bond. The juxtaposition of these functionalities makes **1** highly reactive species toward a plethora of nucleophiles, and in particular, they chemoselectively react at position 2, behaving as excellent Michael acceptors (Figure 1). Over the years, the synthetic importance of these molecules was demonstrated by their usage for synthesizing heterocyclic systems such as pyrroles, furans, indoles,^1^ and carbonyl derivatives.^2^

**Figure 1:**
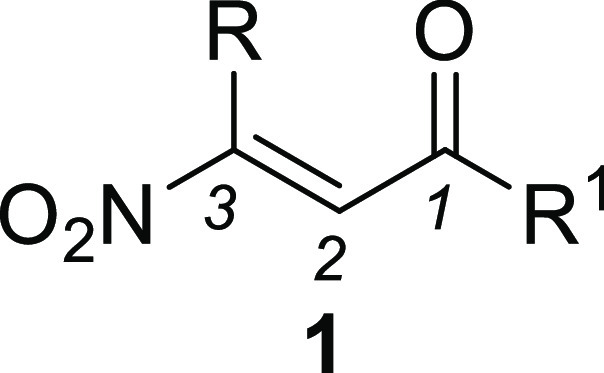
General structure of β-nitroenones **1**.

Following our ongoing research concerning the chemistry of β-nitroenones **1**, we have now discovered a peculiar reactivity of **1** when treated with metal allylating agents **2**. In fact, unlike the reactivity commonly observed with other nucleophilic species, compounds **2** exclusively react with the carbonyl group rather than the nitroalkene moiety, thus generating the corresponding homoallylic alcohols **3** (Scheme 1).

**Scheme 1:**
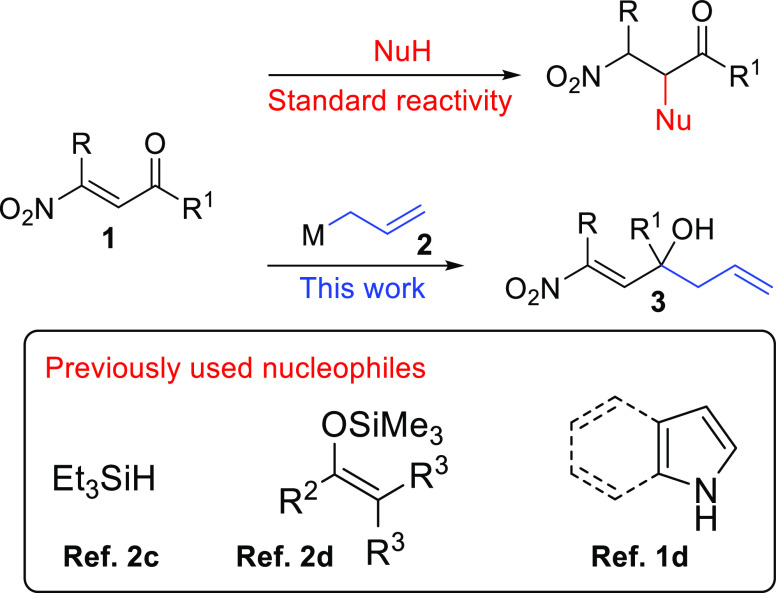
Reactivity of β-Nitroenones **1**

Homoallylic alcohols play a pivotal role as building blocks for the preparation of highly functionalized materials,^3^ as well as key precursors for common frameworks of many natural products and bioactive compounds.^4^ In this regard, due to the peculiar structure of **3**, in which the homoallylic alcohol portion is connected to the nitroalkene system, we explored and successfully achieved the dehydration of **3** into the conjugated nitrotriene systems **4** (Scheme 2).

**Scheme 2:**
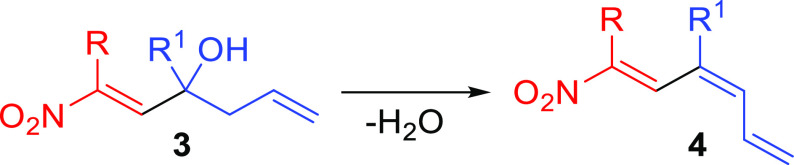
Synthesis of Nitro-Functionalized Triene Systems **4**

Although the chemistry of conjugated nitrodienes **5** has been largely studied (Figure 2),^5^ very little information concerning their homologues **4** is available in the literature. This is probably due to the difficulty of preparing these compounds, whose synthesis often requires elaborate starting materials and/or severe reaction conditions.^6^ In this context, our approach can be considered the first general and straightforward method to access conjugated nitrotrienes **4**.

**Figure 2:**
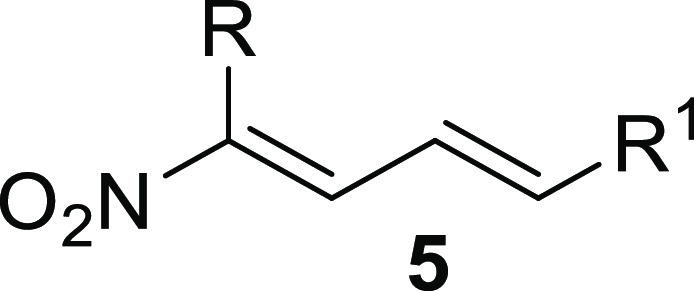
General structure of conjugated nitrodienes **5**.

First, we focused our attention on optimizing the allylation reaction. For this purpose, the conversion of **1a** into **3a** was selected as representative reaction, and a variety of allylating systems and reaction conditions were screened (Scheme 3).

**Scheme 3:**
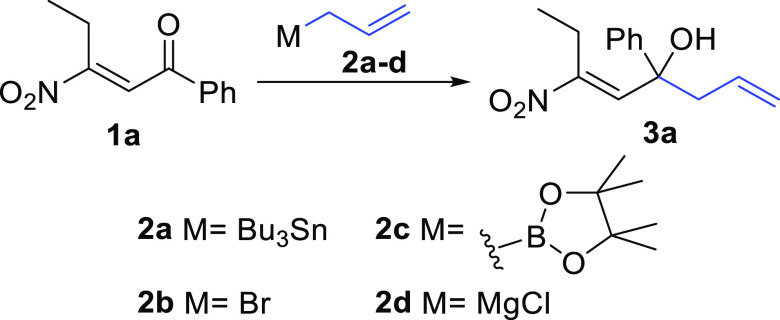
Test Reaction: Conversion of **1a** into **3a**

Based on the article of Kalita and Phukan relating to the allylation of chalcones,^7^ we first attempted the conversion of **1a** into **3a** using allyltributylstannane **2a** in the presence of CuI (0.25 equiv) and DMF. The reaction gave a modest result, and **3a** was isolated in 56% yield. No improvement was observed by changing the solvent (Table 1, entries a–c). Successively, the growing use in the literature of indium as an efficient metal for promoting the Barbier reaction^8^ spurred us to investigate the synthesis of **3a** starting from allyl bromide **2b** and **1a**. Once again, the product **3a** was isolated in a moderate yield of 58% and only in the presence of a significant excess of **2b** and indium (Table 1, entries d–g). Then, inspired by the Schneider and Kobayashi’s paper,^9^ we examined the allylation reaction of **1a** using the pinacolyl allylboronate **2c** in the presence of a catalytic amount of indium(I) iodide. To our delight, the reaction provided **3a** in 94% yield (Table 1, entry h). Extra catalytic systems based on indium species such as In/InB_3_ and In/InCl_3_ were also screened. These systems are supposed to generate in situ the corresponding In(I) halides, which however were less effective than InI in terms of both yield and reaction time due to the lower thermodynamic stability, according to what was previously reported.^9^ Finally, the reaction was attempted using allylmagnesium chloride **2d** without any appreciable result (Table 1, entry k).

**Table 1:** Optimization Studies of the Allylation Reaction

entry	compound **2**	reaction conditions	yield^a^ of **3a**
a	**2a** (1.2 equiv)	K_2_CO_3_ (1.2 equiv), CuI (0.25 equiv), DMF (0.7 M), 70 °C, 20 h	56%
b	**2a** (1.2 equiv)	K_2_CO_3_ (1.2 equiv), CuI (0.25 equiv), MeCN (0.7 M), 70 °C, 20 h	31%
c	**2a** (1.2 equiv)	K_2_CO_3_ (1.2 equiv), CuI (0.25 equiv), γ-valerolactone (0.7 M), 70 °C, 20 h	50%
d	**2b** (1.3 equiv)	In (1.5 equiv), EtOH (0.2 M), 0 °C, 7 h	21%
e	**2b** (2 equiv)	In (2 equiv), EtOH (0.2 M), 0 °C, 6 h	43%
f	**2b** (2 equiv)	In (2 equiv), EtOH (0.2 M), rt, 3 h	49%
g	**2b** (3 equiv)	In (2 equiv), EtOH (0.2 M), rt, 3 h	58%
h	**2c** (1.5 equiv)	InI (0.05 equiv), THF (0.2 M), 40 °C, 7 h	94%
i	**2c** (1.5 equiv)	In (0.20 equiv), InBr_3_ (0.1 equiv), THF (0.2 M), 40 °C, 30 h	85%
j	**2c** (1.5 equiv)	In (0.20 equiv), InCl_3_ (0.1 equiv), THF (0.2 M), 40 °C, 34 h	71%
k	**2d** (1.2 equiv)	THF (0.2 M), 0 °C, 20 h	

a Yield of the pure isolated product **3a**.

Next, we explored both the substrate generality for the allylation of β-nitroenones **1a**–**o** with **2c** catalyzed by 5 mol % of indium(I) iodide and the scalability of our protocol exploring the conversion of **1a** into **2a** on 5 mmol scale. Pleasingly, both studies provided excellent results. In fact, it was seen that the reaction demonstrated good generality, affording the target homoallylic alcohols **3a**–**o** in very good yields (Figure 3), and the large-scale reaction provided **3a** in 95% yield (see SI).

**Figure 3:**
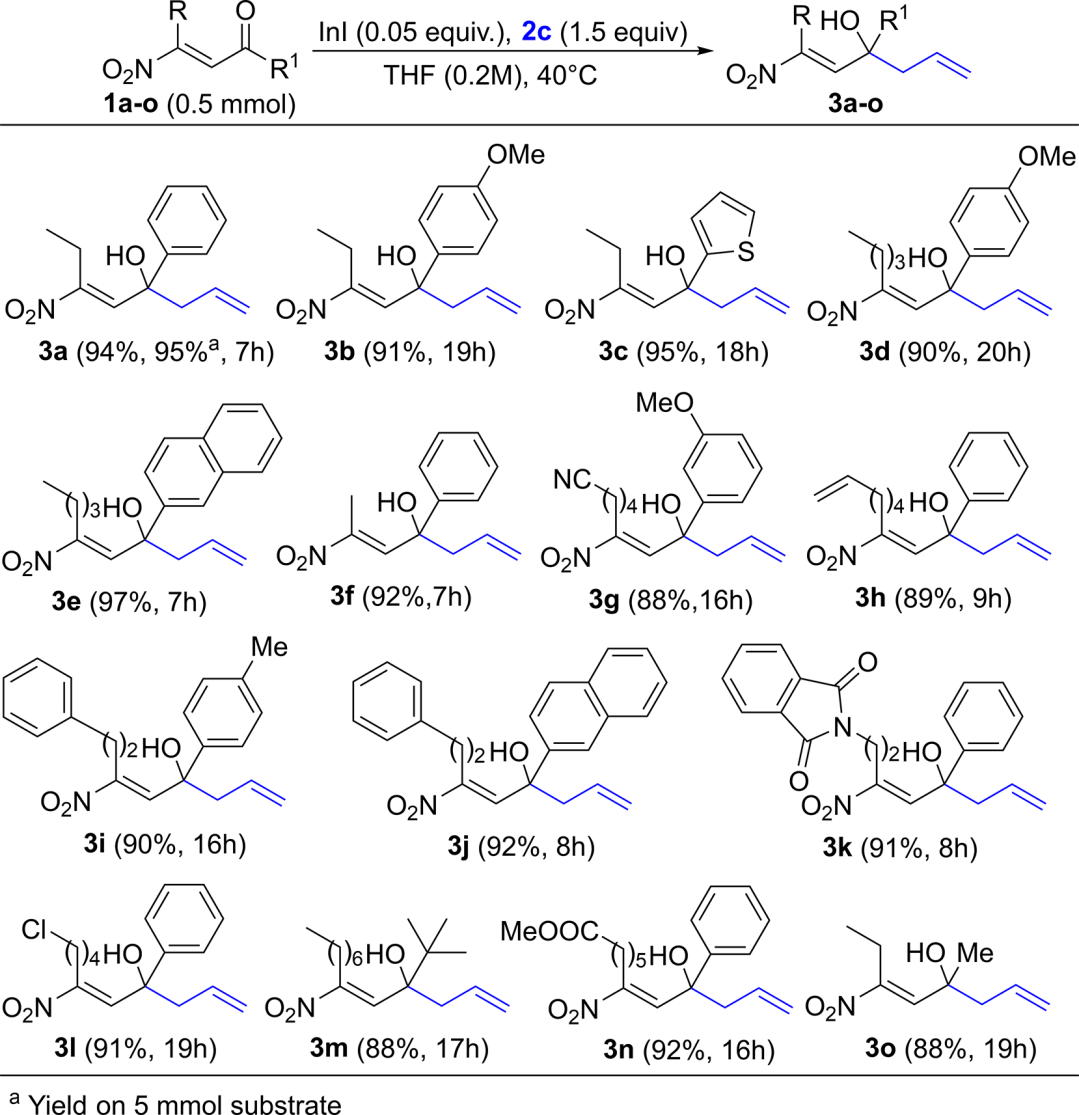
Substrate scope demonstration: synthesis of homoallylic alcohols **3**.

Later, we investigated the use of nitroalkenyl alcohols **3** as valuable building blocks for the possible synthesis of conjugated nitrotriene systems **4**. This hitherto unknown class of nitro derivatives may open new scenarios in the synthesis of functionalized molecules exploiting the chemistry of trienes.^10^ In order to find the best reaction conditions and following the available literature,^11^ we initially explored the conversion of **3a** into **4a** using *p*-toluenesulfonic acid in toluene under reflux conditions, which however provided a complex mixture of inseparable compounds. Conversely, at room temperature, the reaction was completely ineffective. Then, we tested the dehydration reaction utilizing boron trifluoride diethyl etherate in dichloromethane (Table 2).^12^ Under these conditions, at room temperature, we were able to isolate 43% of regioisomeric nitrotrienes **4a** and **5a** in a 75:25 ratio. After a careful screening of the reaction parameters, we obtained the best yield (67%) by working at −10 °C and in the presence of 1.5 equiv of BF_3_·Et_2_O. Noteworthy, the formation of the other regioisomer **5a** was limited to less than 10%. The increase of the observed regioselectivity at lower temperature is probably the result of better kinetic control in the formation of compounds **4**. Moreover, we attempted to convert **4a** into **5a** with the aim to obtain a single regioisomer. With this scope, the reaction was stirred at room temperature for 24 h; however, we observed extensive degradation of the product without any change concerning the **4a:5a** regioisomeric ratio (Table 2, entry f).

**Table 2:**
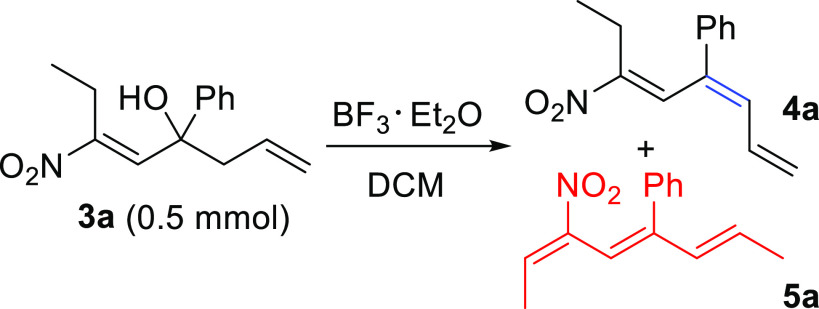
Optimization Studies of the Dehydration Reaction Using BF_3_·Et_2_O

entry	BF_3_·Et_2_O (equiv )	reaction conditions	yield^a^ of **4a** + **5a** (**4a**:**5a**)
a	1.2	rt, 2.5 h	43% (75:25)
b	1.2	0 °C, 2.5 h	49% (80:20)
c	1.5	0 °C, 2.5 h	54% (80:20)
d	1.5	–10 °C, 5 h	67% (90:10)
e	1.5	–20 °C, 24 h	traces
f	1.5	rt, 24 h	17% (75:25)
g	1.5	–10 °C, 5 h	69% (90:10)^b^

a Yield of sum of products **4a** and **5a**.

b Reaction performed on 2 mmol scale.

The reaction was also investigated using a different solvent such as 1,2-dichloroethane (DCE) and additional Lewis acids including AlCl_3_, EtAlCl_2_, BBr_3_, and BF_3_ and AlCl_3_ supported on silica. The use of DCE instead of DCM as well as the use of aluminum trichloride led to a notable drop of the efficiency, giving **4a** in 32% (5 h, −10 °C) and 22% (8 h, rt) yield, respectively. The reaction promoted by BBr_3_ at −10 °C for 6 h led to **4a** in just 41% conversion, while warming to room temperature resulted in complete degradation of the expected product. Finally, use of ethylaluminum dichloride and the heterogeneous BF_3_ and AlCl_3_ was completely ineffective.

We also repeated the reaction starting from 2 mmol of **3a** to demonstrate the larger scale viability of the protocol. Even under these conditions the process was very efficient; in fact, the regioisomeric mixture of **4a** and **5a** (90:10) was isolated in 69% yield (see SI), a value comparable to that obtained on a smaller scale (Table 2, entry g).

With the aim to establish the configuration of the newly generated double bond, we performed NOESY experiments on the isomer **4a**. These studies highlighted a strong cross-peak signal between the proton at C-2 and that at C-5, thus confirming the 3*E* geometry for the prevailing regioisomer **4a** (Figure 4).

**Figure 4:**
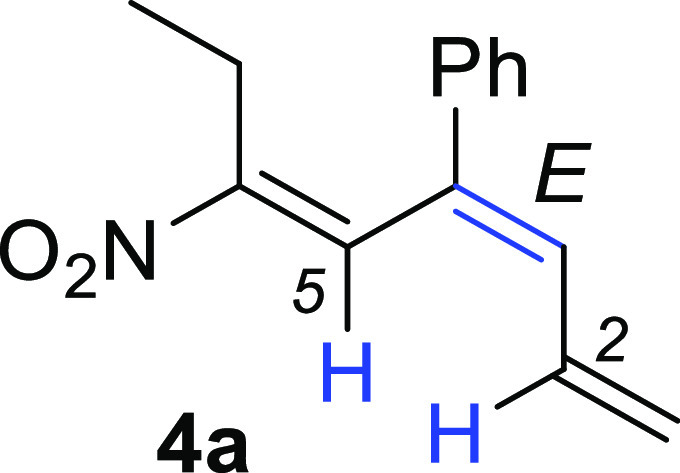
NOESY experiment concerning the isomer **4a**.

The formation of the *E* diastereomer can be explained considering the initial generation of the carbocation **A**, which potentially can assume conformation **B** or **C** to give the *Z* or *E* configuration, respectively. In particular, the higher stability of **C**, in which the steric hindrance and electronic repulsion between the alkyl and the phenyl groups is minimized, over the **B** conformer leads exclusively to the *E* double-bond geometry (Scheme 4).

**Scheme 4:**
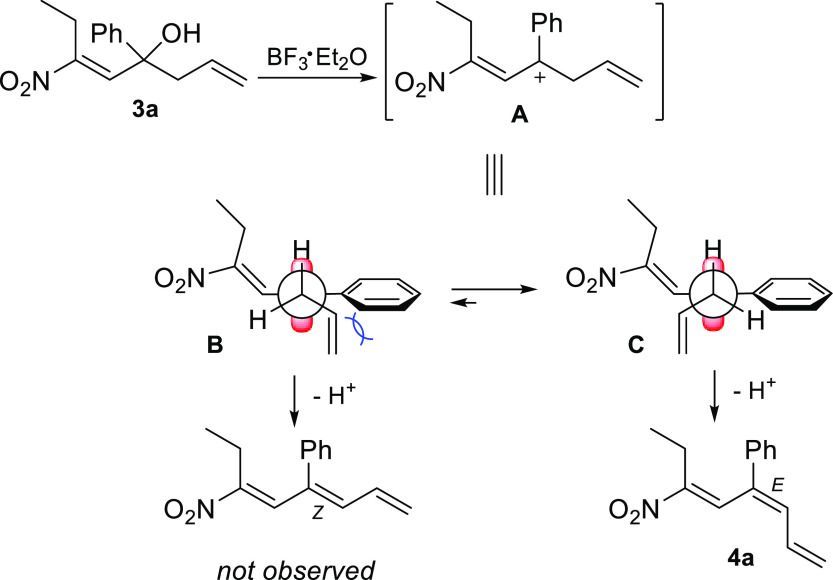
Possible Explanation for the Formation of the *E* Diastereomer **4a**

Finally, we applied the optimized reaction conditions to a variety homoallylic alcohols **3**. In all cases, products **4** were obtained in satisfactory yields, albeit around 10% of the **5** isomer was always detected except for compound **4i** which was obtained as a single regioisomer (Figure 5). The dehydration was also attempted on alkyl derivative **3m**, bearing a 4-*tert*-butyl group, which however was unreactive under the reaction conditions, presumably because of the reduced stability of the carbocationic intermediate.

**Figure 5:**
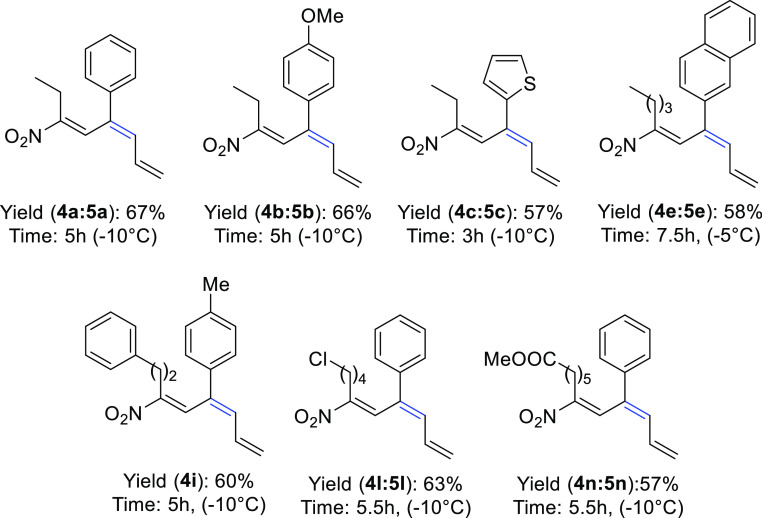
Synthesis of conjugated nitrotriene systems **4**.

In conclusion, we disclosed a new significant reactivity of β-nitroenones in combination with allylating agents which enables one to prepare a series of polyfunctionalized homoallylic alcohols in excellent yields, demonstrating once again the utility of β-nitroenones as pivotal starting materials in organic synthesis. The usefulness of the obtained allylated derivatives has been demonstrated by their stereoselective conversion into functionalized nitrotrienes under mild conditions.

## Experimental Section

### General Remarks

^1^H NMR analyses were recorded at 400 MHz on a Varian Mercury Plus 400. ^13^C NMR analyses were recorded at 100 MHz. IR spectra were recorded with a PerkinElmer FTIR spectrometer Spectrum Two UATR. Microanalyses were performed with a CHNS-O analyzer model EA 1108 from Fisons Instruments. GS-MS analyses were obtained on a Hewlett-Packard GC/MS 6890N that works with the EI technique (70 eV). Compounds **1a**–**o** were prepared starting from alkyl- and arylglyoxals and nitro compounds by following reported procedures;^14^**1a** was a known compound,^2b^ while compounds **1b**–**o** were new compounds. Allyltributyltin **2a** was purchased from Sigma-Aldrich (code 271411) and used as received. Allyl bromide **2b** was purchased from Sigma-Aldrich (code A29585) and used as received. Allylboronic acid pinacol ester **2c** was purchased from Sigma-Aldrich (code 324647) and used as received. Allylmagnesium chloride **2d** was purchased from Sigma-Aldrich (code 225908) and used as received. Boron trifluoride diethyl etherate was purchased from Sigma-Aldrich (code 216607) and used as received. Tetrahydrofuran was purchased from Sigma-Aldrich (code 87368) and distilled over sodium before usage. Dichloromethane (stabilized with amylene) was purchased from Carlo Erba (code 463314) and used as received. Indium(I) iodide was prepared according to the literature.^13^ Heating for synthesizing compounds **3a**–**o** was accomplished by means of a heating magnetic stirrer equipped with an aluminum heating block. Configuration assignment of compound **4a** was made with additional information from the NOESY experiment.

### General Procedure for the Preparation of Compounds **3**

An oven-dried round-bottom flask with a magnetic stir bar, maintained under inert atmosphere, was charged with the appropriate β-nitroenones **1** (0.5 mmol), dry THF (2.5 mL), InI (0.025 mmol, 6 mg), and the allylboronic acid pinacol ester **2c** (0.75 mmol, 141 μL). The resulting mixture was vigorously stirred at 40 °C for the appropriate time (see Figure 3), diluted with dichloromethane (20 mL), and treated with a saturated aqueous solution of NaHCO_3_ (10 mL). After phase separation, the aqueous phase was extracted with dichloromethane (2 × 20 mL), and the combined organic layers were dried with dry Na_2_SO_4_. Finally, the solution was filtered and concentrated in vacuo to give the crude products **3**, which were purified by flash column chromatography (hexane/ethyl acetate).

#### (*E*)-6-Nitro-4-phenylocta-1,5-dien-4-ol **3a**

Flash chromatography on silica gel using hexane/EtOAc = 95:5 as eluent yielded **3a** (116 mg, 94% yield) as a pale yellow oil. IR (cm^–1^, neat): 703, 924, 1334, 1520, 1634, 3392. ^1^H NMR (400 MHz, CDCl_3_) δ: 7.47–7.42 (m, 2H), 7.40–7.34 (m, 3H), 7.31–7.25 (m, 1H), 5.73–5.60 (m, 1H), 5.32–5.24 (m, 2H), 2.78 (d, 2H, *J* = 7.7 Hz), 2.76 (q, 2H, *J* = 7.4 Hz), 2.50 (br s, 1H), 0.92 (t, 3H, *J* = 7.3 Hz). ^13^C{^1^H} NMR (100 MHz, CDCl_3_) δ: 155.8, 144.3, 138.3, 131.7, 128.9, 127.9, 125.3, 122.3, 74.5, 49.1, 20.8, 12.5. GC-MS (70 eV): *m*/*z* 206 (59), 159 (100), 131 (12), 105 (37), 77 (32). Anal. Calcd for C_14_H_17_NO_3_ (247.29): C, 68.00; H, 6.93; N, 5.66. Found: C, 68.05; H, 6.96; N, 5.69.

#### (*E*)-4-(4-Methoxyphenyl)-6-nitroocta-1,5-dien-4-ol **3b**

Flash chromatography on silica gel using hexane/EtOAc = 95:5 as eluent yielded **3b** (126 mg, 91% yield) as a pale yellow oil. IR (cm^–1^, neat): 832, 926, 1034, 1176, 1248, 1334, 1513, 1608, 3464. ^1^H NMR (400 MHz, CDCl_3_) δ: 7.38–7.32 (m, 3H), 6.89 (d, 2H, *J* = 8.9 Hz), 5.74–5.60 (m, 1H), 5.29 (s, 1H), 5.27–5.23 (m, 1H), 3.81 (s, 3H), 2.81–2.71 (m, 4H), 2.38 (br s, 1H), 0.95 (t, 3H, *J* = 7.3 Hz). ^13^C{^1^H} NMR (100 MHz, CDCl_3_) δ: 159.2, 155.4, 138.3, 136.2, 131.9, 126.6, 122.1, 114.2, 74.3, 55.6, 48.9, 20.7, 12.6. GC-MS (70 eV): *m*/*z* 236 (73), 189 (100), 135 (42), 81 (15), 77 (13). Anal. Calcd for C_15_H_19_NO_4_ (277.32): C, 64.97; H, 6.91; N, 5.05. Found: C, 65.02; H, 6.94; N, 5.02.

#### (*E*)-6-Nitro-4-(thiophen-2-yl)octa-1,5-dien-4-ol **3c**

Flash chromatography on silica gel using hexane/EtOAc = 95:5 as eluent yielded **3c** (120 mg, 95% yield) as a pale yellow oil. IR (cm^–1^, neat): 709, 931, 1331, 1434, 1525, 1609, 3528. ^1^H NMR (400 MHz, CDCl_3_) δ: 7.27 (dd, 1H, *J* = 4.4, 1.9 Hz), 7.24 (s, 1H), 7.00–6.96 (m, 2H), 5.82–5.68 (m, 1H), 5.33–5.31 (m, 1H), 5.30–5.26 (m, 1H), 2.92–2.76 (m, 4H), 2.65 (br s, 1H), 1.04 (t, 3H, *J* = 7.3 Hz). ^13^C{^1^H} NMR (100 MHz, CDCl_3_) δ: 155.3, 148.7, 136.8, 131.4, 127.4, 125.6, 124.0, 122.5, 73.9, 49.2, 20.7, 12.9. GC-MS (70 eV): *m*/*z* 212 (61), 165 (100), 111 (47), 81 (19), 39 (17). Anal. Calcd for C_12_H_15_NO_3_S (253.32): C, 56.90; H, 5.97; N, 5.53; S, 12.66. Found: C, 56.93; H, 6.00; N, 5.56; S, 12.70.

#### (*E*)-4-(4-Methoxyphenyl)-6-nitrodeca-1,5-dien-4-ol **3d**

Flash chromatography on silica gel using hexane/EtOAc = 95:5 as eluent yielded **3d** (137 mg, 90% yield) as a pale yellow oil. IR (cm^–1^, neat): 739, 833, 920, 1035, 1177, 1252, 1334, 1508, 1607, 3539. ^1^H NMR (400 MHz, CDCl_3_) δ: 7.35 (d, 2H, *J* = 8.9 Hz), 7.33 (s, 1H), 6.89 (d, 2H, *J* = 8.9 Hz), 5.73–5.61 (m, 1H), 5.29 (s, 1H), 5.27–5.23 (m, 1H), 3.81 (s, 3H), 2.78–2.70 (m, 4H), 2.37 (br s, 1H), 1.39–1.15 (m, 4H), 0.83 (t, 3H, *J* = 7.1 Hz). ^13^C{^1^H} NMR (100 MHz, CDCl_3_) δ: 159.2, 154.5, 138.4, 136.3, 131.9, 126.6, 122.1, 114.1, 74.3, 55.5, 48.9, 26.9, 30.2, 22.9, 13.9. GC-MS (70 eV): *m*/*z* 264 (67), 243 (12), 217 (100), 175 (13), 135 (91), 77 (21). Anal. Calcd for C_17_H_23_NO_4_ (305.37): C, 66.86; H, 7.59; N, 4.56. Found: C, 66.91; H, 7.63; N, 4.59.

#### (*E*)-4-(Naphthalen-2-yl)-6-nitrodeca-1,5-dien-4-ol **3e**

Flash chromatography on silica gel using hexane/EtOAc = 95:5 as eluent yielded **3e** (158 mg, 97% yield) as a pale yellow oil. IR (cm^–1^, neat): 748, 823, 1331, 1426, 1517, 1607, 1635, 3062, 3545. ^1^H NMR (400 MHz, CDCl_3_) δ: 7.94 (d, 1H, *J* = 1.8 Hz), 7.88–7.81 (m, 3H), 7.54–7.48 (m, 3H), 7.47 (s, 1H), 5.74–5.62 (m, 1H), 5.35–5.27 (m, 2H), 2.88 (d, 2H, *J* = 7.3 Hz), 2.74 (dd, 2H, *J* = 8.3, 7.0 Hz), 2.54 (br s, 1H), 1.08–1.37 (m, 4H), 0.74 (t, 3H, *J* = 7.2 Hz). ^13^C{^1^H} NMR (100 MHz, CDCl_3_) δ: 155.2, 141.5, 137.9, 133.3, 132.8, 131.7, 128.8, 128.4, 127.8, 126.8, 126.6, 124.1, 123.5, 122.4, 74.6, 49.0, 30.0, 27.0, 22.8, 13.8. GC-MS (70 eV): *m*/*z* 278 (15), 263 (64), 237 (17), 207 (17), 155 (100), 127 (76), 77 (12). Anal. Calcd for C_20_H_23_NO_3_ (325.41): C, 73.82; H, 7.12; N, 4.30. Found: C, 73.78; H, 7.09; N, 4.27.

#### (*E*)-6-Nitro-4-phenylhepta-1,5-dien-4-ol **3f**

Flash chromatography on silica gel using hexane/EtOAc = 95:5 as eluent yielded **3f** (107 mg, 92% yield). IR (cm^–1^, neat): 698, 724, 987, 1326, 1519, 1608, 1640, 3062, 3537. ^1^H NMR (400 MHz, CDCl_3_) δ: 7.47–7.45 (m, 2H), 7.44 (s, 1H), 7.40–7.35 (m, 2H), 7.32–7.27 (m, 1H), 5.74–5.61 (m, 1H), 5.33–5.24 (m, 2H), 2.79 (d, 2H, *J* = 7.4 Hz), 2.46 (br s, 1H), 2.24 (s, 3H). ^13^C{^1^H} NMR (100 MHz, CDCl_3_) δ: 150.3, 143.8, 138.3, 131.5, 128.7, 127.7, 125.1, 122.0, 74.2, 48.7, 13.6. GC-MS (70 eV): *m*/*z* 192 (60), 145 (100), 105 (29), 77 (31), 67 (32). Anal. Calcd for C_13_H_15_NO_3_ (233.27): C, 66.94; H, 6.48; N, 6.00. Found: C, 66.99; H, 6.52; N, 5.97.

#### (*E*)-8-Hydroxy-8-(3-methoxyphenyl)-6-nitroundeca-6,10-dienenitrile **3g**

Flash chromatography on silica gel using hexane/EtOAc = 95:5 as eluent yielded **3g** (145 mg, 88% yield) as a pale yellow oil. IR (cm^–1^, neat): 698, 1042, 1247, 1328, 1432, 1520, 1583, 1600, 1640, 2249, 3460. ^1^H NMR (400 MHz, CDCl_3_) δ: 7.40 (s, 1H), 7.30 (t, 1H, *J* = 7.9 Hz), 7.03–6.96 (m, 2H), 6.87–6.81 (m, 1H), 5.70–5.57 (m, 1H), 5.32 (d, 1H, *J* = 1.0 Hz), 5.30–5.26 (m, 1H), 3.82 (s, 3H), 2.86–2.69 (m, 4H), 2.52 (br s, 1H), 2.27 (t, 2H, *J* = 7.2 Hz),1.65–1.34 (m, 4H). ^13^C{^1^H} NMR (100 MHz, CDCl_3_) δ: 159.9, 152.9, 145.5, 138.9, 131.2, 129.9, 122.4, 119.5, 117.4, 112.5, 111.4, 74.2, 55.3, 48.7, 26.9, 26.0, 25.0, 16.7. GC-MS (70 eV): *m*/*z* 284 (25), 268 (93), 229 (30), 187 (16), 135 (100), 107 (27), 77 (34). Anal. Calcd for C_18_H_22_N_2_O_4_ (330.38): C, 65.44; H, 6.71; N, 8.48. Found: C, 65.49; H, 6.74; N, 8.51.

#### (*E*)-6-Nitro-4-phenyldodeca-1,5,11-trien-4-ol **3h**

Flash chromatography on silica gel using hexane/EtOAc = 95:5 as eluent yielded **3h** (134 mg, 89% yield) as a pale yellow oil. IR (cm^–1^, neat): 699, 912, 994, 1331, 1521, 1640, 3540. ^1^H NMR (400 MHz, CDCl_3_) δ: 7.47–7.42 (m, 2H), 7.40–7.34 (m, 3H), 7.32–7.26 (m, 1H), 5.80–5.59 (m, 2H), 5.30 (d, 1H, *J* = 0.8 Hz), 5.28–5.24 (m, 1H), 5.00–4.88 (m, 2H), 2.78 (d, 2H, *J* = 7.3 Hz), 2.76–2.70 (m, 2H), 2.41 (br s, 1H), 1.96 (q, 2H, *J* = 6.9 Hz), 1.14–1.12 (m, 4H). ^13^C{^1^H} NMR (100 MHz, CDCl_3_) δ: 154.4, 144.0, 138.5, 138.1, 131.5, 128.6, 127.7, 125.1, 122.1, 114.5, 74.2, 48.8, 33.3, 28.6, 27.2, 26.8. GC-MS (70 eV): *m*/*z* 260 (7), 213 (11), 186 (18), 105 (100), 91 (10), 77 (26), 41 (14). Anal. Calcd for C_18_H_23_NO_3_ (301.39): C, 71.73; H, 7.69; N, 4.65. Found: C, 71.77; H, 7.73; N, 4.62.

#### (*E*)-6-Nitro-8-phenyl-4-(*p*-tolyl)octa-1,5-dien-4-ol **3i**

Flash chromatography on silica gel using hexane/EtOAc = 95:5 as eluent yielded **3i** (152 mg, 90% yield) as a pale yellow oil. IR (cm^–1^, neat): 699, 733, 1326, 1453, 1521, 1603, 1640, 3540. ^1^H NMR (400 MHz, CDCl_3_) δ: 7.46 (s, 1H), 7.34–7.29 (m, 4H), 7.26–7.18 (m, 3H), 7.17–7.12 (m, 2H), 5.65–5.54 (m, 1H), 5.27–5.20 (m, 2H), 3.20–3.06 (m, 2H), 2.79–2.60 (m, 4H), 2.38 (s, 3H), 2.05 (br s, 1H). ^13^C{^1^H} NMR (100 MHz, CDCl_3_) δ: 152.5, 140.8, 140.7, 139.5, 137.5, 131.5, 129.4, 128.7, 128.5, 126.3, 125.0, 121.6, 74.4, 48.5, 33.9, 29.1, 21.0. GC-MS (70 eV): *m*/*z* 290 (19), 275 (48), 248 (9), 199 (12), 119 (100), 91 (82), 65 (24). Anal. Calcd for C_21_H_23_NO_3_ (337.42): C, 74.75; H, 6.87; N, 4.15. Found: C, 74.79; H, 6.90; N, 4.12.

#### (*E*)-4-(Naphthalen-2-yl)-6-nitro-8-phenylocta-1,5-dien-4-ol **3j**

Flash chromatography on silica gel using hexane/EtOAc = 95:5 as eluent yielded **3j** (172 mg, 92% yield) as a pale yellow oil. IR (cm^–1^, neat): 476, 734, 748, 1326, 1521, 1600, 1639, 3027, 3537. ^1^H NMR (400 MHz, CDCl_3_) δ: 7.94–7.85 (m, 4H), 7.59 (s, 1H), 7.58–7.52 (m, 2H), 7.50 (dd, 1H, *J* = 8.7, 1.9 Hz), 7.29–7.17 (m, 3H), 7.06 (d, 2H, *J* = 7.0 Hz), 5.67–5.56 (m, 1H), 5.33–5.22 (m, 2H), 3.21–3.07 (m, 2H), 2.87–2.69 (m, 3H), 2.65–2.55 (m, 1H), 2.20 (br s, 1H). ^13^C{^1^H} NMR (100 MHz, CDCl_3_) δ: 152.9, 141.0, 140.6, 139.2, 133.1, 132.6, 131.3, 128.7, 128.6, 128.5, 128.2, 127.6, 126.6, 126.4, 126.3, 123.9, 123.2, 122.0, 74.5, 48.5, 33.8, 29.1. GC-MS (70 eV): *m*/*z* 326 (34), 311 (34), 284 (59), 207 (29), 155 (100), 127 (90), 91 (34), 77 (17). Anal. Calcd for C_24_H_23_NO_3_ (373.45): C, 77.19; H, 6.21; N, 3.75. Found: C, 77.24; H, 6.18; N, 3.78.

#### (*E*)-2-(5-Hydroxy-3-nitro-5-phenylocta-3,7-dien-1-yl)isoindoline-1,3-dione **3k**

Flash chromatography on silica gel using hexane/EtOAc = 95:5 as eluent yielded **3k** (179 mg, 91% yield) as a pale yellow solid. Mp: 134–176 °C. IR (cm^–1^, neat): 701, 716, 935, 1331, 1398, 1517, 1703, 1770, 3073, 3513. ^1^H NMR (400 MHz, CDCl_3_) δ: 7.80–7.77 (m, 2H), 7.72–7.68 (m, 2H), 7.50 (s, 1H), 7.28–7.11 (m, 5H), 5.49–5.37 (m, 1H), 5.08 (dd, 1H, *J* = 10.2, 1.8 Hz), 4.94 (dd, 1H, *J* = 17.1, 1.6 Hz), 3.99–3.84 (m, 2H), 3.28–3.22 (m, 2H), 2.74 (dd, 1H, *J* = 13.7, 6.5 Hz), 2.67 (br s, 1H), 2.51 (dd, 1H, *J* = 13.8, 8.2 Hz). ^13^C{^1^H} NMR (100 MHz, CDCl_3_) δ: 168.5, 149.4, 143.4, 141.2, 134.1, 132.4, 131.3, 128.9, 127.9, 125.0, 123.5, 122.2, 74.8, 48.5, 36.0, 26.5. GC-MS (70 eV): *m*/*z* 345 (35), 330 (38), 183 (99), 171 (47), 160 (65), 105 (100), 77 (80). Anal. Calcd for C_22_H_20_N_2_O_5_ (392.41): C, 67.34; H, 5.14; N, 7.14. Found: C, 67.38; H, 5.17; N, 7.11.

#### (*E*)-10-Chloro-6-nitro-4-phenyldeca-1,5-dien-4-ol **3l**

Flash chromatography on silica gel using hexane/EtOAc = 95:5 as eluent yielded **3l** (141 mg, 91% yield) as a yellow oil. IR (cm^–1^, neat): 699, 1331, 1520, 3537. ^1^H NMR (400 MHz, CDCl_3_) δ: 7.47–7.35 (m, 5H), 7.32–7.27 (m, 1H), 5.72–5.59 (m, 1H), 5.33–5.25 (m, 2H), 3.44 (dt, 2H, *J* = 6.8, 0.8 Hz), 2.81–2.74 (m, 4H), 2.47 (br s, 1H), 1.73–1.63 (m, 2H), 1.60–1.46 (m, 1H), 1.44–1.31 (m, 1H). ^13^C{^1^H} NMR (100 MHz, CDCl_3_) δ: 153.5, 143.9, 138.7, 131.3, 128.7, 127.8, 125.0, 122.3, 74.3, 48.7, 44.4, 32.2, 26.1, 25.2. GC-MS (70 eV): *m*/*z* 268 (43), 221 (81), 105 (100), 77 (54), 41 (17). Anal. Calcd for C_16_H_20_ClNO_3_ (309.79): C, 62.03; H, 6.51; N, 4.52. Found: C, 62.08; H, 6.48; N, 4.55.

#### (*E*)-4-(*tert*-Butyl)-6-nitrotrideca-1,5-dien-4-ol **3m**

Flash chromatography on silica gel using hexane/EtOAc = 95:5 as eluent yielded **3m** (131 mg, 88% yield) as a yellow oil. IR (cm^–1^, neat): 1329, 1522, 3558. ^1^H NMR (400 MHz, CDCl_3_) δ: 7.10 (s, 1H), 5.80–5.69 (m, 1H), 5.27 (d, 1H, *J* = 10.1 Hz), 5.23 (d, 1H, *J* = 17.0 Hz), 3.06–2.98 (m, 1H), 2.84–2.76 (m, 1H), 2.61 (dd, 1H, *J* = 13.5, 5.5 Hz), 2.34 (dd, 1H, *J* = 13.6, 9.4 Hz), 1.80 (br s, 1H), 1.56–1.47 (m, 1H), 1.43–1.21 (m, 9H), 1.04 (s, 9H), 0.90 (t, 3H, *J* = 7.0 Hz). ^13^C{^1^H} NMR (100 MHz, CDCl_3_) δ: 154.6, 136.7, 133.2, 121.0, 78.8, 41.3, 39.2, 31.7, 29.6, 28.9, 28.6, 26.4, 25.4, 22.6, 14.0. GC-MS (70 eV): *m*/*z* 256 (24), 209 (30), 139 (15), 109 (14), 95 (18), 81 (25), 69 (100), 57 (90), 41 (74). Anal. Calcd for C_17_H_31_NO_3_ (297.44): C, 68.65; H, 10.51; N, 4.71. Found: C, 68.60; H, 10.54; N, 4.74.

#### Methyl (*E*)-9-Hydroxy-7-nitro-9-phenyldodeca-7,11-dienoate **3n**

Flash chromatography on silica gel using hexane/EtOAc = 95:5 as eluent yielded **3n** (160 mg, 92% yield) as a pale yellow oil. IR (cm^–1^, neat): 699, 1331, 1520, 1732, 3484. ^1^H NMR (400 MHz, CDCl_3_) δ: 7.46–7.24 (m, 6H), 5.72–5.58 (m, 1H), 5.31–5.23 (m, 2H), 3.65 (s, 3H), 2.78 (d, 2H, *J* = 7.3 Hz), 2.73 (dd, 2H, *J* = 8.5, 6.0 Hz), 2.57 (br s, 1H), 2.23 (t, 2H, *J* = 7.5 Hz), 1.59–1.47 (m, 2H), 1.43–1.11 (m, 4H). ^13^C{^1^H} NMR (100 MHz, CDCl_3_) δ: 174.2, 154.2, 144.0, 138.3, 131.5, 128.6, 127.7, 125.1, 122.0, 74.3, 51.5, 48.8, 33.8, 28.7, 27.2, 26.6, 24.3. GC-MS (70 eV): *m*/*z* 300 (6), 285 (13), 199 (22), 197 (18), 105 (100), 77 (41). Anal. Calcd for C_19_H_25_NO_5_ (347.41): C, 65.69; H, 7.25; N, 4.03. Found: C, 65.73; H, 7.28; N, 4.07.

#### (*E*)-4-Methyl-6-nitroocta-1,5-dien-4-ol **3o**

Flash chromatography on silica gel using hexane/EtOAc = 90:10 as eluent yielded **3o** (81 mg, 88% yield) as a pale yellow oil. IR (cm^–1^, neat): 735, 921, 1334, 1457, 1519, 1641, 3533. ^1^H NMR (400 MHz, CDCl_3_) δ: 7.03 (s, 1H), 5.89–5.76 (m, 1H), 5.33–5.19 (m, 2H), 2.95 (dq, 2H, *J* = 7.3, 1.7 Hz), 2.52–2.46 (m, 1H), 2.42–2.35 (m, 1H), 1.94 (br s, 1H), 1.45 (s, 3H), 1.13 (t, 3H, *J* = 7.3 Hz). ^13^C{^1^H} NMR (100 MHz, CDCl_3_) δ: 154.6, 138.4, 132.0, 121.1, 71.7, 47.8, 28.4, 20.1, 13.2. GC-MS (70 eV): *m*/*z* 144 (45), 97 (100), 43 (58). Anal. Calcd for C_9_H_15_NO_3_ (185.22): C, 58.36; H, 8.16; N, 7.56. Found: C, 58.40; H, 8.19; N, 7.58.

### General Procedure for the Preparation of Compounds **4**

BF_3_·Et_2_O (0.75 mmol, 93 μL) was added dropwise at −10 °C to a stirred solution of the appropriate homoallylic alcohol **3** (0.5 mmol) in dichloromethane (5 mL). The reaction was stirred at the same temperature (−5 °C for compound **3e**) for the appropriate time (see Figure 4), diluted with dichloromethane (10 mL), and treated with a saturated aqueous solution of NaHCO_3_ (10 mL). After phase separation, the aqueous phase was extracted with dichloromethane (2 × 20 mL), and the combined organic layers were dried with dry Na_2_SO_4_. Finally, the solution was filtered and concentrated in vacuo to give the crude regioisomeric products **4** and **5**, which were purified by flash column chromatography (hexane/ethyl acetate).

#### ((3*E*,5*E*)-6-Nitroocta-1,3,5-trien-4-yl)benzene **4a** (Major Regioisomer)

Flash chromatography on silica gel using hexane/EtOAc = 98:2 as eluent yielded a 90:10 mixture of **4a** and **5a** (77 mg, 67% yield) as a pale yellow oil. Major regioisomer **4a**. IR (cm^–1^, neat): 695, 760, 1333, 1520, 1632. ^1^H NMR (400 MHz, CDCl_3_) δ: 7.81 (s, 1H), 7.44–7.29 (m, 5H), 6.73–6.48 (m, 2H), 5.51 (d, 1H, *J* = 16.4 Hz), 5.39 (d, 1H, *J* = 9.9 Hz), 2.42 (q, 2H, *J* = 7.4 Hz), 0.96 (t, 3H, *J* = 7.4 Hz). ^13^C{^1^H} NMR (100 MHz, CDCl_3_) δ: 155.7, 138.8, 133.8, 133.1, 132.7, 130.1, 128.5, 128.4, 126.7, 122.0, 21.1, 11.5. GC-MS (70 eV): *m*/*z* 229 ([M^+^], 63), 168 (100), 152 (59), 128 (32), 115 (36), 91 (29), 77 (24). Anal. Calcd for C_14_H_15_NO_2_ (229.28): C, 73.34; H, 6.59; N, 6.11. Found: C, 73.38; H, 6.63; N, 6.14.

#### 1-Methoxy-4-((3*E*,5*E*)-6-nitroocta-1,3,5-trien-4-yl)benzene **4b** (Major Regioisomer)

Flash chromatography on silica gel using hexane/EtOAc = 98:2 as eluent yielded a 90:10 mixture of **4b** and **5b** (86 mg, 66% yield) as a pale yellow oil. Major regioisomer **4b**. IR (cm^–1^, neat): 832, 1030, 1175, 1244, 1333, 1511, 1604. ^1^H NMR (400 MHz, CDCl_3_) δ: 7.76 (s, 1H), 7.28 (d, 2H, *J* = 8.9 Hz), 6.89 (d, 2H, *J* = 8.9 Hz), 6.63–6.44 (m, 2H), 5.45 (dd, 1H, *J* = 16.0, 1.4 Hz), 5.33 (dd, 1H, *J* = 9.5, 1.3 Hz), 3.82 (s, 3H), 2.43 (q, 2H, *J* = 7.4 Hz), 0.97 (t, 3H, *J* = 7.4 Hz). ^13^C{^1^H} NMR (100 MHz, CDCl_3_) δ: 160.0, 155.8, 133.5, 131.3, 131.1, 130.7, 128.7, 128.1, 121.3, 114.4, 55.6, 21.4, 11.8. GC-MS (70 eV): *m*/*z* 259 ([M^+^], 84), 215 (51), 198 (100), 183 (47), 65 (27), 153 (31), 128 (24), 115 (26). Anal. Calcd for C_15_H_17_NO_3_ (259.31): C, 69.48; H, 6.61; N, 5.40. Found: C, 69.52; H, 6.57; N, 5.42.

#### 2-((3*E*,5*E*)-6-Nitroocta-1,3,5-trien-4-yl)thiophene **4c** (Major Regioisomer)

Flash chromatography on silica gel using hexane/EtOAc = 98:2 as eluent yielded a 90:10 mixture of **4c** and **5c** (67 mg, 57% yield) as a pale yellow oil. Major regioisomer **4c**. IR (cm^–1^, neat): 694, 828, 909, 1329, 1523, 1612, 1656. ^1^H NMR (400 MHz, CDCl_3_) δ: 7.65 (s, 1H), 7.25 (dd, 1H, *J* = 3.9, 0.8 Hz), 7.01–6.97 (m, 1H), 6.94 (dd, 1H, *J* = 3.6, 1.0 Hz), 6.68 (d, 1H, *J* = 11.6 Hz), 6.47–6.33 (m, 1H), 5.48 (dd, 1H, *J* = 16.7, 0.7 Hz), 5.33 (dd, 1H, *J* = 10.1, 0.7 Hz), 2.53 (q, 2H, *J* = 7.4 Hz), 1.05 (t, 3H, *J* = 7.4 Hz). ^13^C{^1^H} NMR (100 MHz, CDCl_3_) δ: 156.4, 142.2, 132.8, 131.2, 129.3, 129.0, 127.8, 125.6, 125.5, 121.4, 21.2, 11.8. GC-MS (70 eV): *m*/*z* 235 ([M^+^], 76), 174 (100), 147 (26), 128 (35), 115 (33), 97 (24), 77 (19). Anal. Calcd for C_12_H_13_NO_2_S (235.30): C, 61.25; H, 5.57; N, 5.95; S, 13.63. Found: C, 61.29; H, 5.60; N, 5.89; S, 13.67.

#### 2-((3*E*,5*E*)-6-Nitrodeca-1,3,5-trien-4-yl)naphthalene **4e** (Major Regioisomer)

Flash chromatography on silica gel using hexane/EtOAc = 98:2 as eluent yielded a 90:10 mixture of **4e** and **5e** (89 mg, 58% yield) as a pale yellow oil. Major regioisomer **4e**. IR (cm^–1^, neat): 464, 751, 812, 1325, 1430, 1467, 1519, 3054. ^1^H NMR (400 MHz, CDCl_3_) δ: 7.96 (s, 1H), 7.89–7.83 (m, 3H), 7.76 (d, 1H, *J* = 1.4 Hz), 7.58–7.49 (m, 3H), 6.84 (d, 1H, *J* = 11.0 Hz), 6.70–6.58 (m, 1H), 5.58 (d, 1H, *J* = 16.7 Hz), 5.45 (d, 1H, *J* = 10.1 Hz), 2.45–2.38 (m, 2H), 1.42–1.33 (m, 2H), 1.15–1.05 (m, 2H), 0.70 (t, 3H, *J* = 7.3 Hz). ^13^C{^1^H} NMR (100 MHz, CDCl_3_) δ: 154.9, 136.0, 133.6, 133.3, 133.2, 133.1, 130.5, 128.8, 128.5, 128.2, 127.6, 126.7, 126.5, 126.1, 124.2, 122.2, 29.1, 27.4, 22.3, 13.4. GC-MS (70 eV): *m*/*z* 307 ([M^+^], 50), 264 (37), 218 (100), 202 (92), 165 (14), 41 (14). Anal. Calcd for C_20_H_21_NO_2_ (307.39): C, 78.15; H, 6.89; N, 4.56. Found: C, 78.19; H, 6.92; N, 4.59.

#### 1-Methyl-4-((3*E*,5*E*)-6-nitro-8-phenylocta-1,3,5-trien-4-yl)benzene **4i**

Flash chromatography on silica gel using hexane/EtOAc = 98:2 as eluent yielded **4i** (96 mg, 60% yield) as a pale yellow oil. IR (cm^–1^, neat): 699, 821, 915, 1330, 1520, 3025. ^1^H NMR (400 MHz, CDCl_3_) δ: 7.89 (s, 1H), 7.29–7.07 (m, 7H), 6.93–6.89 (m, 2H), 6.62–6.45 (m, 2H), 5.47 (dd, 1H, *J* = 16.0, 1.7 Hz), 5.37 (dd, 1H, *J* = 9.4, 1.6 Hz), 2.66 (s, 4H), 2.37 (s, 3H). ^13^C{^1^H} NMR (100 MHz, CDCl_3_) δ: 153.1, 140.1, 138.4, 136.0, 133.6, 133.0, 132.9, 131.8, 129.5, 128.4, 128.3, 126.8, 126.3, 122.0, 33.0, 29.9, 21.2. GC-MS (70 eV): *m*/*z* 319 ([M^+^], 7), 227 (100), 182 (92), 165 (50), 152 (17), 91 (58). Anal. Calcd for C_21_H_21_NO_2_ (319.40): C, 78.97; H, 6.63; N, 4.39. Found: C, 79.01; H, 6.60; N, 4.42.

#### ((3*E*,5*E*)-10-Chloro-6-nitrodeca-1,3,5-trien-4-yl)benzene **4l** (Major Regioisomer)

Flash chromatography on silica gel using hexane/EtOAc = 98:2 as eluent yielded a 90:10 mixture of **4l** and **5l** (92 mg, 63% yield) as a pale yellow oil. Major regioisomer **4l**. IR (cm^–1^, neat): 700, 1326, 1519, 1640. ^1^H NMR (400 MHz, CDCl_3_) δ: 7.93 (s, 1H), 7.43–7.27 (m, 5H), 6.70–6.51 (m, 2H), 5.52 (dd, 1H, *J* = 15.9, 1.5 Hz), 5.42 (dd, 1H, *J* = 9.4, 1.5 Hz), 3.34 (t, 2H, *J* = 6.2 Hz), 2.40–2.34 (m, 2H), 1.62–1.44 (m, 4H). ^13^C{^1^H} NMR (100 MHz, CDCl_3_) δ: 153.7, 138.8, 133.7, 133.6, 132.8, 131.0, 128.9, 128.5, 126.8, 122.6, 44.1, 32.0, 26.9, 24.4. GC-MS (70 eV): *m*/*z* 291 (29), 214 (43), 168 (100), 167 (70), 165 (53), 153 (32), 152 (59), 115 (22), 91 (20), 41 (21). Anal. Calcd for C_16_H_18_ClNO_2_ (291.77): C, 65.86; H, 6.22; N, 4.80. Found: C, 65.91; H, 6.19; N, 4.83.

#### Methyl (7*E*,9*E*)-7-Nitro-9-phenyldodeca-7,9,11-trienoate **4n** (Major Regioisomer)

Flash chromatography on silica gel using hexane/EtOAc = 98:2 as eluent yielded a 90:10 mixture of **4n** and **5n** (94 mg, 57% yield) as a yellow oil. Major regioisomer **4n** IR (cm^–1^, neat): 697, 732, 1331, 1521, 1734. ^1^H NMR (400 MHz, CDCl_3_) δ: 7.87 (s, 1H), 7.40–7.27 (m, 5H), 6.68–6.49 (m, 2H), 5.50 (dd, 1H, *J* = 16.3, 1.5 Hz), 5.39 (dd, 1H, *J* = 9.8, 1.5 Hz), 3.63 (s, 3H), 2.37–2.31 (m, 2H), 2.16 (t, 2H, *J* = 7.5 Hz), 1.49–1.30 (m, 4H), 1.15–1.04 (m, 2H). ^13^C{^1^H} NMR (100 MHz, CDCl_3_) δ: 174.0, 154.2, 138.8, 133.7, 133.2, 132.9, 130.7, 128.8, 128.4, 126.8, 122.3, 51.5, 33.7, 28.7, 27.4, 26.6, 24.2. GC-MS (70 eV): *m*/*z* 294 (10), 220 (81), 210 (80), 168 (100), 167 (94), 115 (40), 87 (41), 55 (35). Anal. Calcd for C_19_H_23_NO_4_ (329.40): C, 69.28; H, 7.04; N, 4.25. Found: C, 69.33; H, 7.07; N, 4.21.

## Data Availability

The data underlying this study are available in the published article and its online Supporting Information.

## Supporting Information Available

Full characterization of compounds **1**; copies of ^1^H NMR and ^13^C{1H} NMR spectra for compounds **1**, **3**, and **4** (PDF)

## References

[ref1] aChiurchiù E.; Xhafa S.; Ballini R.; Maestri G.; Protti S.; Palmieri A. Diastereoselective Isomerization of (*E*)-β-Nitroenones into β-Nitro-β,γ-Unsaturated Ketones under Microwave Conditions. Adv. Synth. Catal. 2020, 362 (21), 4680–4686. 10.1002/adsc.202000747.

[ref2] aRaviola C.; Carrera C.; Serra M.; Palmieri A.; Lupidi G.; Maestri G.; Protti S. Visible-Light-Driven Competitive Stereo- and Regioisomerization of (*E*)-β-Nitroenones. ChemPhotoChem. 2021, 5 (9), 871–875. 10.1002/cptc.202100081.

[ref3] aLübbesmeyer M.; Mackay E. G.; Raycroft M. A. R.; Elfert J.; Pratt D. A.; Studer A. Base-Promoted C-C Bond Activation Enables Radical Allylation with Homoallylic Alcohols. J. Am. Chem. Soc. 2020, 142 (5), 2609–2616. 10.1021/jacs.9b12343. 31941267 PMC7021447

[ref4] aLu Z.; Zhang X.; Guo Z.; Chen Y.; Mu T.; Li A. Total Synthesis of Aplysiasecosterol A. J. Am. Chem. Soc. 2018, 140 (29), 9211–9218. 10.1021/jacs.8b05070. 29939021

[ref5] aTan B.; Chua P. J.; Li Y. X.; Zhong G. F. Organocatalytic Asymmetric Tandem Michael-Henry Reactions: A Highly Stereoselective Synthesis of Multifunctionalized Cyclohexanes with Two Quaternary Stereocenters. Org. Lett. 2008, 10 (12), 2437–2440. 10.1021/ol8007183. 18489178

[ref6] aSalvatore S. R.; Rowart P.; Schopfer F. J. Mass Spectrometry-Based Study Defines the Human Urine Nitrolipidome. Free Radic. Biol. Med. 2021, 162, 327–337. 10.1016/j.freeradbiomed.2020.10.305. 33131723 PMC10895545

[ref7] Kalita P. K.; Phukan P. Facile Chemoselective Carbonyl Allylation of Chalcones with Allyltributylstannane Catalyzed by CuI. Tetrahedron Lett. 2013, 54 (33), 4442–4445. 10.1016/j.tetlet.2013.06.037.

[ref8] aAugé J.; Lubin-Germain N.; Marque S.; Seghrouchni L. Indium-catalyzed Barbier Allylation Reaction. J. Organomet. Chem. 2003, 679 (1), 79–83. 10.1016/S0022-328X(03)00515-1.

[ref9] Schneider U.; Kobayashi S. Catalytic Activation of Pinacolyl Allylboronate with Indium(I): Development of a General Catalytic Allylboration of Ketones. Angew. Chem., Int. Ed. 2007, 46 (31), 5909–5912. 10.1002/anie.200700899. 17577908

[ref10] Hema K.; Ravi A.; Raju C.; Pathan J. R.; Rai R.; Sureshan K. M. Topochemical Polymerizations for the Solid-state Synthesis of Organic Polymers. Chem. Soc. Rev. 2021, 50, 4062–4099. 10.1039/D0CS00840K. 33543741

[ref11] aFang Y.; Yang Z.; Park H. Straightforward and Facile Synthesis of a Bioactive Component from Zingiber cassumunar Roxb. Synth. Commun. 2014, 44 (9), 1212–1217. 10.1080/00397911.2013.846380.

[ref12] Posner G. H.; Shulman-Roskes E. M.; Oh C. H.; Carry J.-C.; Green J. V.; Clark A. B.; Dai H.; Anjeh T. E. N. BF_3_·OEt_2_ Promotes Fast, Mild, Clean and Regioselective Dehydration of Tertiary Alcohols. Tetrahedron Lett. 1991, 32 (45), 6489–6492. 10.1016/0040-4039(91)80200-P.

[ref13] Johns B. A.; Grant C. M.; Marshall J. Synthesis and Utilization of Indium (I) Iodide for in Situ Formation of Enantioenriched Allenylindium Reagents and Their Addition to Aldehydes: (2R, 3S, 4S)-1-(tert-Butyldiphenylsilyloxy)-2,4-dimethyl-5-hexyn-3-ol. Org. Synth. 2003, 79, 59–71. 10.1002/0471264180.os079.08.

[ref14] aBallini R.; Fiorini D.; Palmieri A. Nitroalkanes and Ethyl Glyoxalate as Common Precursors for the Preparation of Both β-Keto Esters and α,β-Unsaturated Esters. Tetrahedron Lett. 2004, 45, 7027–7029. 10.1016/j.tetlet.2004.07.141.

