# Supplementary material for: Overcoming the Usual Reactivity of β-Nitroenones: Synthesis of Polyfunctionalized Homoallylic Alcohols and Conjugated Nitrotriene Systems
Source: J Org Chem. 2023 Mar 16;88(7):4770–7. doi: 10.1021/acs.joc.2c02669 (PMC10088019; doi:10.1021/acs.joc.2c02669)

# Overcoming the Usual Reactivity of β-Nitroenones: Synthesis of Polyfunctionalized Homoallylic Alcohols and Conjugated Nitrotriene Systems

Lixia Yuan,[b,a] Liudmila Kachalova,[a] Muhammad E. I. Khan,[a] Roberto Ballini,[a] Marino Petrini,*[a] and Alessandro Palmieri*[a]

[a] Green Chemistry Group, School of Sciences and Technology, Chemistry Division, University of Camerino ChIP Research Center, Via Madonna delle Carceri, 62032 Camerino (MC), Italy E-mail: marino.petrini@unicam.it; alessandro.palmieri@unicam.it

[b] Institute of BioPharmaceutical Research, Liaocheng University, Liaocheng 252059, People's Republic of China

## Table of contents

1. Large-scale reaction investigations ........ S2
2. Spectroscopic data of compounds **1a-o** ........ S3
3. Copy of $^{1}$H NMR and $^{113}$C{$^{1}$H} NMR of compounds **1** ........ S9
4. Copy of $^{1}$H NMR and $^{113}$C{$^{1}$H} NMR of compounds **3** ........ S39
5. Copy of $^{1}$H NMR and $^{13}$C{$^{1}$H} NMR of compounds **4** ........ S69
6. NOESY in $CDCl_3$ of Compound **4a** ........ S83

## 1. Large-scale reaction investigations.

**Synthesis of compound 3a on 5 mmol scale.** An oven-dried round bottom flask with a magnetic stir bar, and maintained under inert atmosphere, was charged with the appropriate β-nitroenone **1a** (5 mmol, 1.026 g), dry THF (25 mL), InI (0.25 mmol, 60 mg) and the allylboronic acid pinacol ester **2c** (7.5 mmol, 1.407 mL). The resulting mixture was vigorously stirred at 40°C for 7 hours, then was diluted with dichloromethane (100 mL) and treated with a saturated aqueous solution of $NaHCO_3$ (40 mL). After phase separation, the aqueous one was extracted with dichloromethane (3 x 50 mL) and the combined organic layers were dried with dry $Na_2SO_4$. Finally, the solution was filtered and concentrated in vacuo to give the crude product **3a**, which was purified by flash column chromatography (hexane/ethyl acetate=95:5) yielding the pure **3a** in 95% yield (1.174g).

**Conversion of 3a into trienes 4a and 5a on 2 mmol scale.** $BF_3{\cdot}Et_2O$ (3 mmol, 0.370 mL) was added dropwise and at -10°C to a stirred solution of **3a** (2 mmol, 0.495 g) in dichloromethane (20 mL). The reaction was stirred at the same temperature for 5 hours, then diluted with dichloromethane (20 mL) and treated with a saturated aqueous solution of $NaHCO_3$ (20 mL). After phase separation, the aqueous one was extracted with dichloromethane (3 x 30 mL) and the combined organic layers were dried with dry $Na_2SO_4$. Finally, the solution was filtered and concentrated in vacuo to give the crude regioisomeric products **4a** and **5a,** which were purified by flash column chromatography (hexane/ethyl acetate=98:2) yielding **4a** and **5a** in 90:10 regioisomeric ratio and in 69% of overall yield (0.316 g).

## 2. Spectroscopic data of compounds **1**.

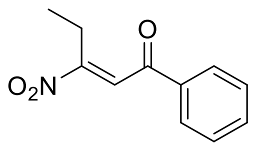

(*E*)-3-Nitro-1-phenylpent-2-en-1-one **1a**. Flash chromatography on silica gel using (hexane/EtOAc = 95:5) as eluent yielded **1a** (291 mg, 71% yield) as yellow solid. M.p.: 41-43°C. IR ($cm^{-1}$, neat): 733, 1337, 1449, 1528, 1596, 1634, 1679. $^{1}H$ NMR (400 MHz, $CDCl_3$) δ: 7.99-7.97 (m, 2H), 7.95 (s, 1H), 7.67-7.64 (m, 1H), 7.58-7.52 (m, 2H), 2.93 (q, 2H, *J* = 7.4 Hz), 1.21 (t, 3H, *J* = 7.4 Hz). $^{13}C\{^{1}H\}$ NMR (100 MHz, $CDCl_3$) δ: 189.7, 162.8, 136.7, 134.4, 129.1, 128.7, 124.6, 21.4, 12.6. GC-MS (70eV): *m/z*: 159 (60), 144 (14), 105 (100), 77 (68), 51 (20). Anal. Calcd. For $C_{11}H_{11}NO_3$ (205.21): C, 64.38; H, 5.40; N, 6.83. Found: C, 64.42; H, 5.37; N, 6.80.

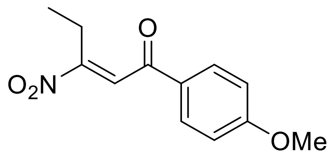

(*E*)-1-(4-Methoxyphenyl)-3-nitropent-2-en-1-one **1b**. Flash chromatography on silica gel using (hexane/EtOAc = 95:5) as eluent yielded **1b** (343 mg, 73% yield) as yellow solid. M.p.: 57-59°C. IR ($cm^{-1}$, neat): 763, 849, 1177, 1520, 1589, 1628, 1674. $^{1}H$ NMR (400 MHz, $CDCl_3$) δ: 7.95 (d, 2H, *J* = 8.9 Hz), 7.92 (s, 1H), 6.99 (d, 2H, *J* = 8.9 Hz), 3.90 (s, 3H), 2.90 (q, 2H, *J* = 7.4 Hz), 1.20 (t, 3H, *J* = 7.4 Hz). $^{13}C\{^{1}H\}$ NMR (100 MHz, $CDCl_3$) δ: 188.1, 164.7, 162.0, 131.2, 129.8, 125.1, 114.3, 55.7, 21.4, 12.6. GC-MS (70eV): *m/z*: 135 (100), 107 (9), 92 (14), 77 (18). Anal. Calcd. For $C_{12}H_{13}NO_4$ (235.24): C, 61.27; H, 5.57; N, 5.95. Found: C, 61.20; H, 5.54; N, 5.93.

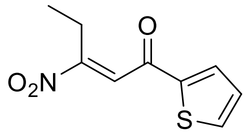

(*E*)-3-Nitro-1-(thiophen-2-yl)pent-2-en-1-one **1c**. Flash chromatography on silica gel using (hexane/EtOAc = 90:10) as eluent yielded **1c** (283 mg, 67% yield) as orange solid. M.p.: 51-53°C. IR ($cm^{-1}$, neat): 727, 1338, 1411, 1530, 1623, 1670. $^{1}H$ NMR (400 MHz, $CDCl_3$) δ: 7.85 (s, 1H), 7.81 (dd, 1H, *J* = 3.9, 1.1 Hz), 7.79 (dd, 1H, *J* = 4.9, 1.1 Hz), 7.20 (dd, 1H, *J* = 4.9, 3.9 Hz), 3.04 (q, 2H, *J* = 7.4 Hz), 1.21 (t, 3H, *J* = 7.4 Hz). $^{13}C\{^{1}H\}$ NMR (100 MHz, $CDCl_3$) δ: 181.2, 163.9, 144.9, 136.2, 133.5,

128.8, 123.0, 21.4, 12.5. GC-MS (70eV): *m/z*: 165 (7), 111 (100), 83 (11), 39 (19). Anal. Calcd. For $C_9H_9NO_3S$ (211.24): C, 51.17; H, 4.29; N, 6.63; S, 15.18. Found: C, 51.14; H, 4.32; N, 6.60; S, 15.15.

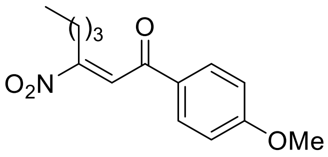


(*E*)-1-(4-Methoxyphenyl)-3-nitrohept-2-en-1-one **1d**. Flash chromatography on silica gel using (hexane/EtOAc = 95:5) as eluent yielded **1d** (369 mg, 70% yield) as yellow solid. M.p.: 29-31°C. IR (cm$^{-1}$, neat): 589, 599, 838, 1169, 1422, 1530, 1595, 1626, 1674. $^1$H NMR (400 MHz, $CDCl_3$) δ: 7.94 (d, 2H, *J* = 9.0 Hz), 7.92 (s, 1H), 6.98 (d, 2H, *J* = 8.9 Hz), 3.90 (s, 3H), 2.94-2.86 (m, 2H), 1.60-1.49 (m, 2H), 1.43-1.31 (m, 2H), 0.89 (t, 3H, *J* = 7.3 Hz). $^{13}$C{$^1$H} NMR (100 MHz, $CDCl_3$) δ: 188.0, 164.6, 161.3, 131.2, 129.9, 125.1, 114.3, 55.6, 30.1, 27.3, 22.4, 13.6. GC-MS (70eV): *m/z*: 135 (100), 107 (11), 92 (15), 77 (18). Anal. Calcd. For $C_{14}H_{17}NO_4$ (263.29): C, 63.87; H, 6.51; N, 5.32. Found: C, 63.91; H, 6.53; N, 5.29.

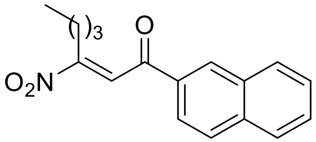


(E)-1-(Naphthalen-2-yl)-3-nitrohept-2-en-1-one **1e**. Flash chromatography on silica gel using (hexane/EtOAc = 97:3) as eluent yielded **1e** (419 mg, 74% yield) as orange waxy solid. IR (cm$^{-1}$, neat): 473, 758, 1334, 1531, 1625, 1675. $^1$H NMR (400 MHz, $CDCl_3$) δ: 8.47 (s, 1H), 8.13 (s, 1H), 8.08-7.87 (m, 4H), 7.72-7.57 (m, 2H), 3.00-2.95 (m, 2H), 1.64-1.54 (m, 2H), 1.46-1.35 (m, 2H), 0.92 (t, 3H, *J* = 7.3 Hz). $^{13}$C{$^1$H} NMR (100 MHz, $CDCl_3$) δ: 189.5, 162.0, 136.1, 134.2, 132.4, 131.2, 129.8, 129.4, 129.2, 127.9, 127.3, 124.8, 123.5, 30.1, 27.4, 22.5, 13.7. GC-MS (70eV): *m/z*: 254 (24), 212 (31), 197 (32), 155 (100), 127 (68), 69 (53). Anal. Calcd. For $C_{17}H_{17}NO_3$ (283.33): C, 72.07; H, 6.05; N, 4.94. Found: C, 72.11; H, 6.02; N, 4.91.

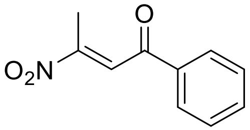


(*E*)-3-Nitro-1-phenylbut-2-en-1-one **1f**. Flash chromatography on silica gel using (hexane/EtOAc = 95:5) as eluent yielded **1f** (252 mg, 66% yield) as yellow solid. M.p.: 26-28 °C. IR (cm$^{-1}$, neat): 616, 687, 724, 960, 1328, 1449, 1528, 1596, 1635, 1680. $^1$H NMR (400 MHz, $CDCl_3$) δ: 8.03 (q, 1H, *J* = 1.2 Hz), 7.97 (dd, 2H, *J* = 8.4, 1.3 Hz), 7.69-7.62 (m,

1H), 7.53 (t, 2H, $J$ = 7.7 Hz), 2.50 (d, 3H, $J$ = 1.2 Hz). $^{13}C\{^1H\}$ NMR (100 MHz, $CDCl_3$) δ: 189.8, 157.6, 136.7, 134.5, 129.1, 128.7, 125.0, 14.3. GC-MS (70eV): *m/z*: 145 (54), 105 (100), 77 (69), 51 (21), 39 (7). Anal. Calcd. For $C_{10}H_9NO_3$ (191.19): C, 62.82; H, 4.75; N, 7.33. Found: C, 62.79; H, 4.78; N, 7.30.

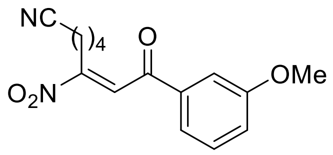


(*E*)-8-(3-Methoxyphenyl)-6-nitro-8-oxooct-6-enenitrile **1g**. Flash chromatography on silica gel using (hexane/EtOAc = 85:15) as eluent yielded **1g** (409 mg, 71% yield) as orange waxy solid. IR (cm$^{-1}$, neat): 727, 907, 1263, 1429, 1534, 1582, 1596, 1679, 2253. $^1H$ NMR (400 MHz, $CDCl_3$) δ: 8.02 (s, 1H), 7.37-7.56 (m, 3H), 7.20 (dd, 1H, $J$ = 8.2, 2.7 Hz), 3.87 (s, 3H), 2.96 (t, 2H, $J$ = 7.1 Hz), 2.40 (t, 2H, $J$ = 6.8 Hz), 1.82-1.71 (m, 4H). $^{13}C\{^1H\}$ NMR (100 MHz, $CDCl_3$) δ: 189.2, 160.7, 160.2, 137.9, 130.2, 125.3, 121.5, 121.3, 119.3, 112.4, 55.6, 27.0, 26.8, 24.9, 16.8. GC-MS (70eV): *m/z*: 241 (38), 201 (28), 187 (100), 135 (32), 107 (20), 92 (19), 77 (33). Anal. Calcd. For $C_{15}H_{16}N_2O_4$ (288.30): C, 62.49; H, 5.59; N, 9.72. Found: C, 62.53 H, 5.62; N, 9.69.

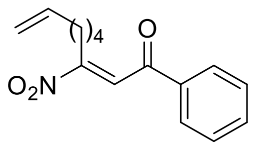


(*E*)-3-Nitro-1-phenylnona-2,8-dien-1-one **1h**. Flash chromatography on silica gel using (hexane/EtOAc = 97:3) as eluent yielded **1h** (399 mg, 77% yield) as yellow oil. IR (cm$^{-1}$, neat): 615, 688, 732, 911, 1002, 1334, 1449, 1530, 1596, 1639, 1678. $^1H$ NMR (400 MHz, $CDCl_3$) δ: 7.99-7.95 (m, 3H), 7.69-7.63 (m, 1H), 7.54 (t, 2H, $J$ = 7.7 Hz), 5.82-5.70 (m, 1H), 5.04-4.88 (m, 2H), 2.97-2.91 (m, 2H), 2.06 (q, 2H, $J$ = 7.2 Hz), 1.67-1.55 (m, 2H), 1.52-1.41 (m, 2H). $^{13}C\{^1H\}$ NMR (100 MHz, $CDCl_3$) δ: 189.7, 161.8, 138.2, 136.8, 134.4, 129.1, 128.7, 124.8, 114.8, 33.2, 28.4, 27.5, 27.4. GC-MS (70eV): *m/z*: 212 (7), 183 (28), 157 (53), 144 (24), 128 (35). 105 (100), 77 (67). Anal. Calcd. For $C_{15}H_{17}NO_3$ (259.31): C, 69.48; H, 6.61; N, 5.40. Found: C, 69.52; H, 6.58; N, 5.37.

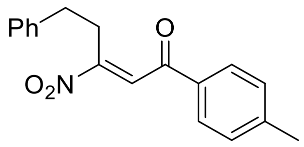

($E$)-3-Nitro-5-phenyl-1-(p-tolyl)pent-2-en-1-one **1i**. Flash chromatography on silica gel using (hexane/EtOAc = 97:3) as eluent yielded **1i** (402 mg, 68% yield) as yellow solid. M.p.: 63-65 °C. IR ($cm^{-1}$, neat): 509, 591, 694, 742, 829, 973, 1235, 1328, 1526, 1599, 1620, 1671. $^{1}H$ NMR (400 MHz, $CDCl_3$) δ: 7.98 (bs, 1H), 7.76 (d, 2H, $J$ = 8.3 Hz), 7.29 (d, 2H, $J$ = 8.3 Hz), 7.24-7.16 (m, 4H), 7.14-7.07 (m, 1H), 3.26-3.19 (m, 2H), 2.94-2.87 (m, 2H), 2.45 (s, 3H). $^{13}C\{^{1}H\}$ NMR (100 MHz, $CDCl_3$) δ: 189.1, 160.1, 145.6, 139.7, 134.2, 129.7, 128.8, 128.6, 128.5, 126.5, 126.1, 34.1, 29.7, 21.9. GC-MS (70eV): $m/z$: 248 (100), 205 (22), 171 (27), 128 (47), 119 (36), 91 (45), 65 (18). Anal. Calcd. For $C_{18}H_{17}NO_3$ (295.34): C, 73.20; H, 5.80; N, 4.74. Found: C, 73.24; H, 5.77; N, 4.76.

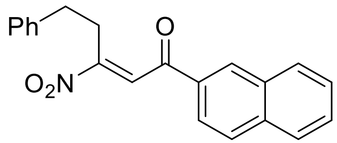

($E$)-1-(Naphthalen-2-yl)-3-nitro-5-phenylpent-2-en-1-one **1j**. Flash chromatography on silica gel using (hexane/EtOAc = 98:2) as eluent yielded **1j** (497 mg, 75% yield) as yellow solid. M.p.: 94-96 °C. IR ($cm^{-1}$, neat): 479, 697, 748, 766, 836, 1123, 1339, 1528, 1615, 1667. $^{1}H$ NMR (400 MHz, $CDCl_3$) δ: 8.28 (s, 1H), 8.15 (s, 1H), 8.01-7.88 (m, 4H), 7.70-7.57 (m, 2H), 7.22-7.11 (m, 4H), 6.99 (t, 1H, $J$ = 7.0 Hz), 3.33-3.26 (m, 2H), 2.98-2.91 (m, 2H). $^{13}C\{^{1}H\}$ NMR (100 MHz, $CDCl_3$) δ: 189.4, 160.3, 139.6, 136.0, 134.0, 132.3, 131.3, 129.8, 129.4, 129.0, 128.6, 128.5, 127.9, 127.2, 126.5, 126.2, 123.4, 34.1, 29.7. GC-MS (70eV): $m/z$: 155 (100), 127 (53), 91 (19). Anal. Calcd. For $C_{21}H_{17}NO_3$ (331.37): C, 76.12; H, 5.17; N, 4.23. Found: C, 76.16; H, 5.20; N, 4.20.

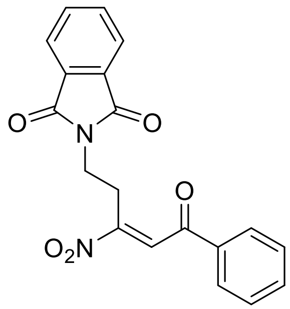

(*E*)-2-(3-Nitro-5-oxo-5-phenylpent-3-en-1-yl)isoindoline-1,3-dione **1k**. Flash chromatography on silica gel using (hexane/EtOAc = 85:15) as eluent yielded **1k** (427 mg, 61% yield) as yellow solid. M.p.: 146-149 °C. IR ($cm^{-1}$, neat): 528, 706, 688, 715, 1331, 1396, 1524, 1594, 1647, 1677, 1704. $^1$H NMR (400 MHz, $CDCl_3$) δ: 8.11 (s, 1H), 7.79-7.68 (m, 4H), 7.67-7.59 (m, 2H), 7.55-7.47 (m, 1H), 7.35 (t, 2H, *J* = 7.9 Hz), 4.07-4.01 (m, 2H), 3.31-3.25 (m, 2H). $^{13}$C{$^1$H} NMR (100 MHz, $CDCl_3$) δ: 189.1, 168.2, 157.0, 136.0, 134.4, 133.9, 131.9, 128.9, 128.6, 128.5, 123.3, 35.5, 27.0. GC-MS (70eV): *m/z*: 216 (85), 188 (19), 160 (42), 105 (100), 77 (51), 51 (17). Anal. Calcd. For $C_{19}H_{14}N_2O_5$ (350.33): C, 65.14; H, 4.03; N, 8.00. Found: C, 65.18; H, 4.01; N, 8.03.

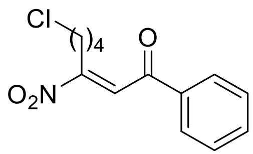

(*E*)-7-Chloro-3-nitro-1-phenylhept-2-en-1-one **1l**. Flash chromatography on silica gel using (hexane/EtOAc = 95:5) as eluent yielded **1l** (380 mg, 71% yield) as yellow oil. IR ($cm^{-1}$, neat): 687, 731, 1002, 1227, 1326, 1336, 1448, 1531, 1633, 1678. $^1$H NMR (400 MHz, $CDCl_3$) δ: 8.03 (s, 1H), 8.00-7.94 (m, 2H), 7.71-7.64 (m, 1H), 7.54 (t, 2H, *J* = 7.9 Hz), 3.55 (t, 2H, *J* = 6.4 Hz), 3.01-2.93 (m, 2H), 1.93-1.70 (m, 4H). $^{13}$C{$^1$H} NMR (100 MHz, $CDCl_3$) δ: 189.5, 161.2, 136.7, 134.6, 129.1, 128.7, 125.1, 44.2, 32.0, 26.9, 25.4. GC-MS (70eV): *m/z*: 221 (4), 157 (7), 105 (100), 77 (43), 51 (11). Anal. Calcd. For $C_{13}H_{14}ClNO_3$ (267.71): C, 58.33; H, 5.27; N, 5.23. Found: C, 58.29; H, 5.30; N, 5.21.

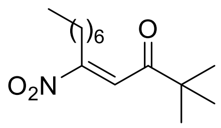

(*E*)-2,2-Dimethyl-5-nitrododec-4-en-3-one **1m**. Flash chromatography on silica gel using (hexane/EtOAc = 95:5) as eluent yielded **1m** (368 mg, 72% yield) as yellow oil. IR ($cm^{-1}$, neat): 578, 993, 1076, 1335, 1465, 1535, 1701. $^1$H NMR (400 MHz, $CDCl_3$) δ: 7.54 (s, 1H), 2.90-2.84 (m, 2H), 1.65-1.45 (m, 2H), 1.42-1.14 (m, 8H), 1.21 (s, 9H), 0.87 (t, 3H, *J* = 6.9 Hz). $^{13}$C{$^1$H} NMR (100 MHz, $CDCl_3$) δ: 204.9, 162.4, 122.5, 44.9, 31.6, 29.3, 28.8, 28.1, 27.3, 25.9, 22.6, 14.1. GC-MS (70eV): *m/z*: 255

(1, [M$^+$]), 199 (10), 151 (26), 143 (22), 81 (20), 57 (100), 41 (43), 29 (19). Anal. Calcd. For $C_{14}H_{25}NO_3$ (255.36): C, 65.85; H, 9.87; N, 5.49. Found: C, 65.88; H, 9.90; N, 5.52.

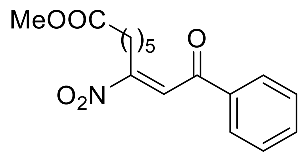

Methyl (*E*)-7-nitro-9-oxo-9-phenylnon-7-enoate **1n**. Flash chromatography on silica gel using (hexane/EtOAc = 90:10) as eluent yielded **1n** (403 mg, 66% yield) as yellow oil. IR (cm$^{-1}$, neat): 688, 732, 1334, 1450, 1531, 1678, 1734. $^1$H NMR (400 MHz, $CDCl_3$) δ: 7.99-7.94 (m, 3H), 7.69-7.62 (m, 1H), 7.53 (t, 2H, *J* = 7.9 Hz), 3.65 (s, 3H), 2.95-2.88 (m, 2H), 2.29 (t, 2H, *J* = 7.5 Hz), 1.69-1.54 (m, 4H), 1.45-1.33 (m, 2H). $^{13}$C{$^1$H} NMR (100 MHz, $CDCl_3$) δ: 189.6, 174.0, 161.7, 136.7, 134.5, 129.1, 128.7, 124.8, 51.5, 33.8, 28.7, 27.7, 27.4, 24.4. GC-MS (70eV): *m/z*: 271 (34), 198 (76), 105 (100), 77 (78). Anal. Calcd. For $C_{16}H_{19}NO_5$ (305.33): C, 62.94; H, 6.27; N, 4.59. Found: C, 62.98; H, 6.30; N, 4.61.

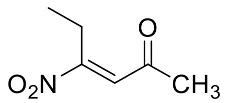

(*E*)-4-Nitrohex-3-en-2-one **1o**. Flash chromatography on silica gel using (hexane/EtOAc = 90:10) as eluent yielded **1o** (180 mg, 63% yield) as yellow oil. IR (cm$^{-1}$, neat): 557, 730, 1183, 1338, 1531, 1644, 1706. $^1$H NMR (400 MHz, $CDCl_3$) δ: 7.24 (s, 1H), 2.96 (q, 2H, *J* = 7.4 Hz), 2.40 (s, 3H), 1.15 (t, 3H, *J* = 7.4 Hz). $^{13}$C{$^1$H} NMR (100 MHz, $CDCl_3$) δ: 196.8, 163.0, 124.7, 32.5, 20.9, 12.4. GC-MS (70eV): *m/z*: 97 (36), 81 (11), 43 (100). Anal. Calcd. For $C_6H_9NO_3$ (143.14): C, 50.35; H, 6.34; N, 9.79. Found: C, 50.39; H, 6.37; N, 9.76.

3. Copy of $^{1}H$ NMR and $^{13}C\{^{1}H\}$ NMR of compounds **1**.

$^{1}H$ NMR (400 MHz, $CDCl_3$) Compound **1a**.

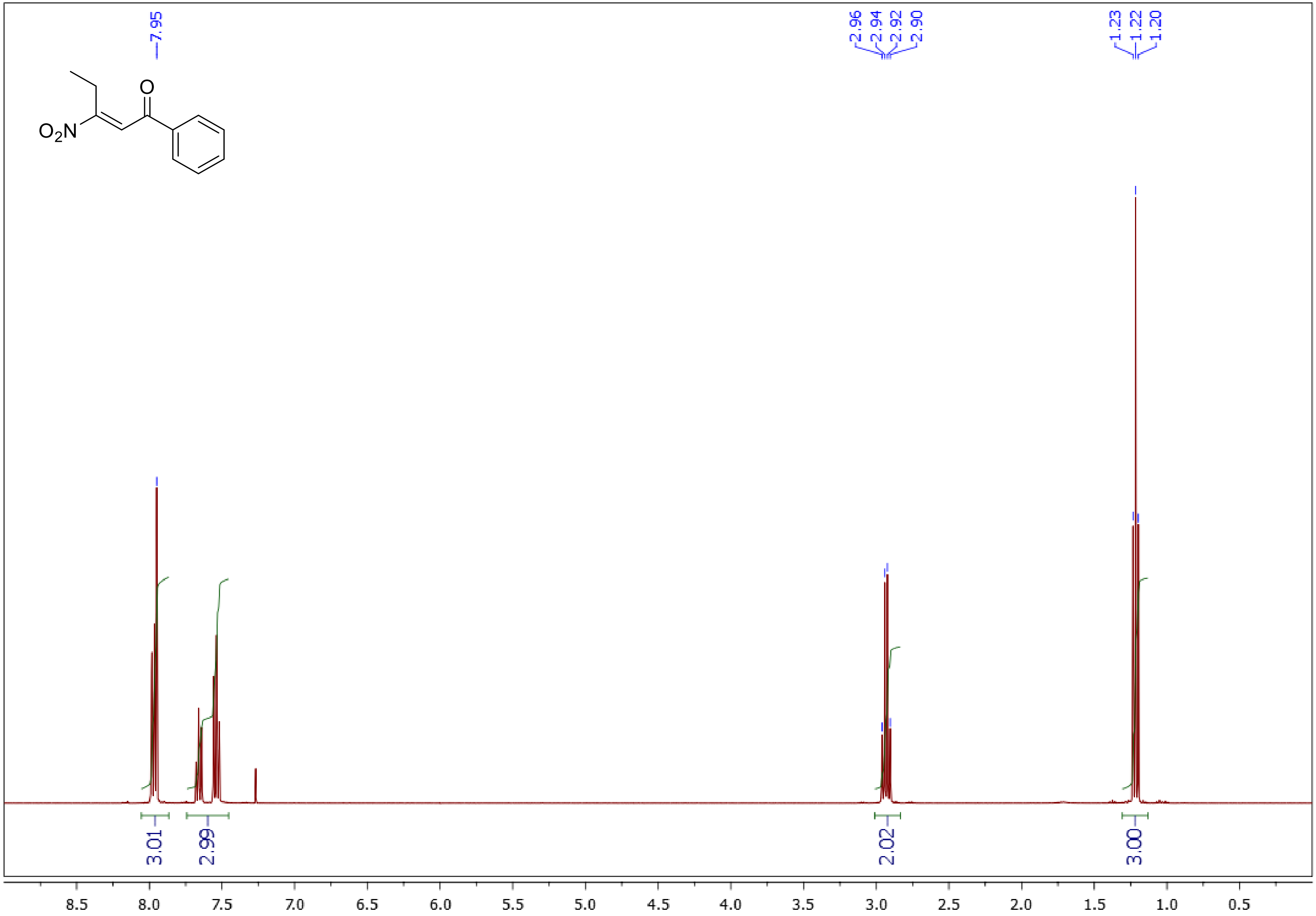

$^{13}C\{^1H\}$ NMR (100 MHz, $CDCl_3$) Compound **1a**.

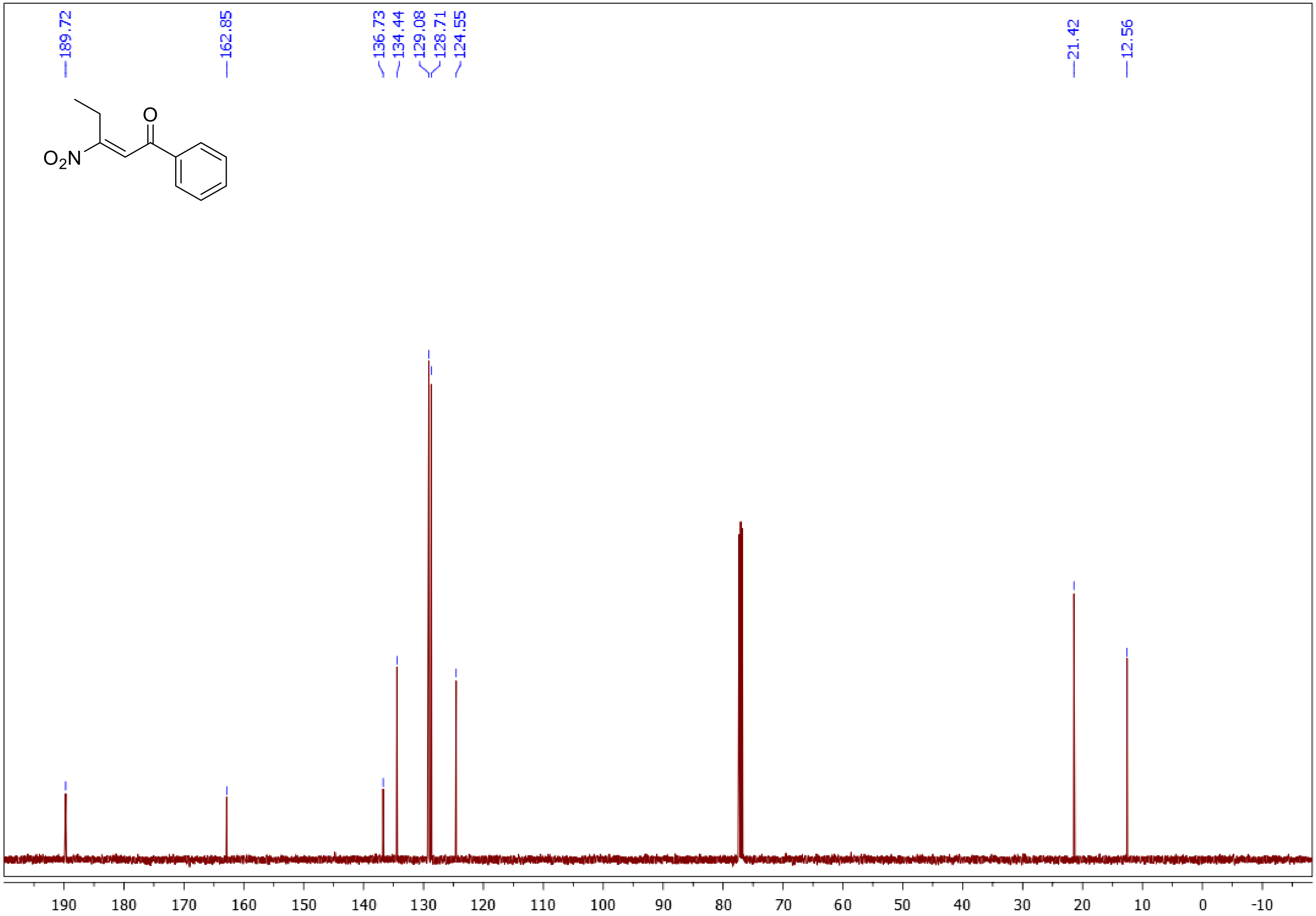

$^{1}H$ NMR (400 MHz, $CDCl_3$) Compound **1b**.

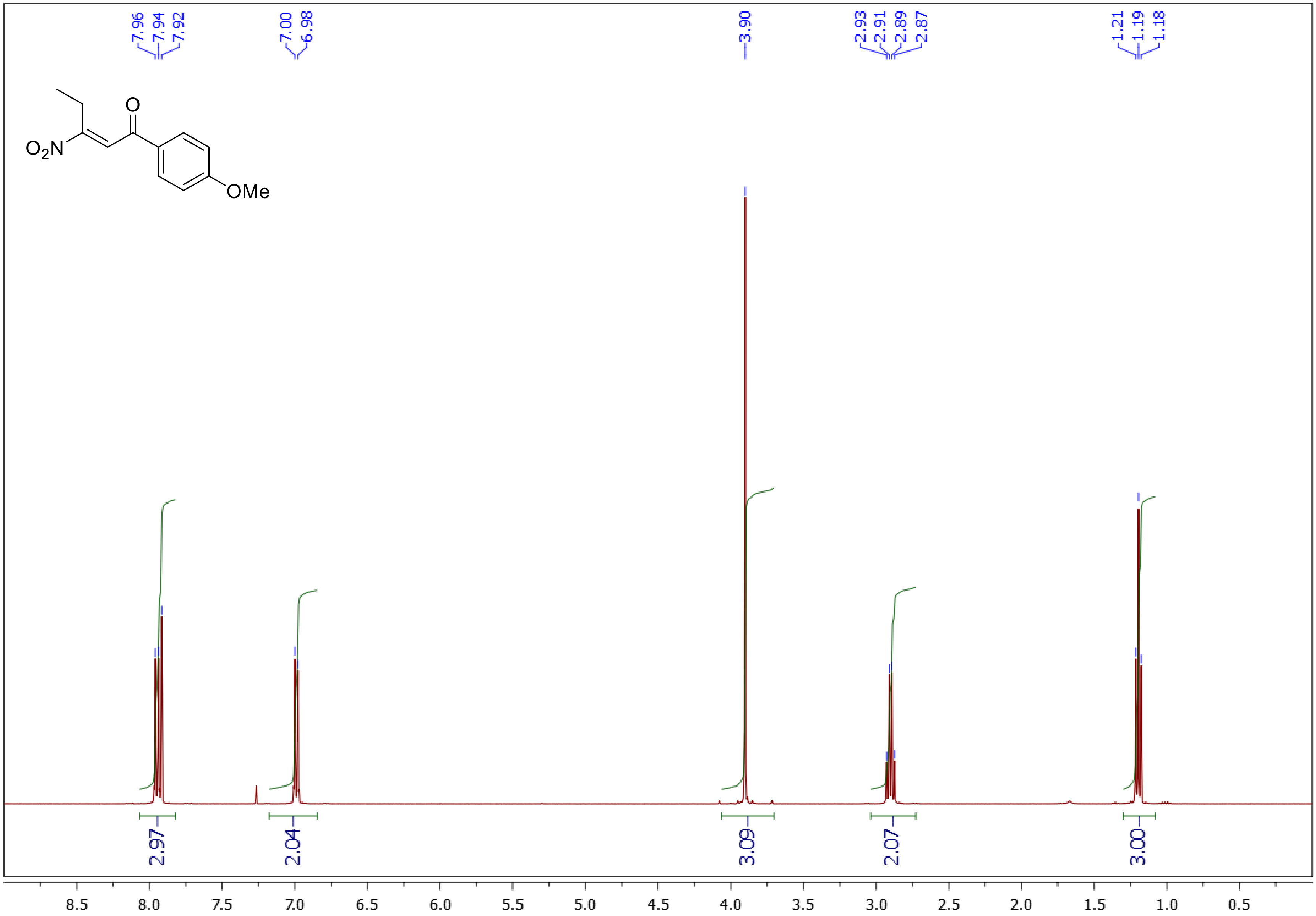

$^{13}C\{^{1}H\}$ NMR (100 MHz, $CDCl_3$) Compound **1b**.

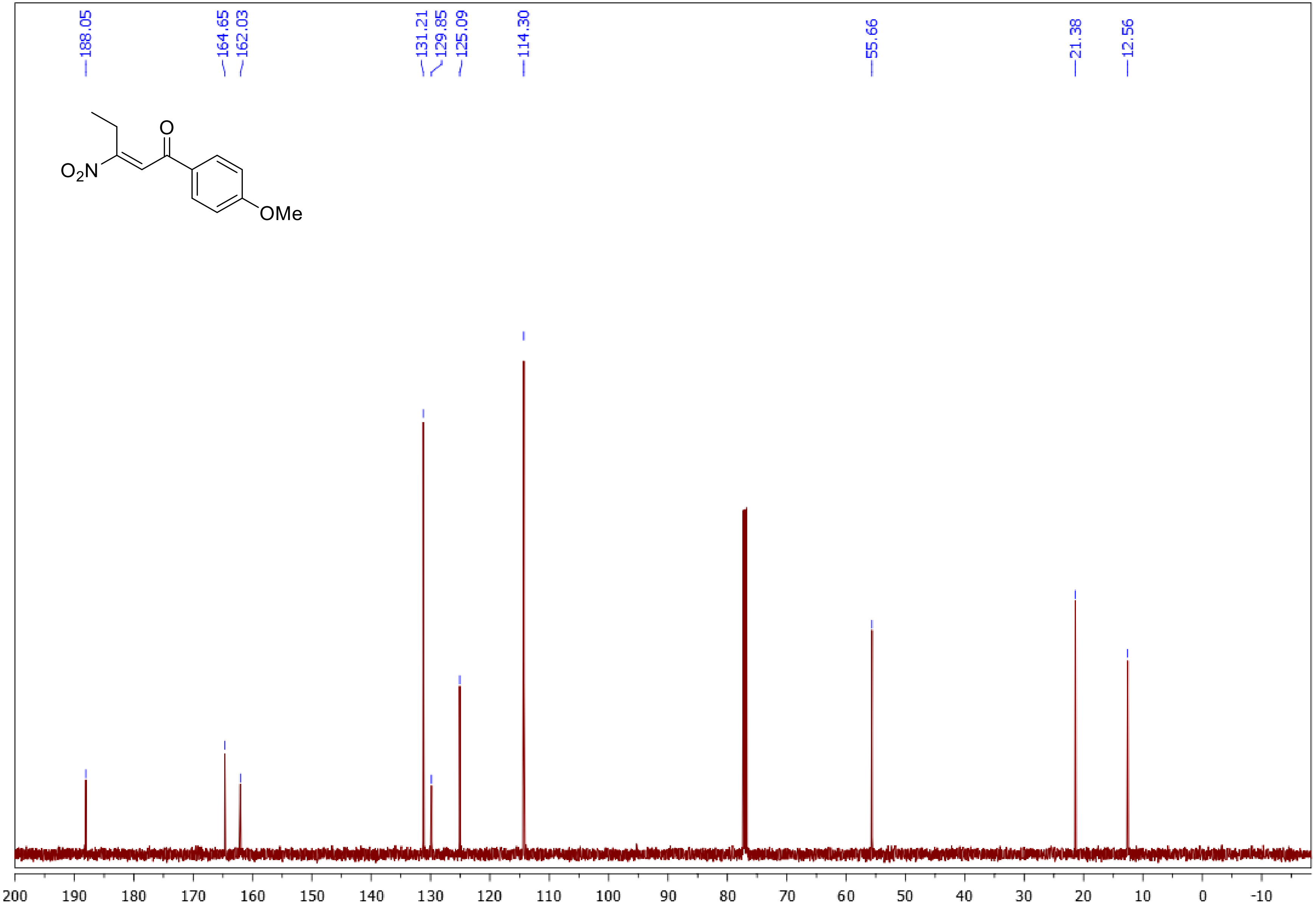

$^{1}$H NMR (400 MHz, $CDCl_3$) **1c**.

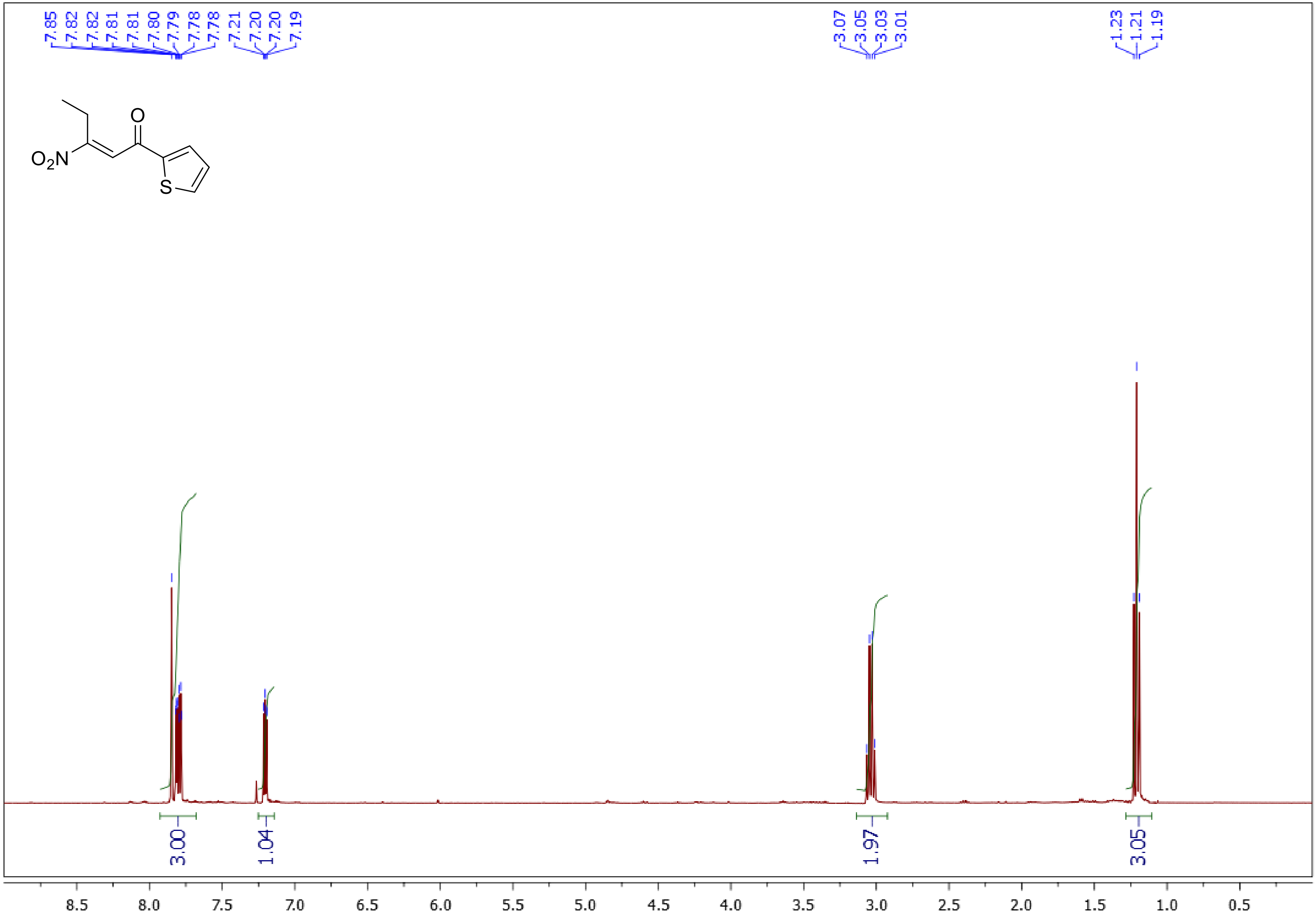

$^{13}C\{^1H\}$ NMR (100 MHz, $CDCl_3$) Compound **1c**.

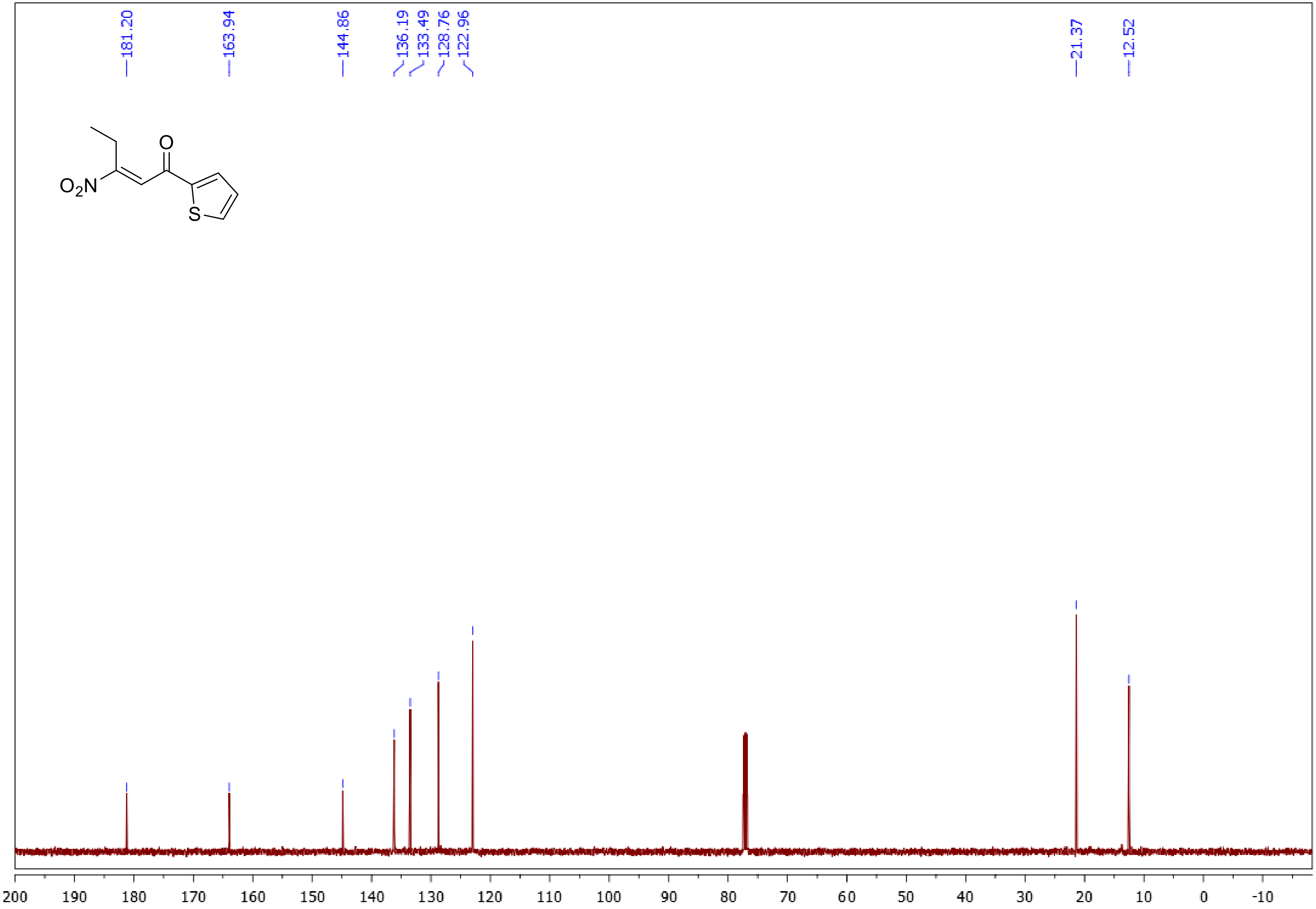

$^{1}$H NMR (400 MHz, $CDCl_3$) Compound **1d**.

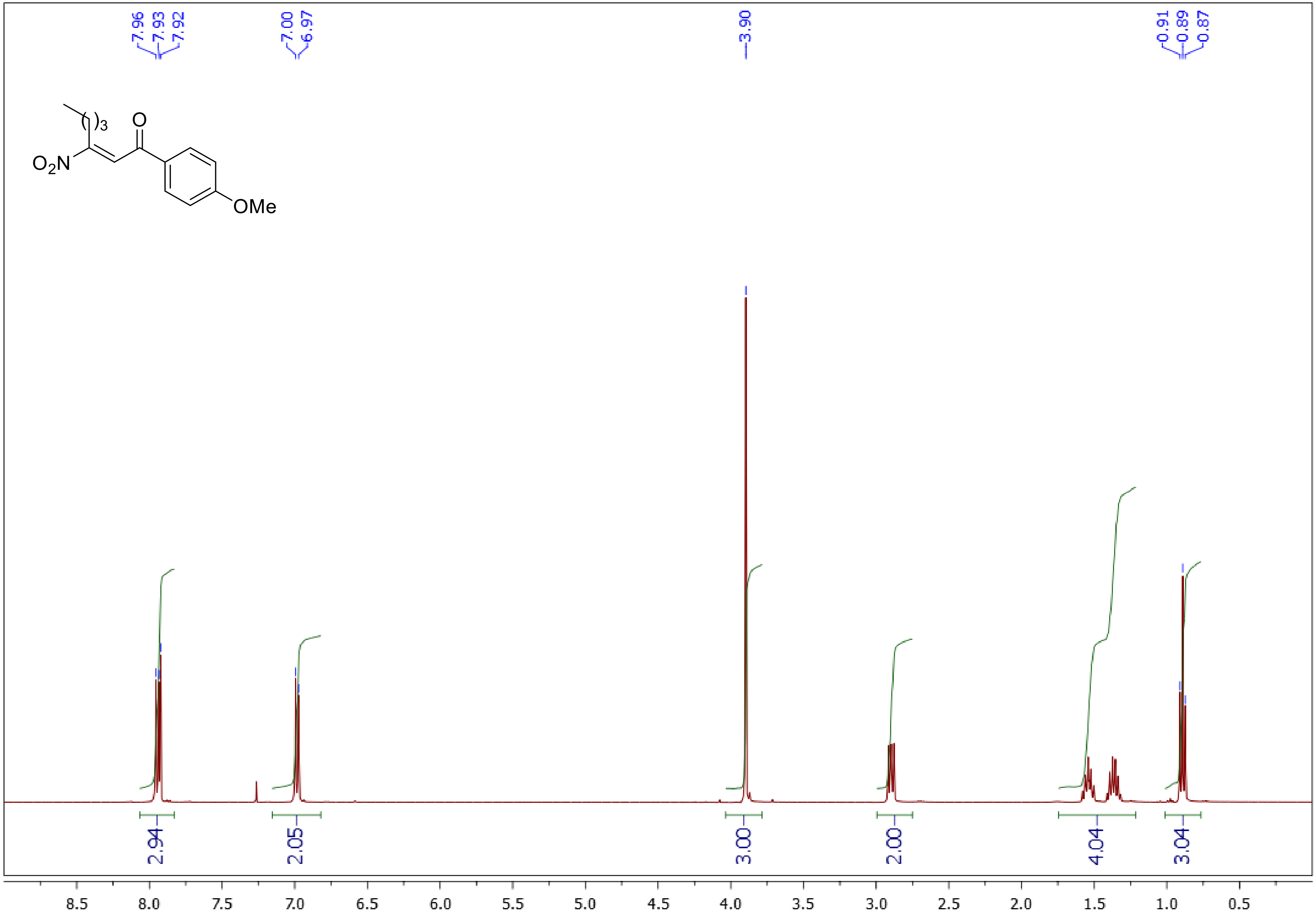

$^{13}C\{^{1}H\}$ NMR (100 MHz, $CDCl_3$) Compound **1d**.

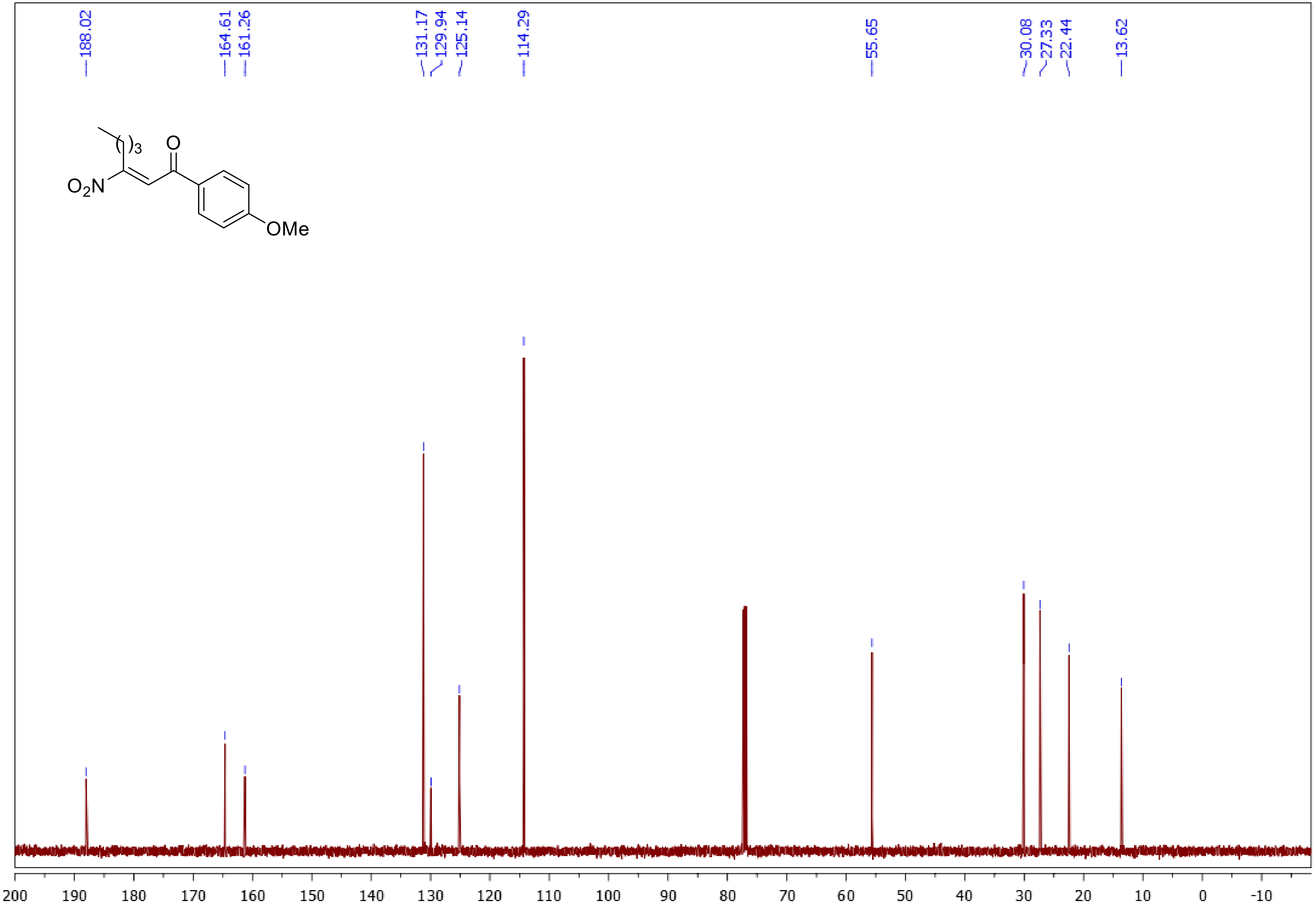

$^{1}$H NMR (400 MHz, $CDCl_3$) Compound **1e**.

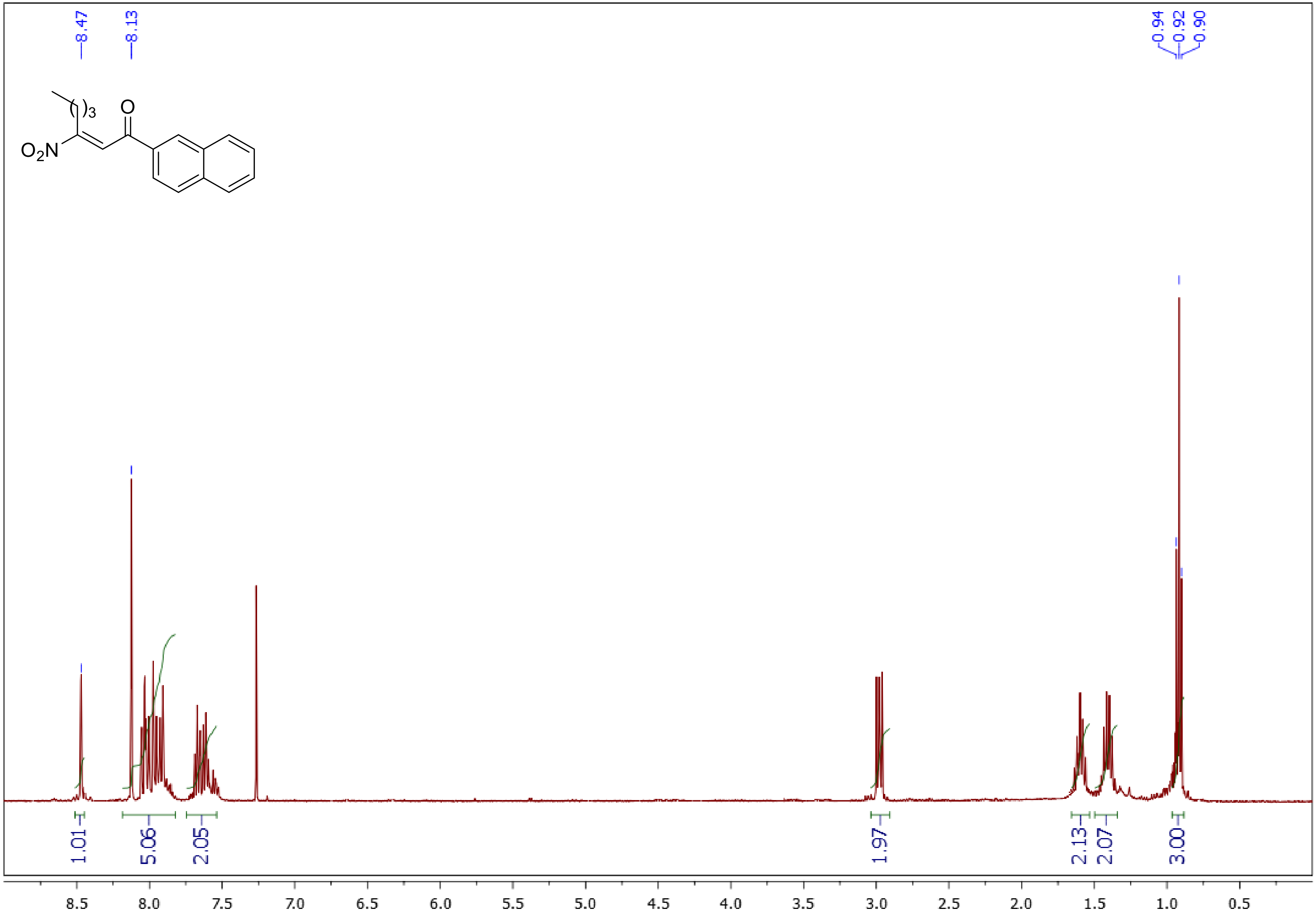

$^{13}C\{^1H\}$ NMR (100 MHz, $CDCl_3$) Compound **1e**.

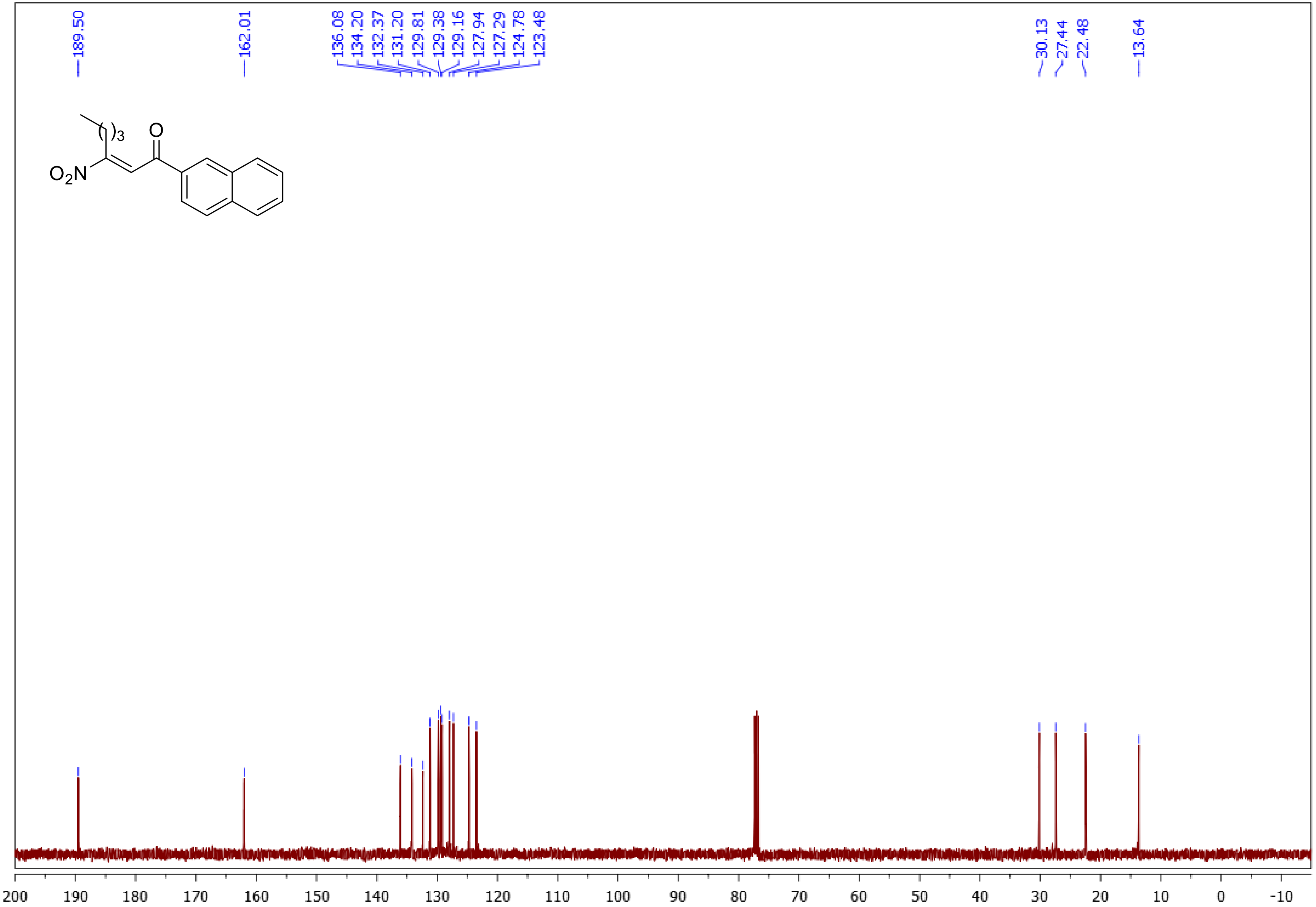

$^{1}$H NMR (400 MHz, $CDCl_3$) Compound **1f**.

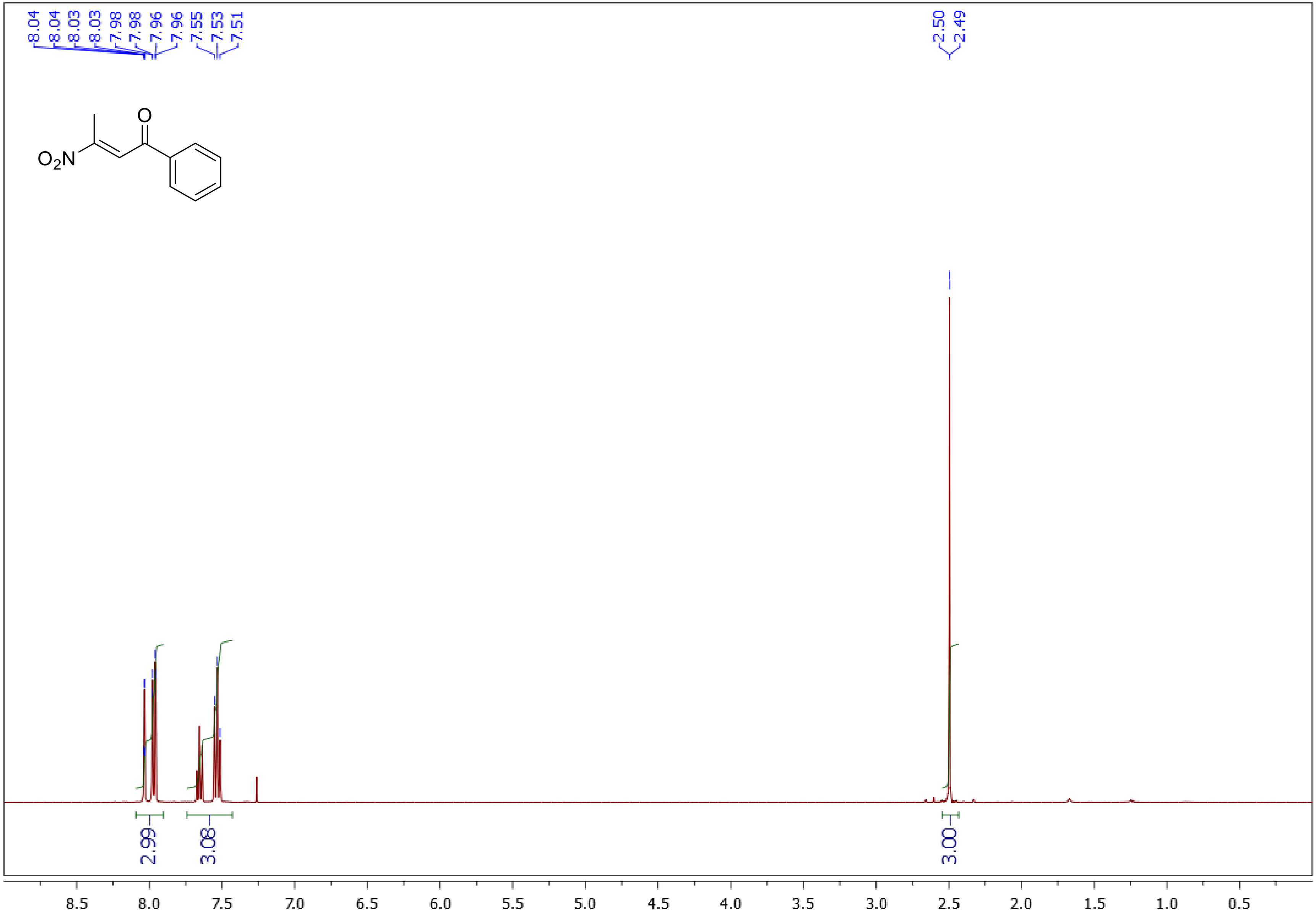

$^{13}C\{^1H\}$ NMR (100 MHz, $CDCl_3$) Compound **1f**.

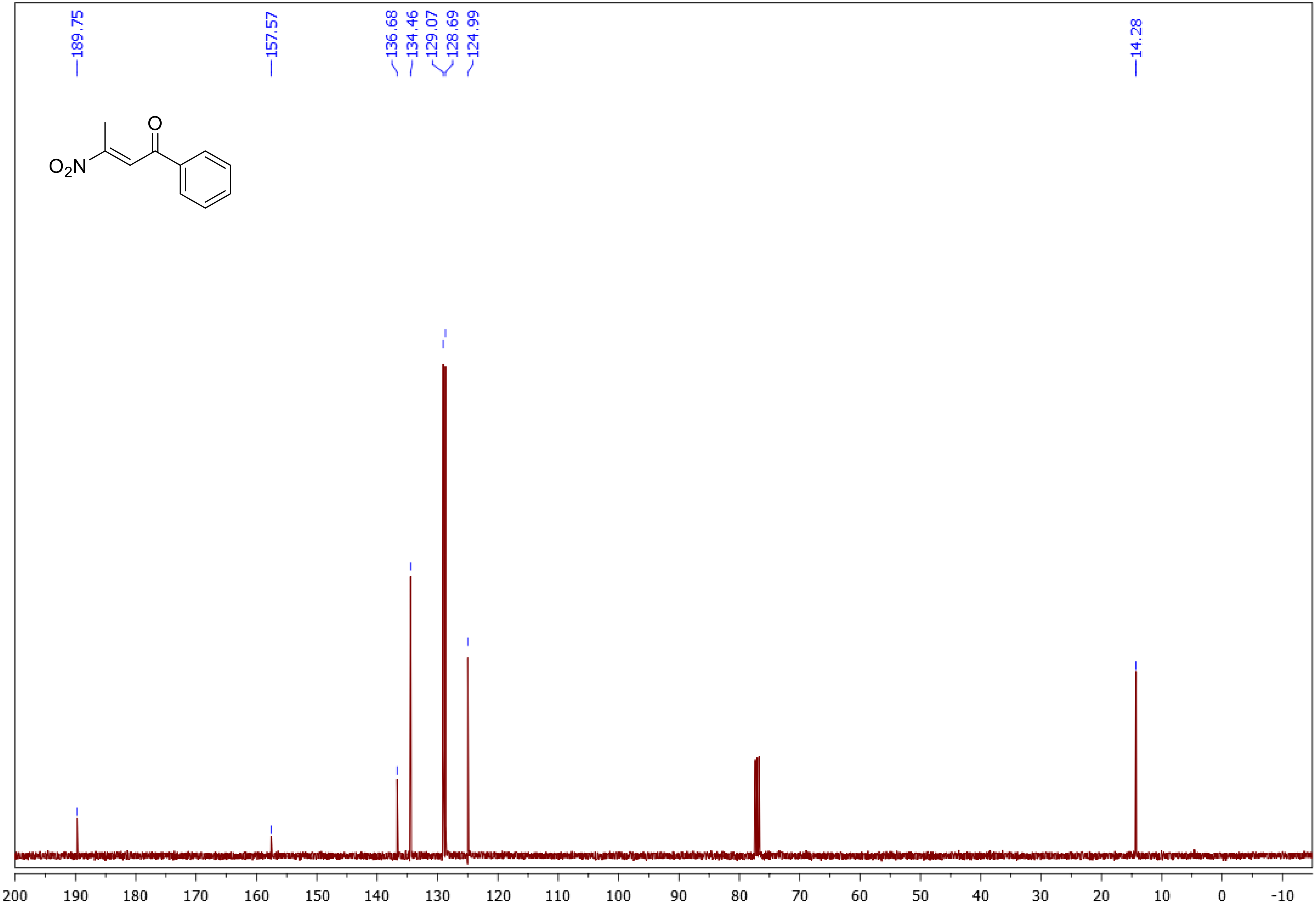

$^1$H NMR (400 MHz, $CDCl_3$) Compound **1g**.

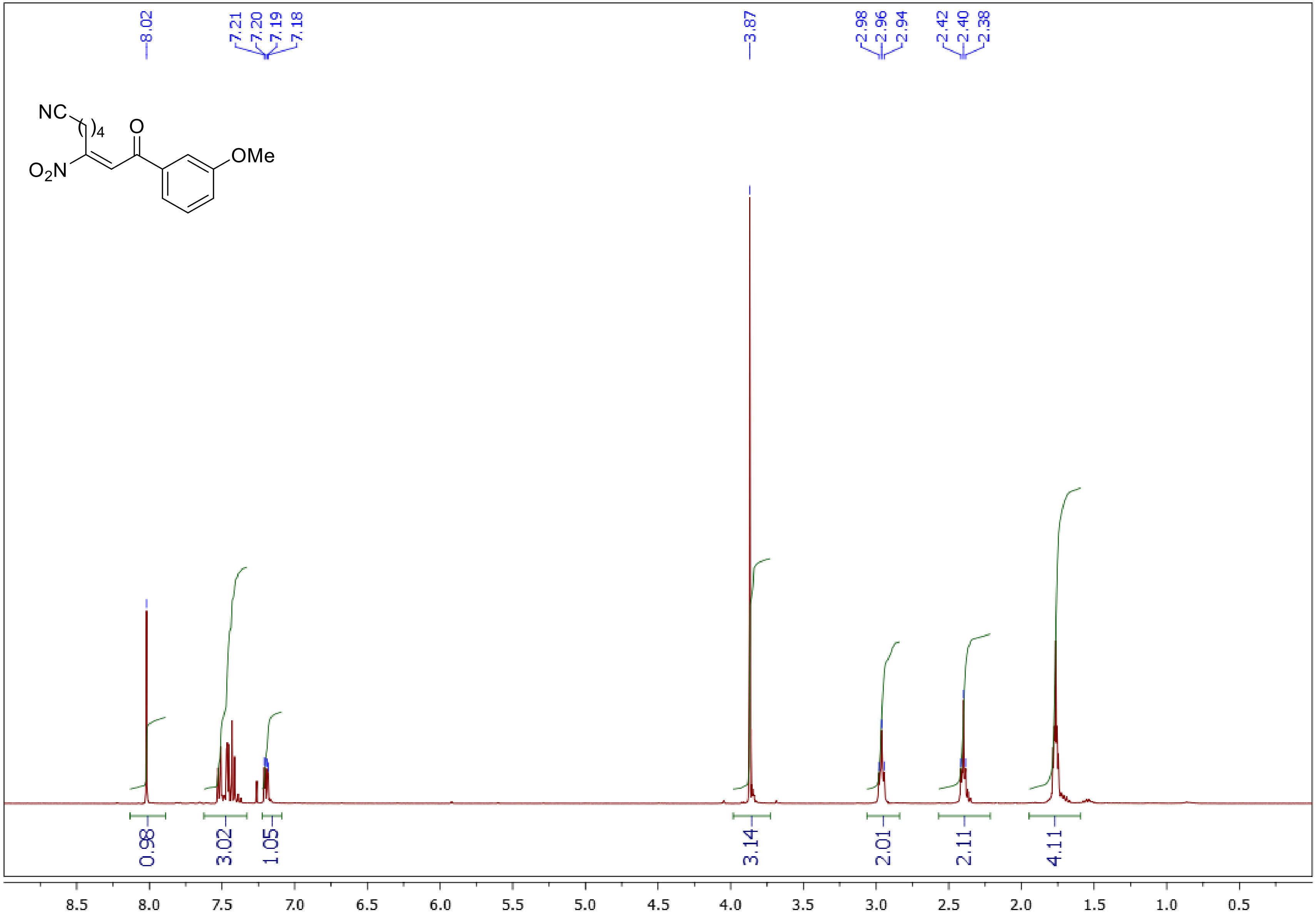

$^{13}C\{^{1}H\}$ NMR (100 MHz, $CDCl_3$) Compound **1g**.

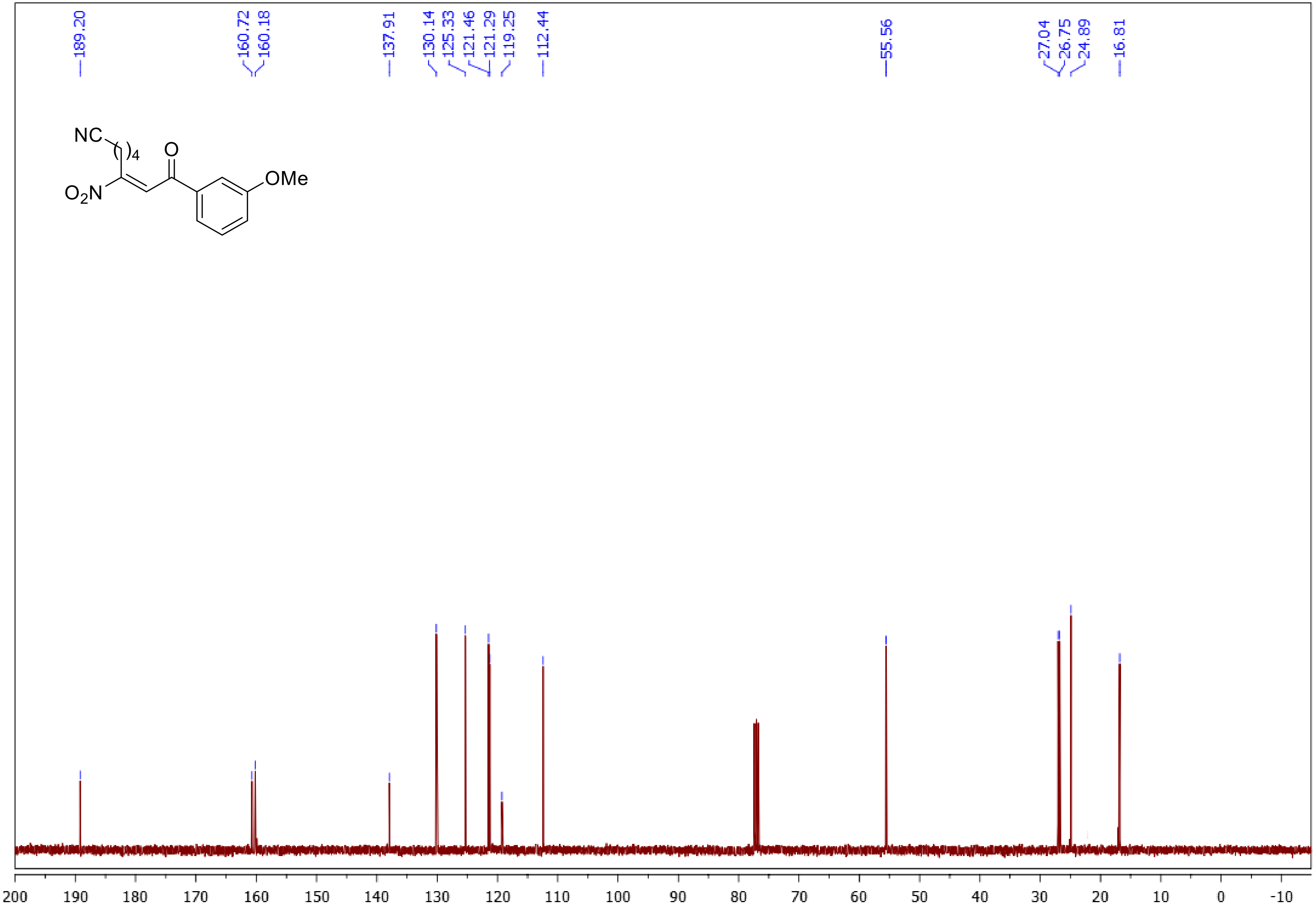

$^{1}$H NMR (400 MHz, $CDCl_3$) Compound **1h**.

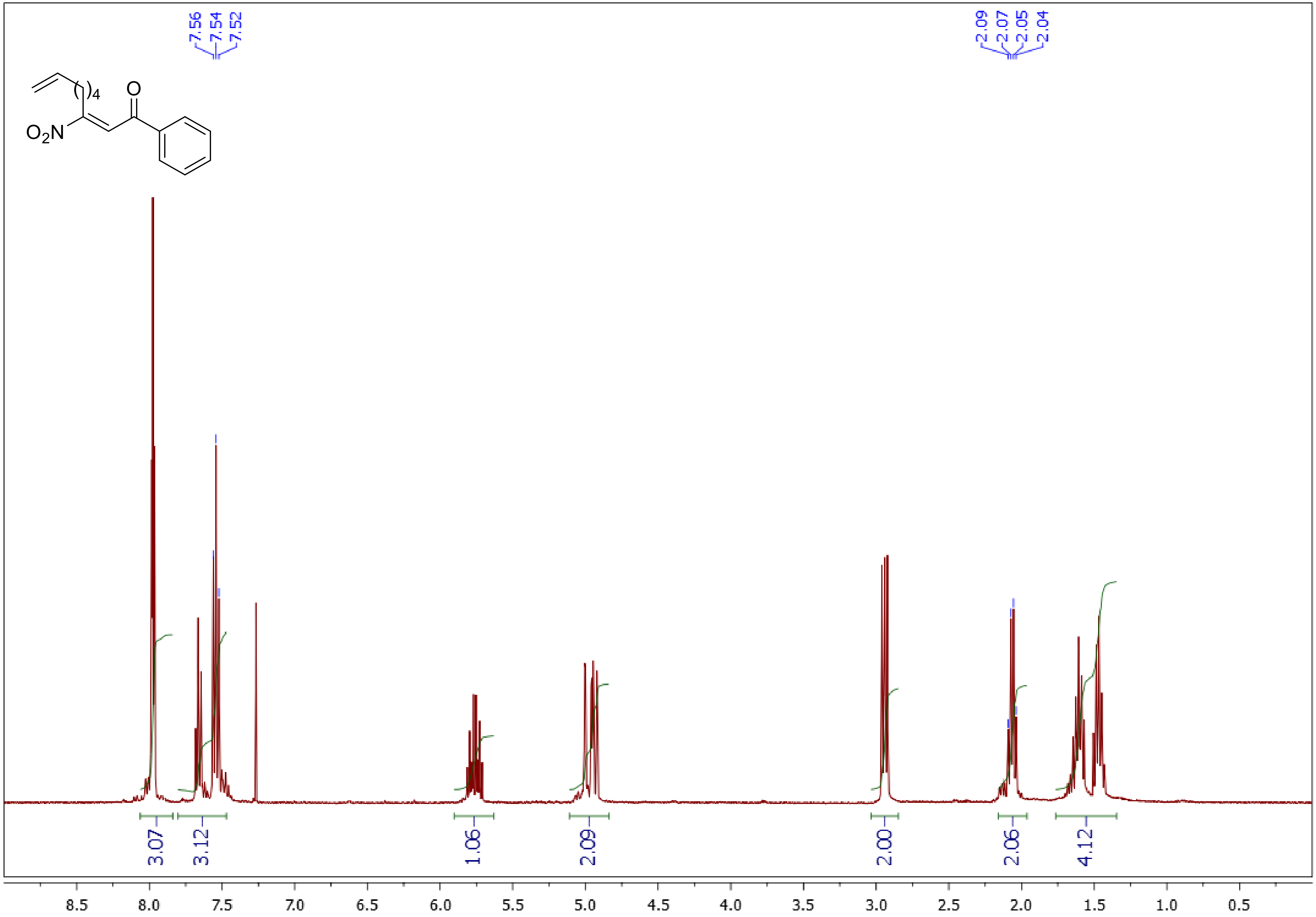

$^{13}C\{^1H\}$ NMR (100 MHz, $CDCl_3$) Compound **1h**.

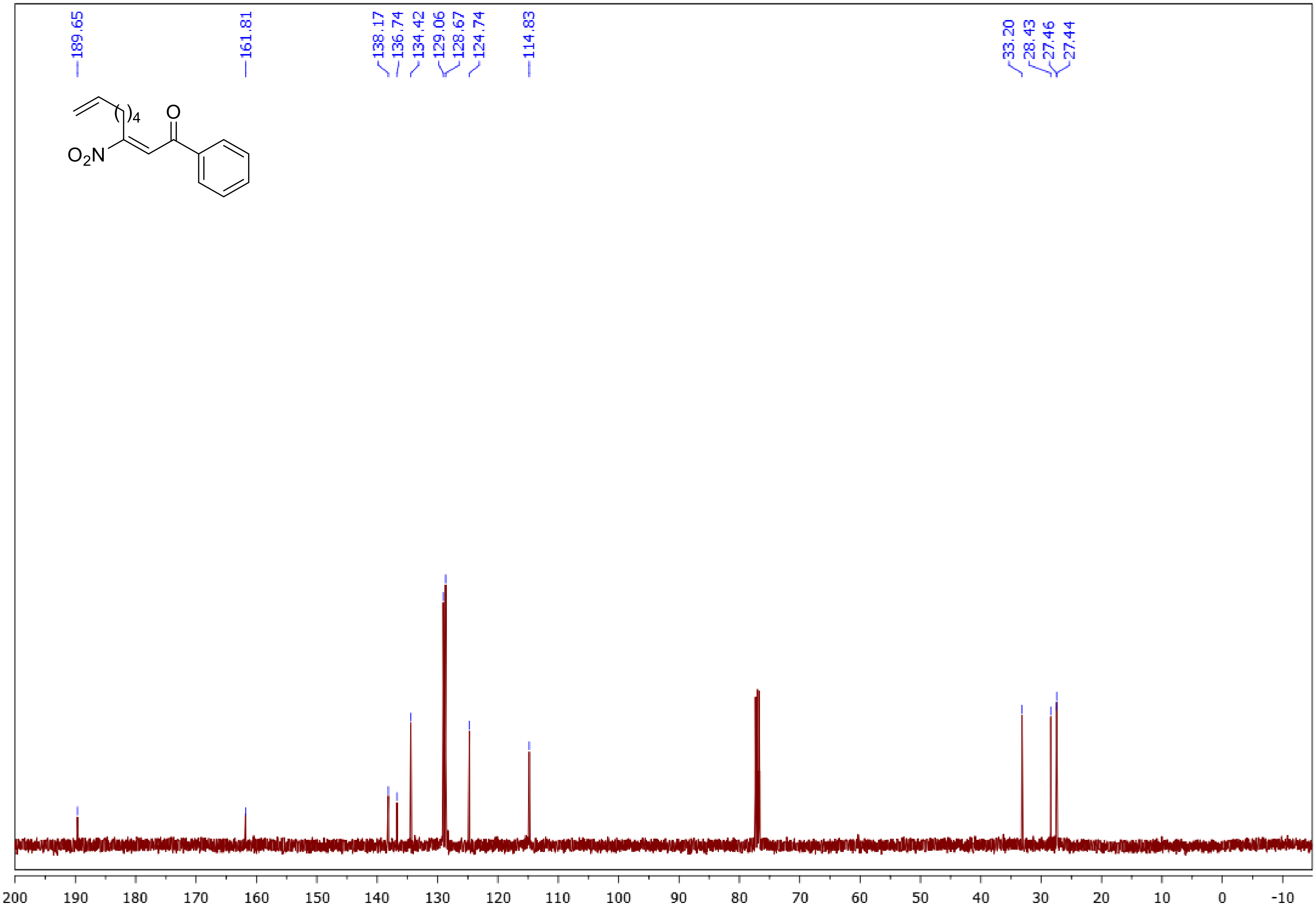

$^{1}H$ NMR (400 MHz, $CDCl_3$) Compound **1i**.

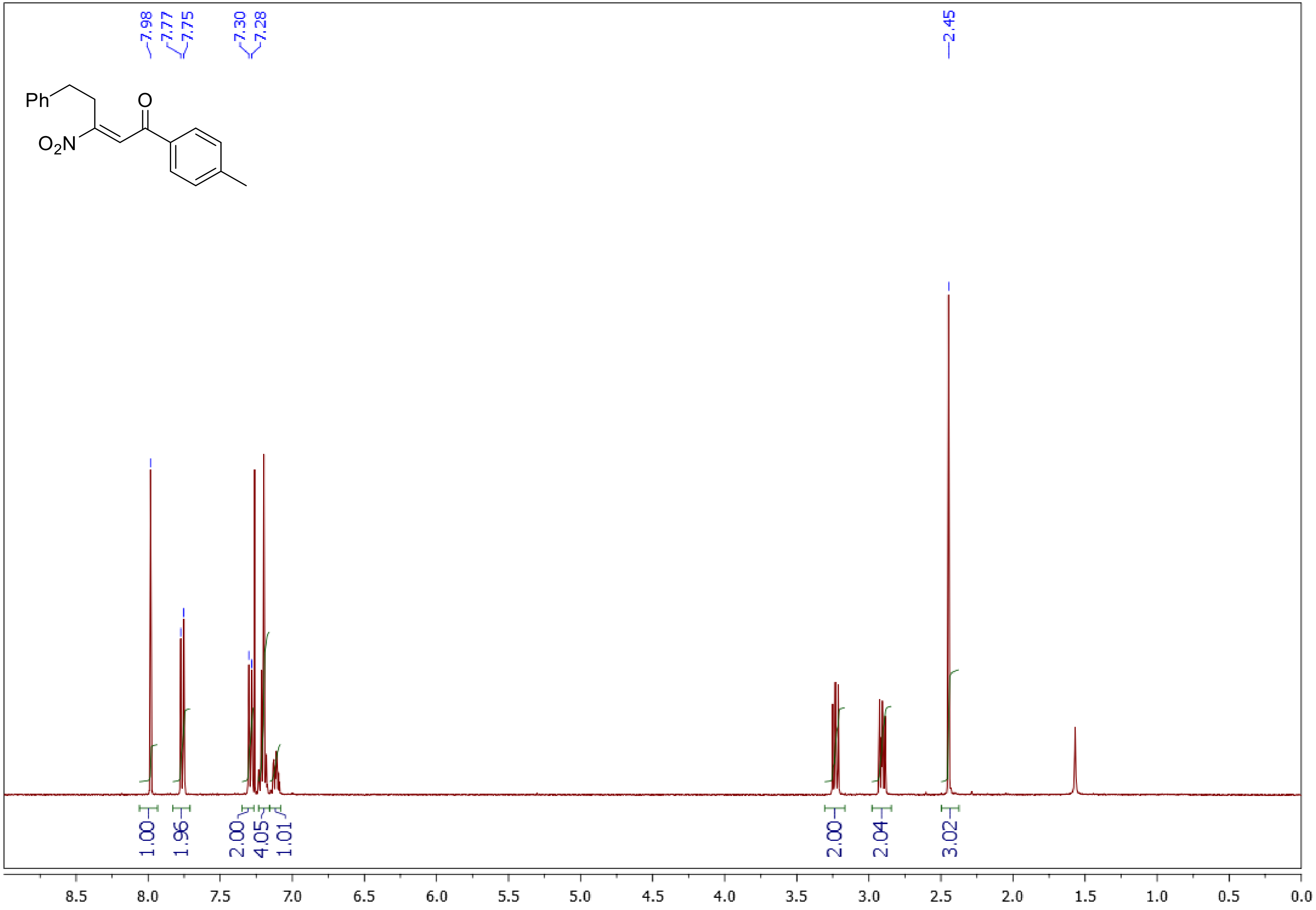

$^{13}C\{^1H\}$ NMR (100 MHz, $CDCl_3$) Compound **1i**.

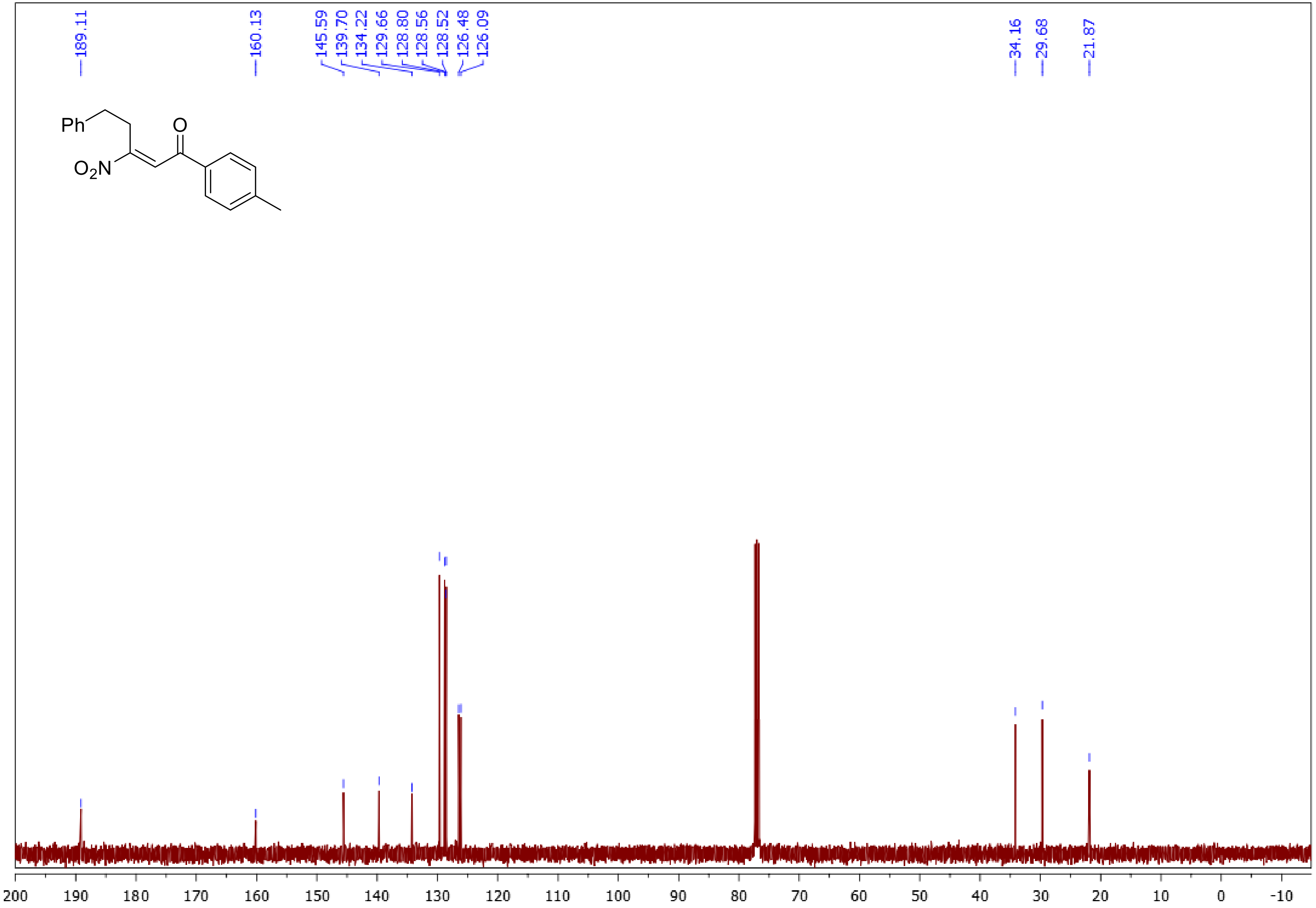

$^{1}$H NMR (400 MHz, $CDCl_3$) Compound **1j**.

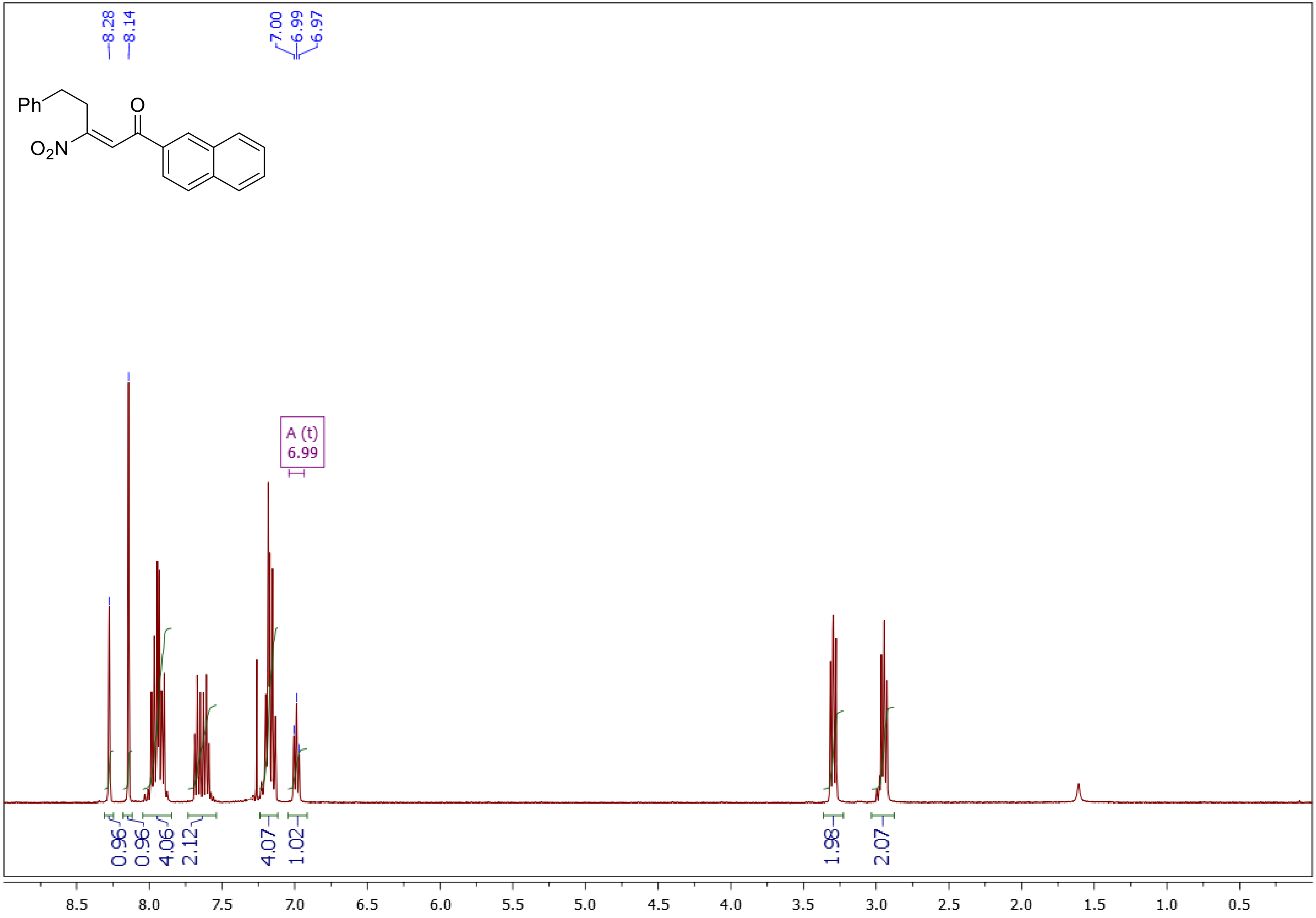

$^{13}C\{^{1}H\}$ NMR (100 MHz, $CDCl_3$) Compound **1j**.

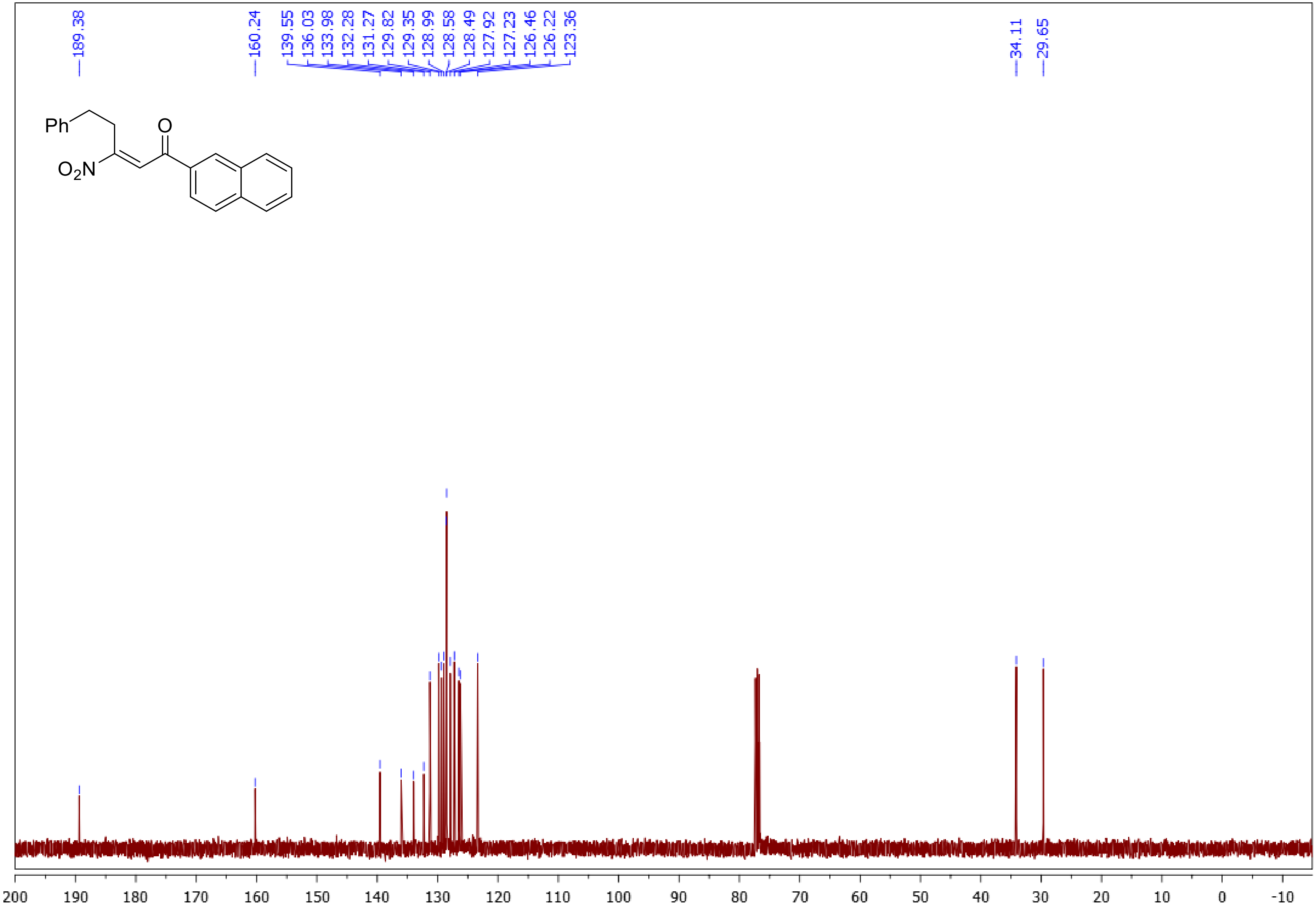

$^{1}$H NMR (400 MHz, $CDCl_3$) Compound **1k**.

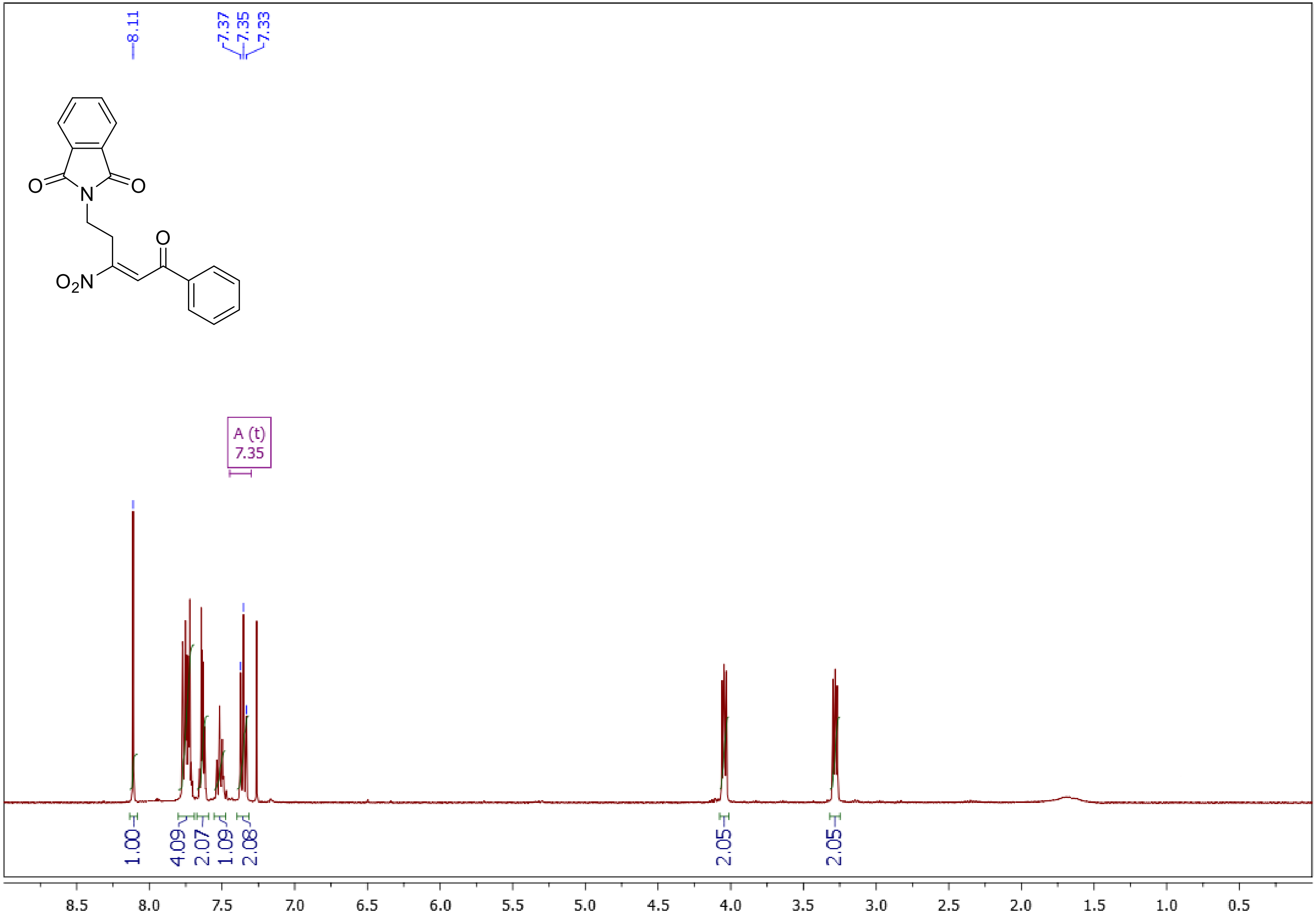

$^{13}C\{^1H\}$ NMR (100 MHz, $CDCl_3$) Compound **1k**.

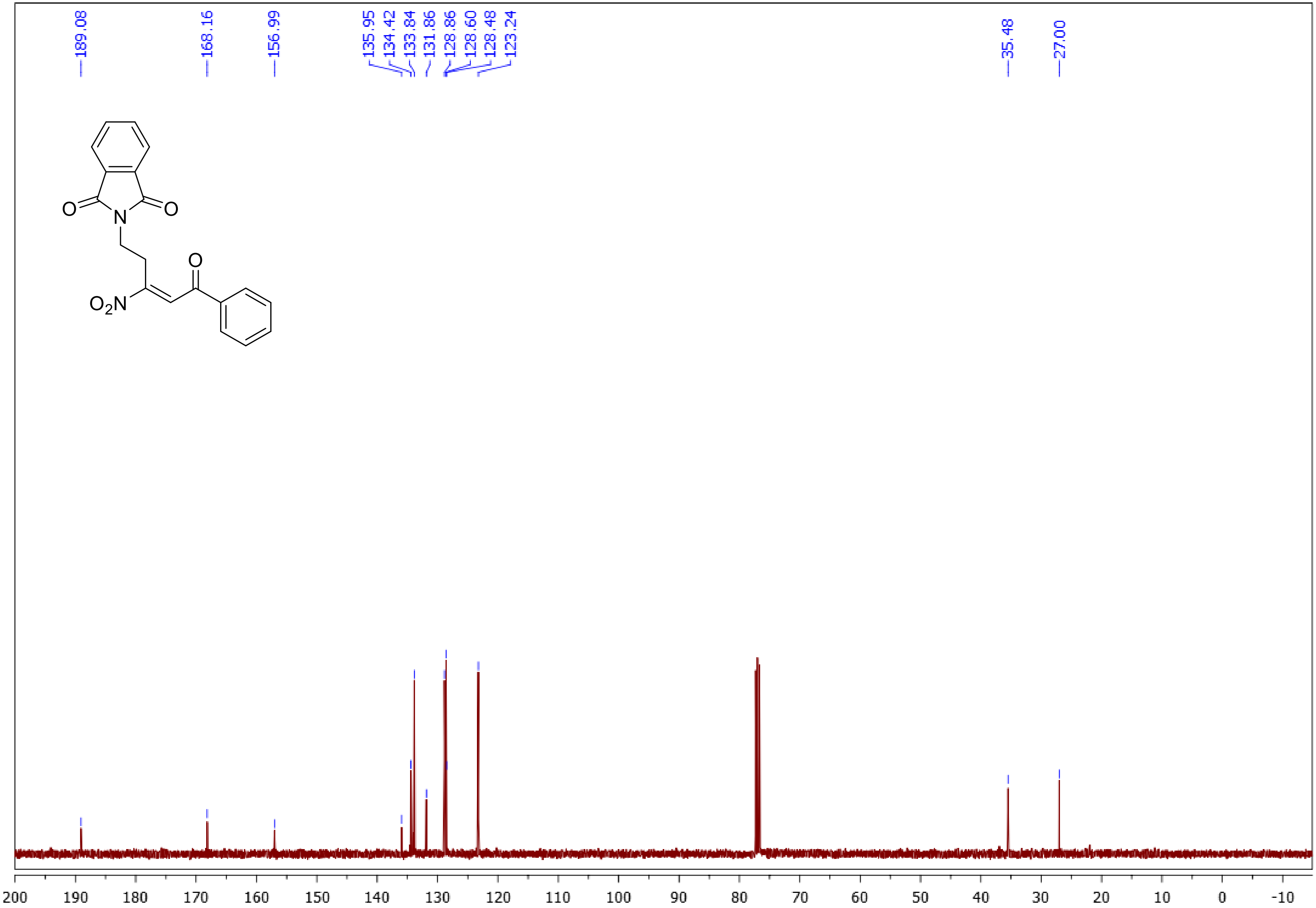

$^{1}$H NMR (400 MHz, $CDCl_3$) Compound **1l**.

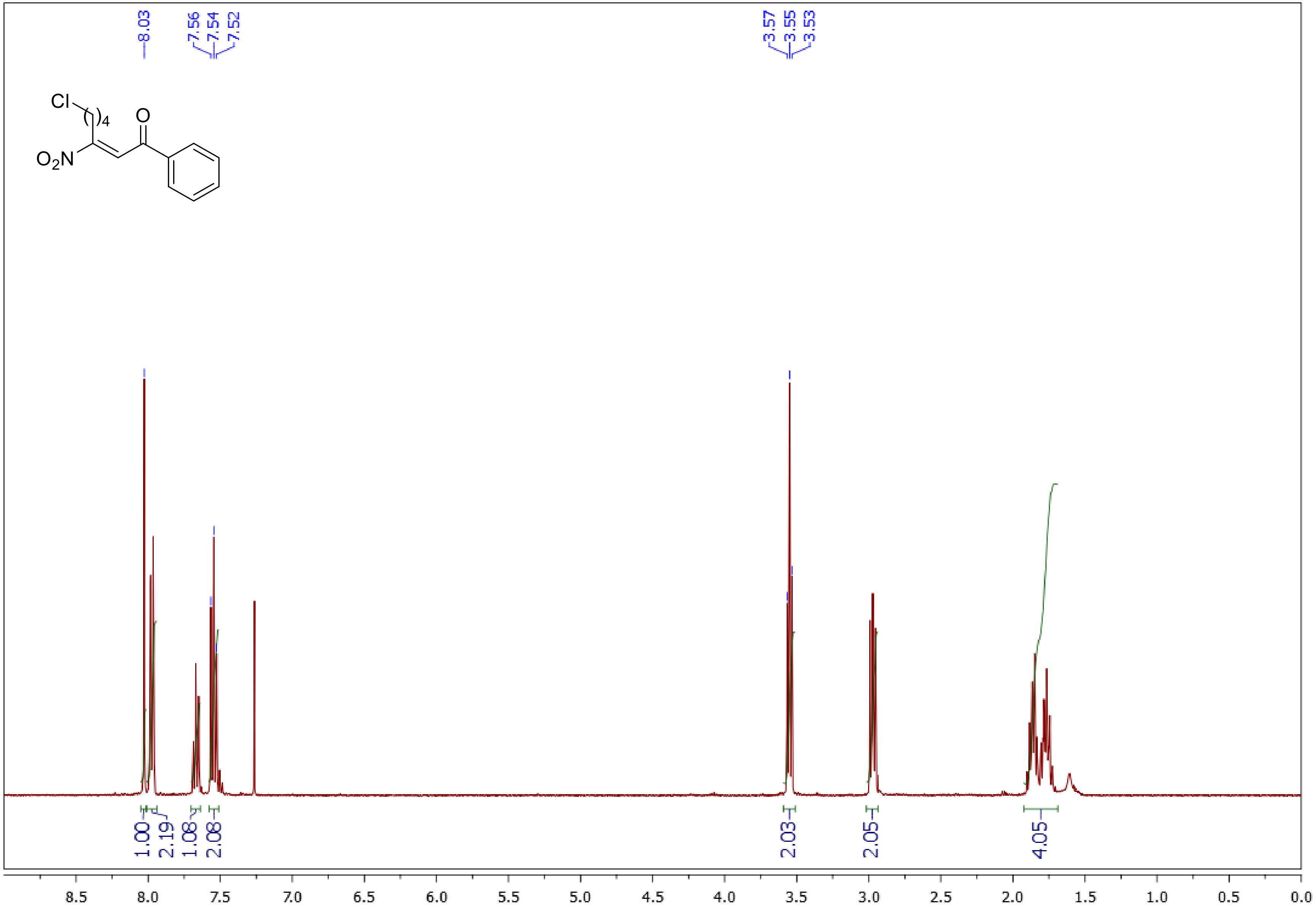

$^{13}C\{^1H\}$ NMR (100 MHz, $CDCl_3$) Compound **1l**.

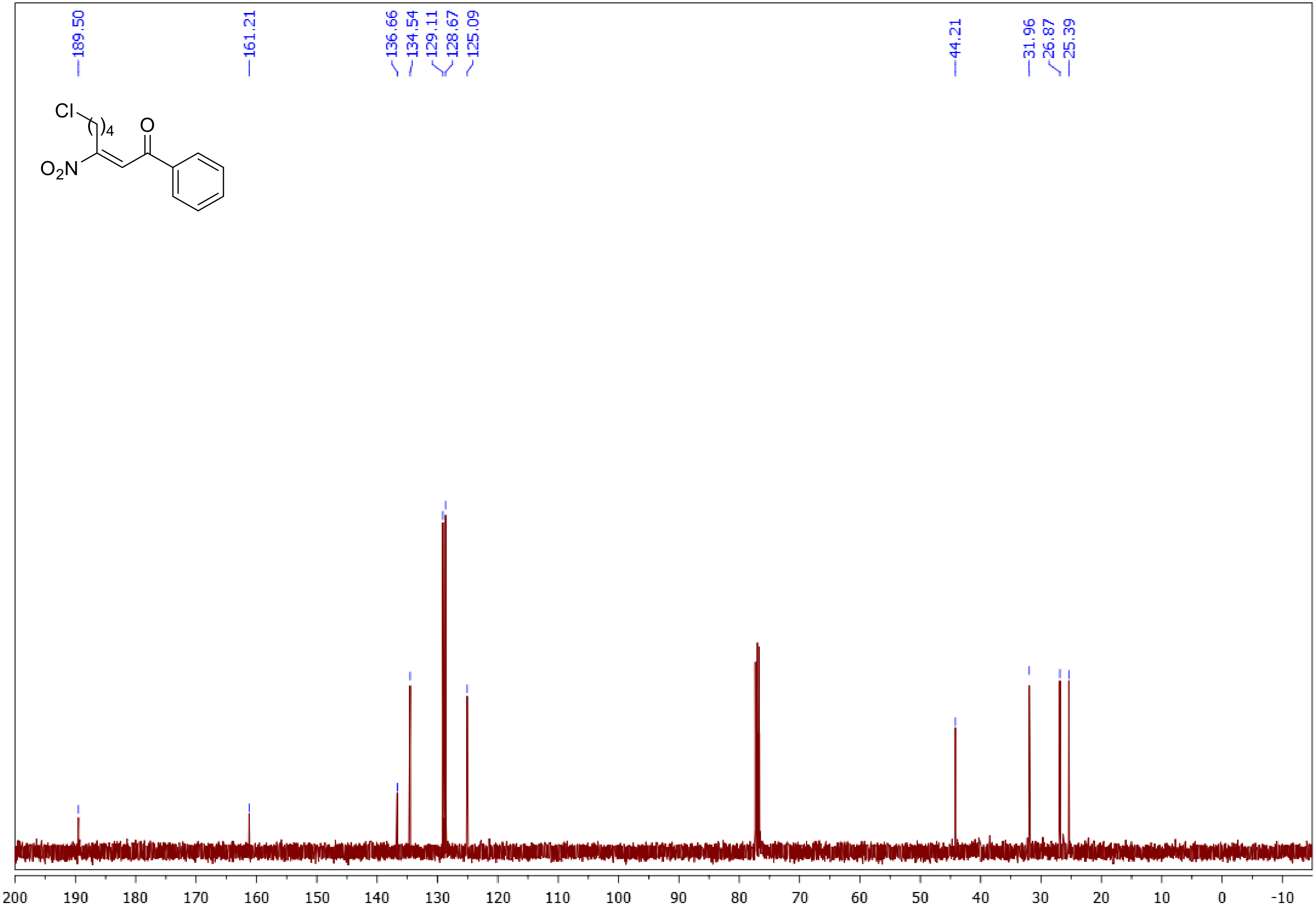

$^{1}H$ NMR (400 MHz, $CDCl_3$) Compound **1m**.

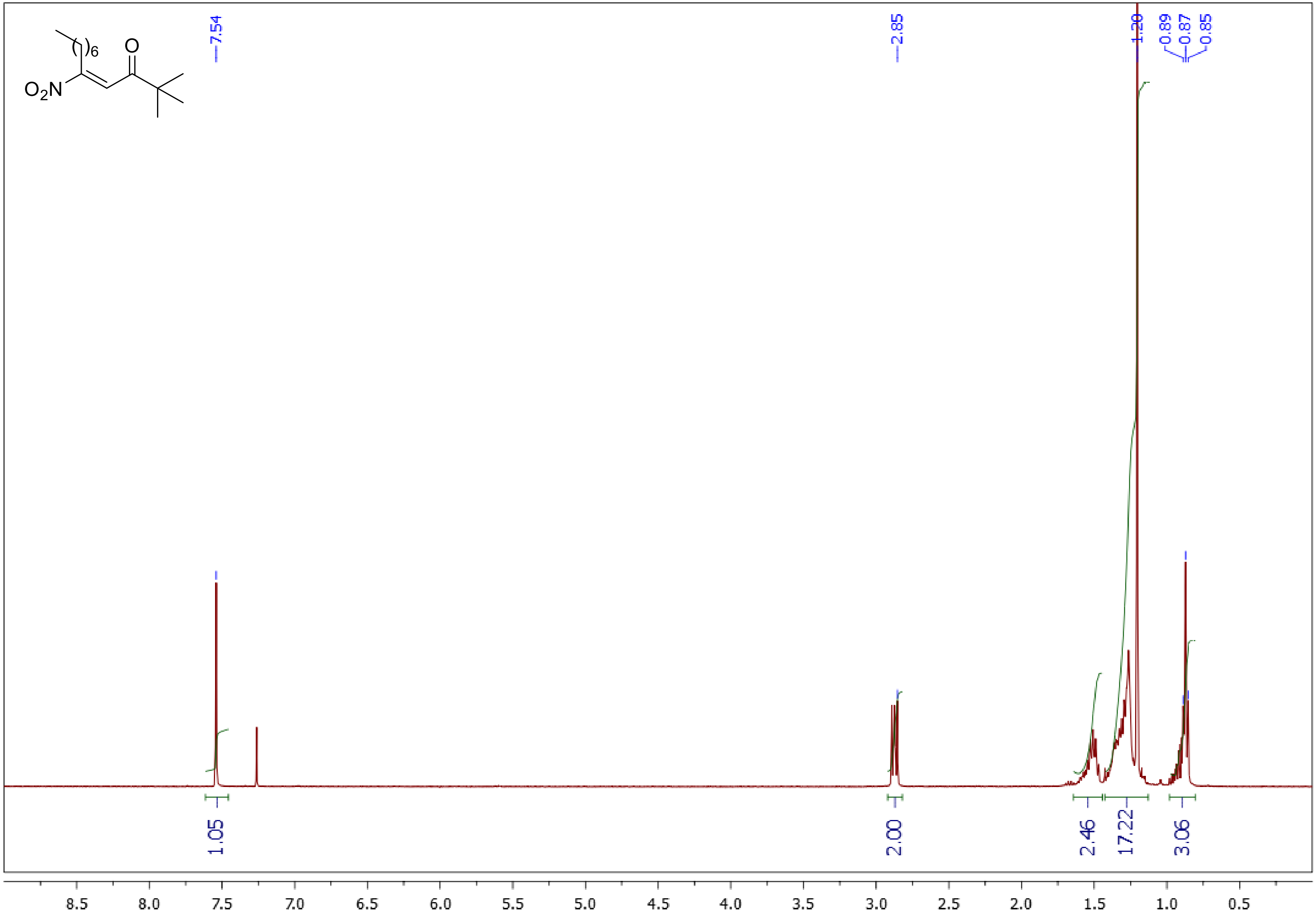

$^{13}C\{^1H\}$ NMR (100 MHz, $CDCl_3$) Compound **1m**.

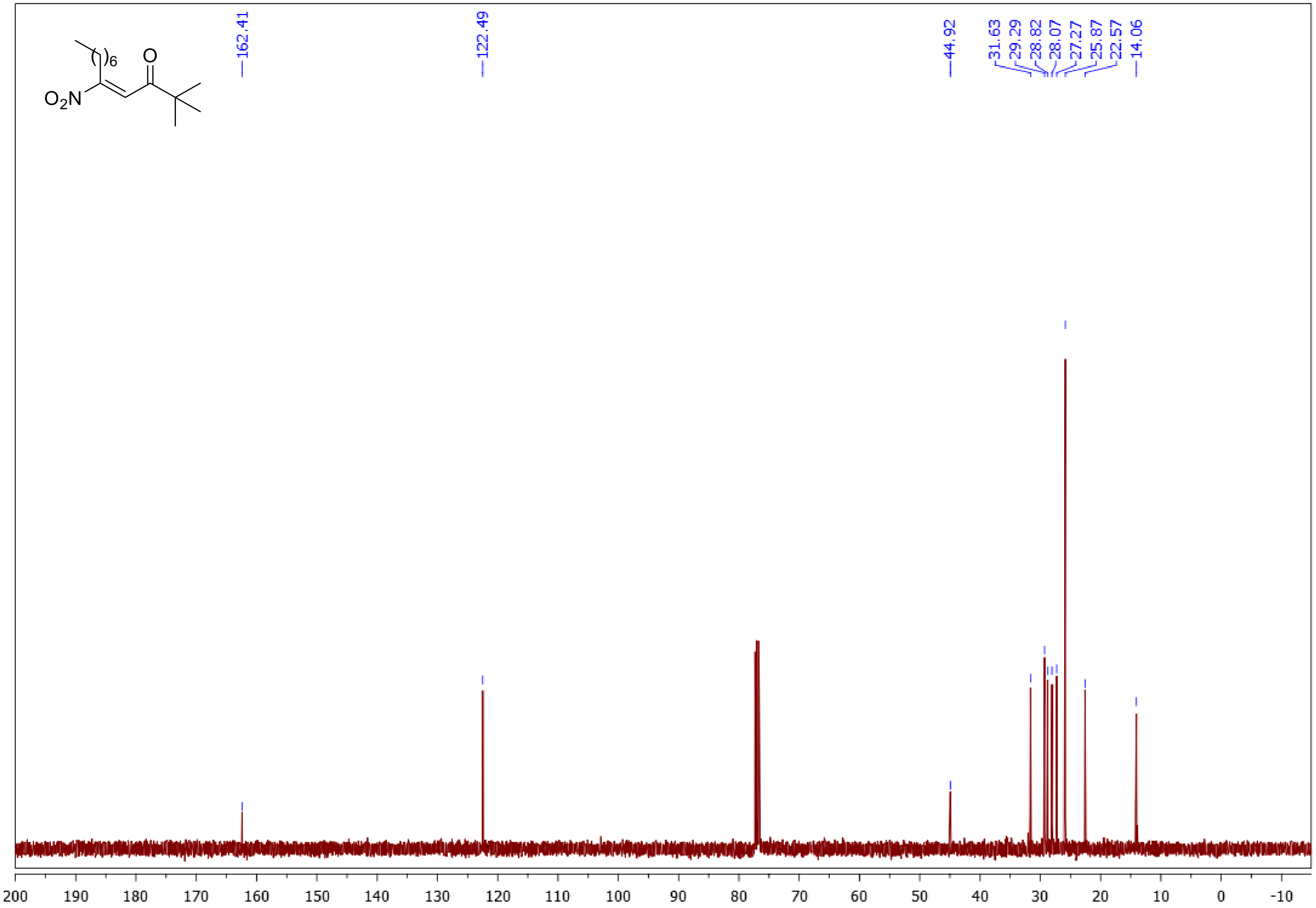

$^1$H NMR (400 MHz, $CDCl_3$) Compound **1n**.

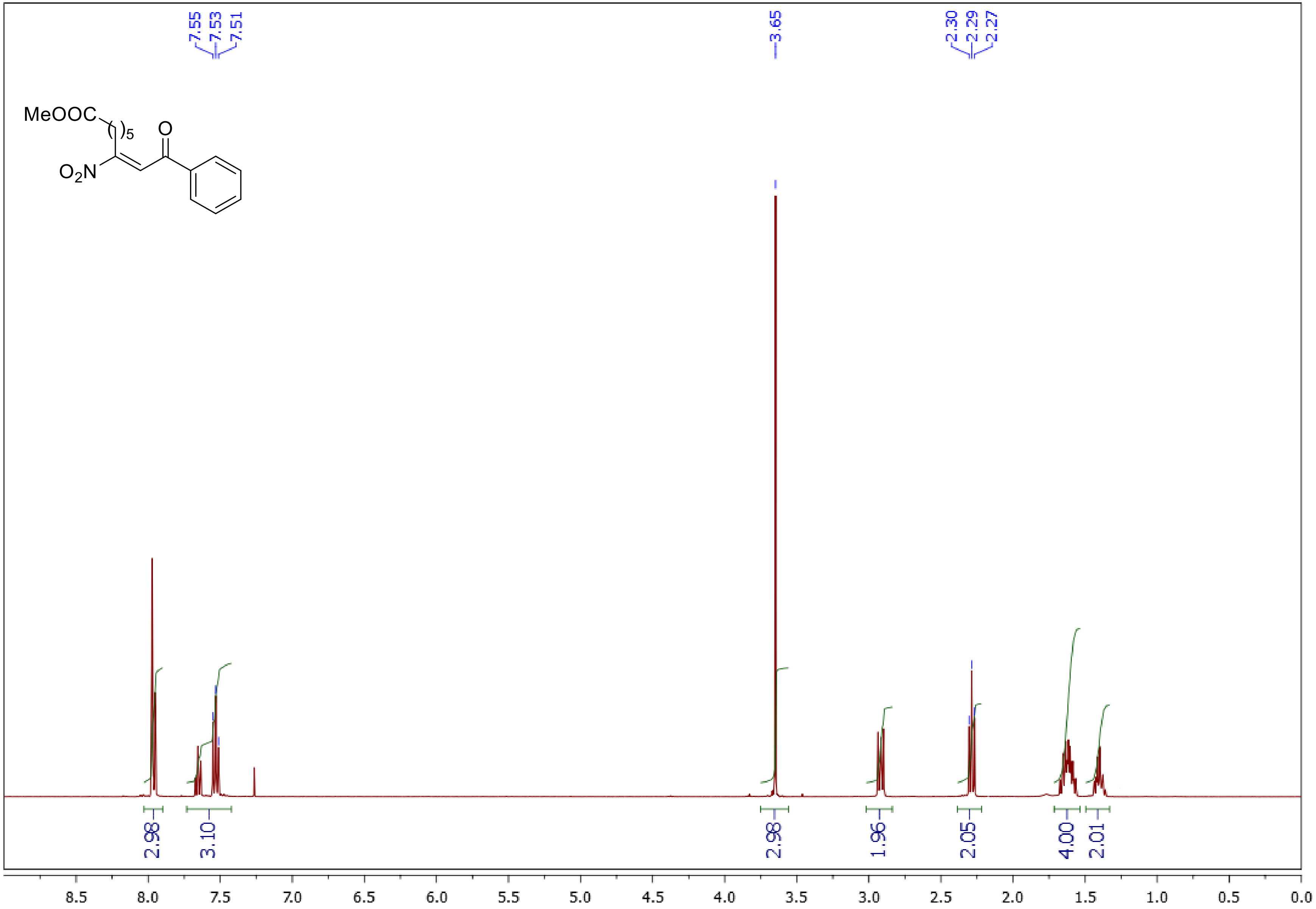

$^{13}C\{^1H\}$ NMR (100 MHz, $CDCl_3$) Compound **1n**.

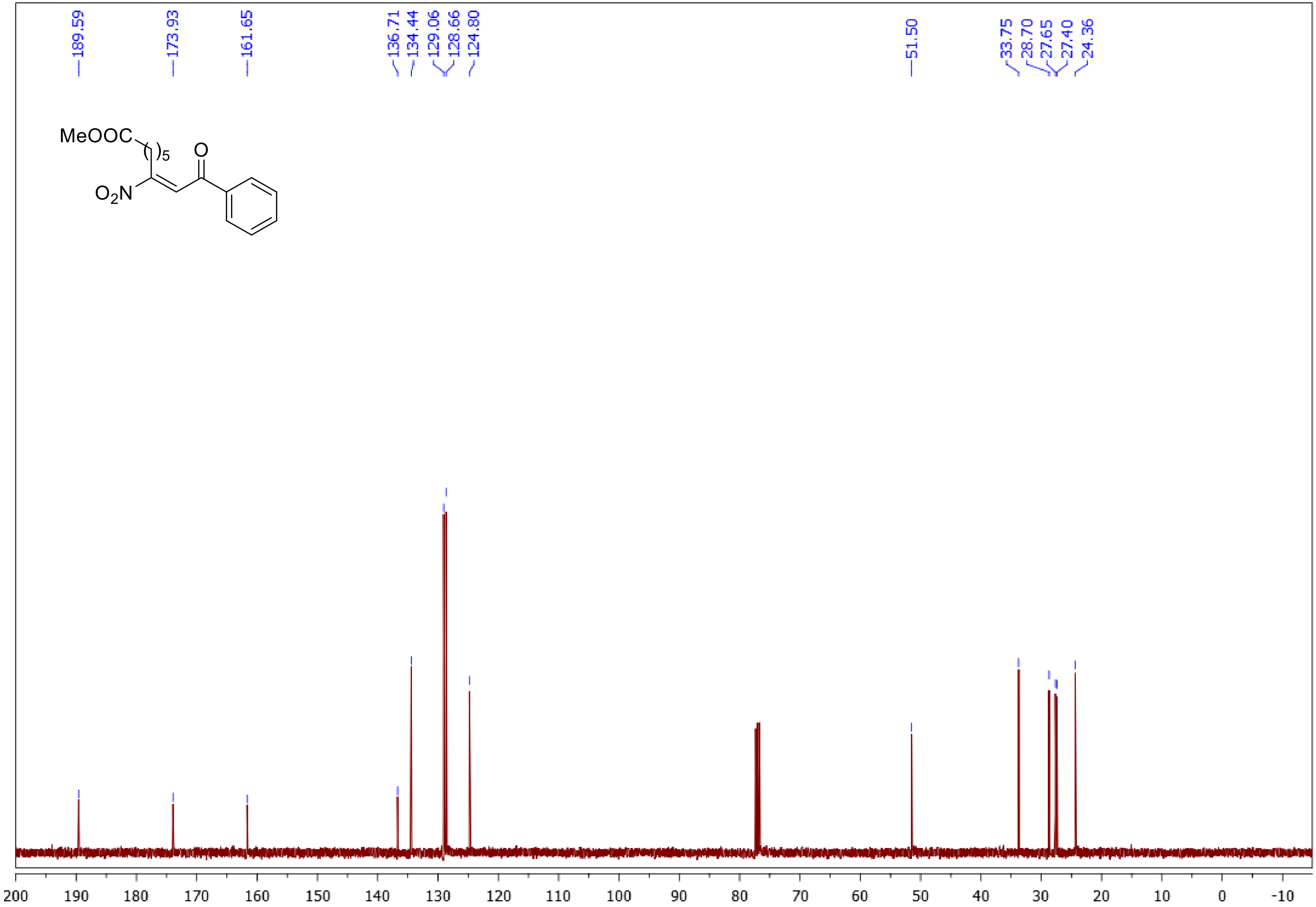

$^{1}H$ NMR (400 MHz, $CDCl_3$) Compound **1o**.

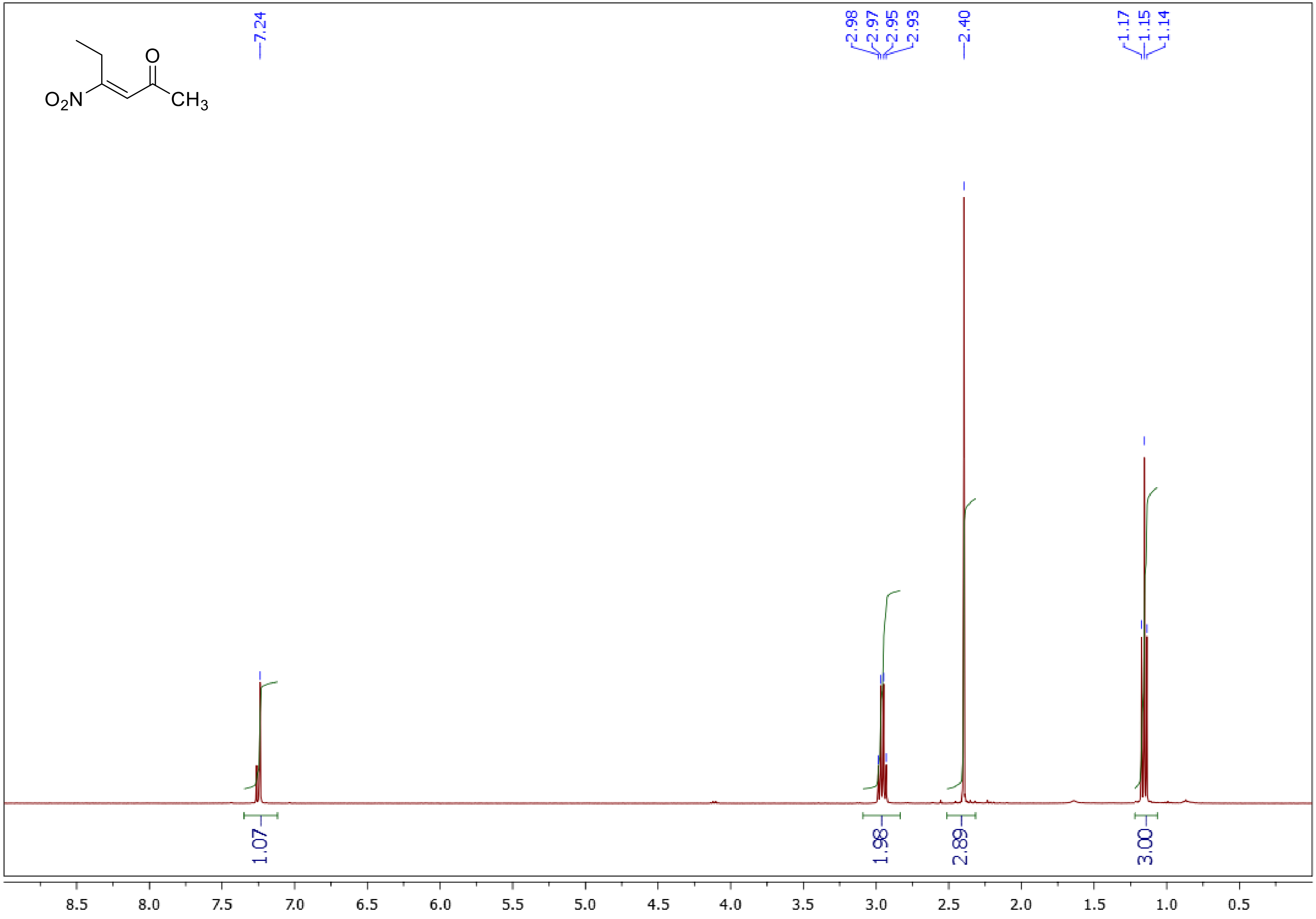

$^{13}C\{^{1}H\}$ NMR (100 MHz, $CDCl_3$) Compound **1o**.

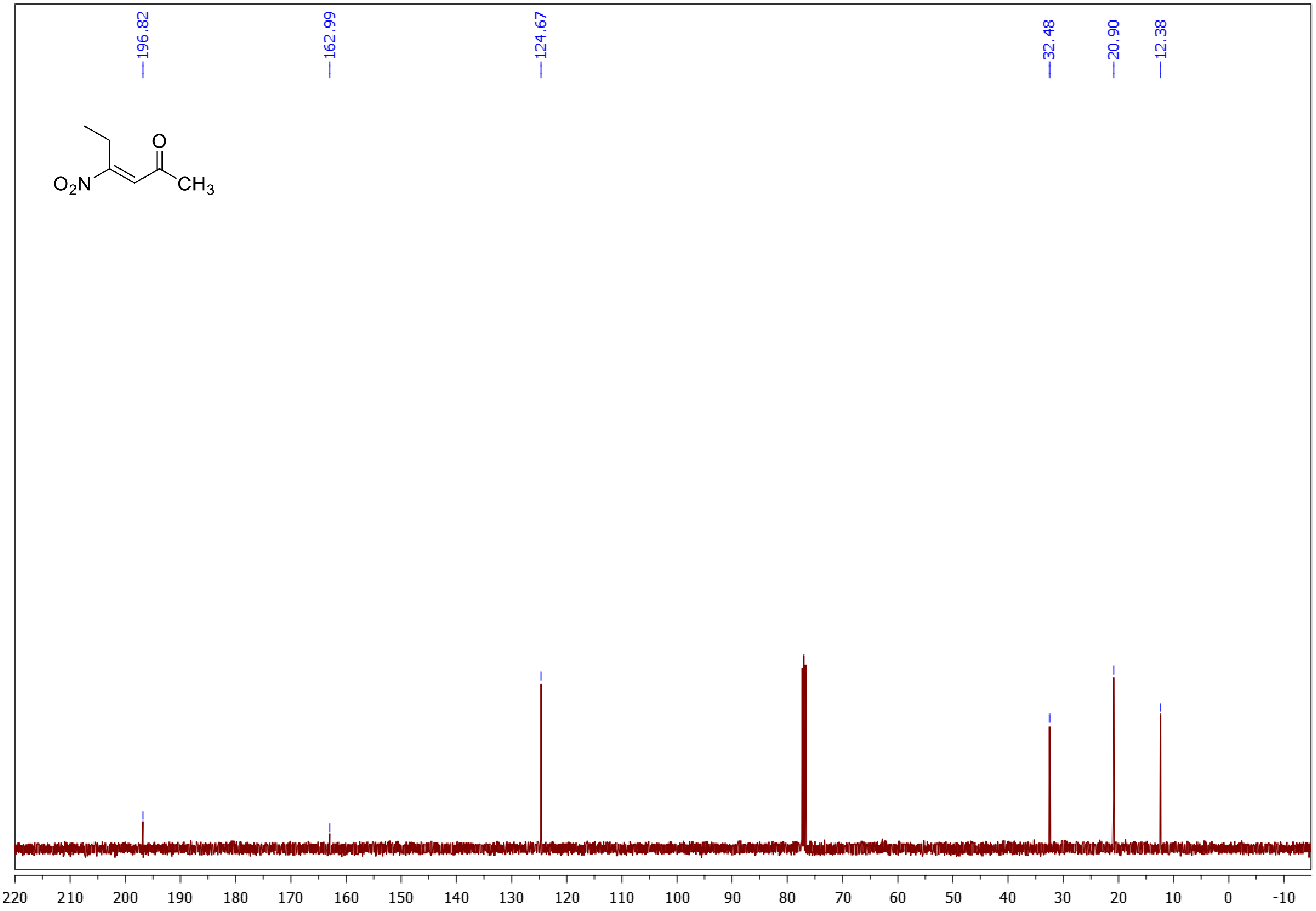

$^1$H NMR (400 MHz, $CDCl_3$) Compound **3a**.

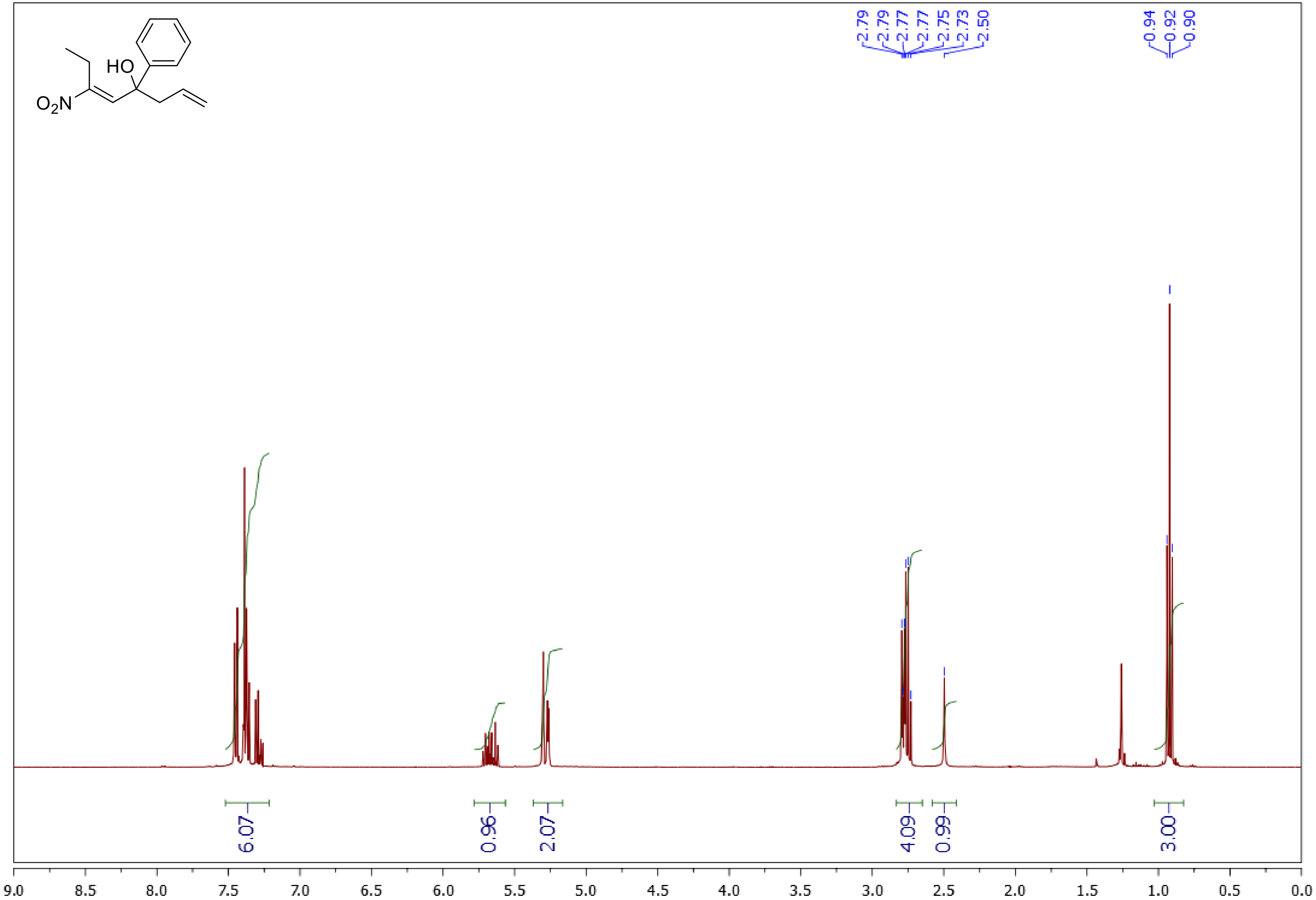

$^{13}C\{^{1}H\}$ NMR (100 MHz, $CDCl_3$) Compound **3a**.

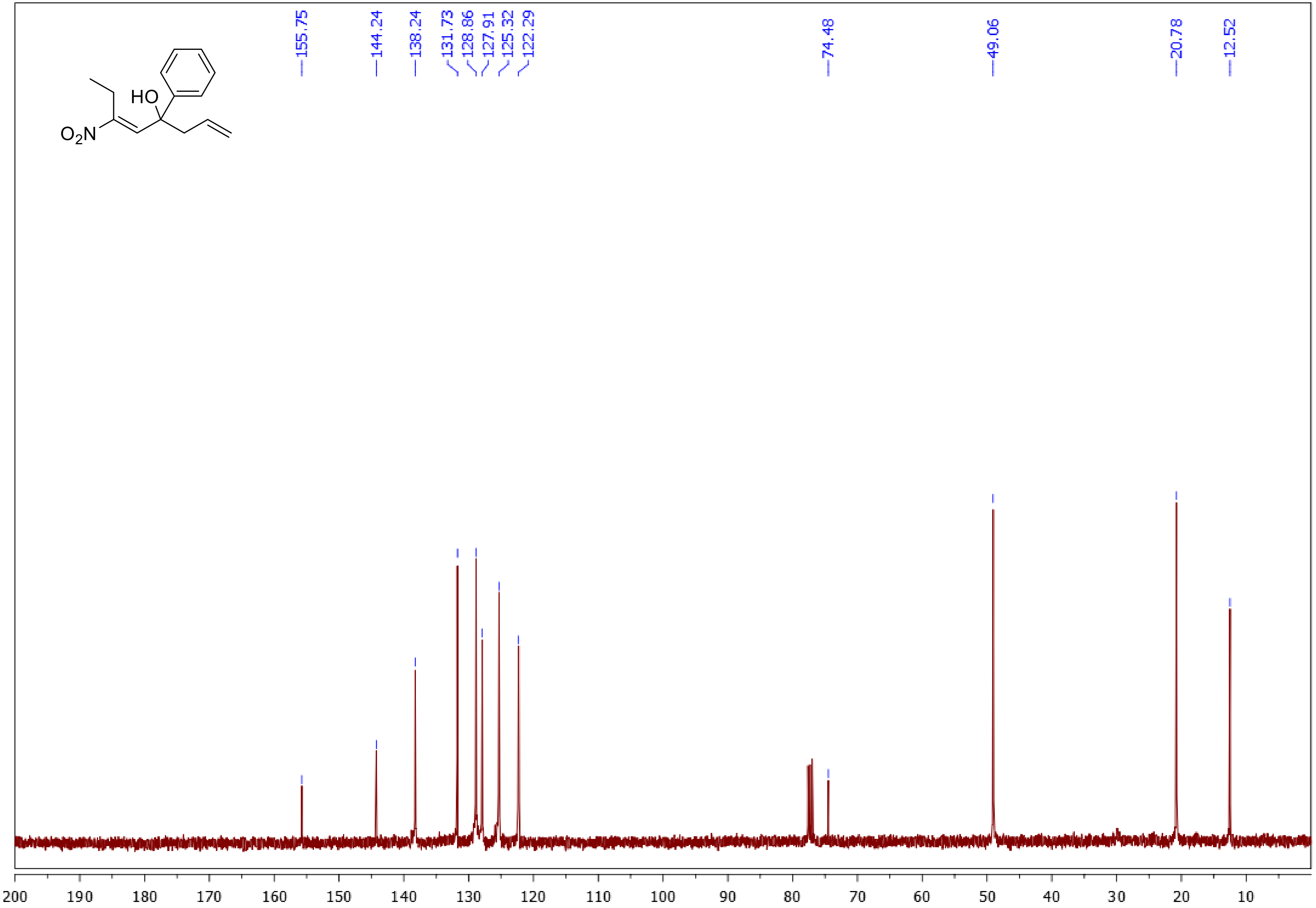

$^1H$ NMR (400 MHz, $CDCl_3$) Compound **3b**.

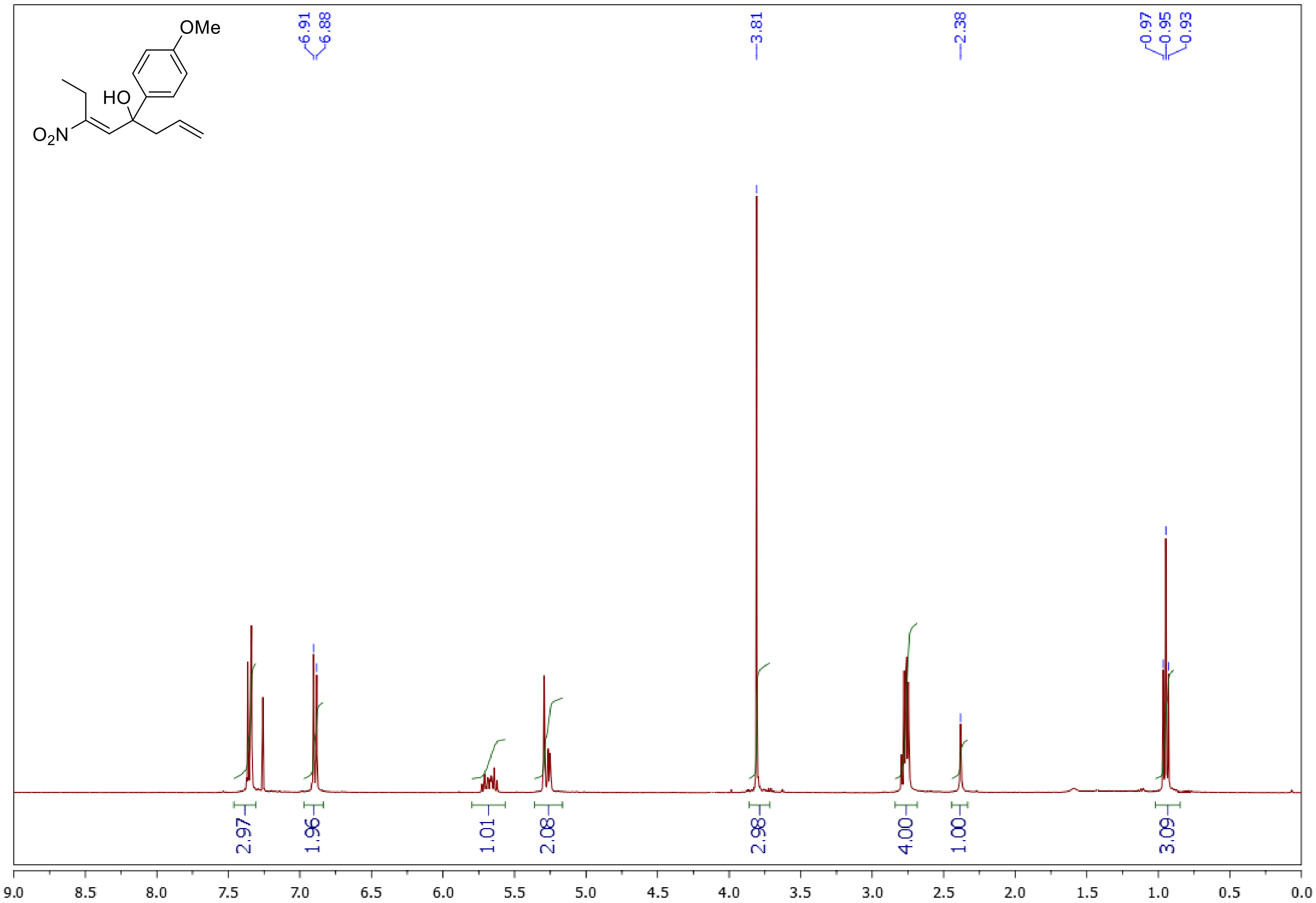

$^{13}C\{^{1}H\}$ NMR (100 MHz, $CDCl_3$) Compound **3b**.

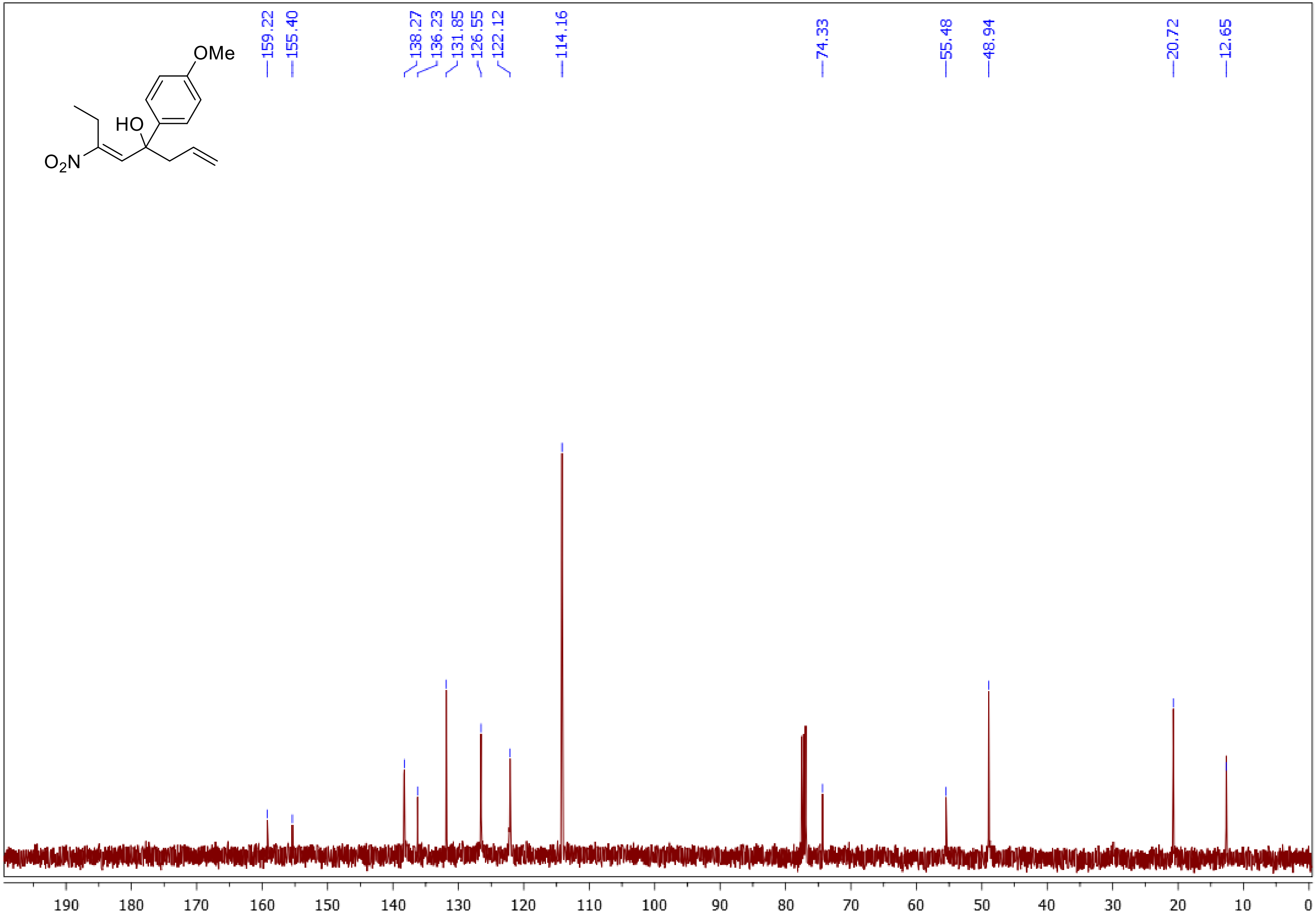

$^1H$ NMR (400 MHz, $CDCl_3$) Compound **3c**.

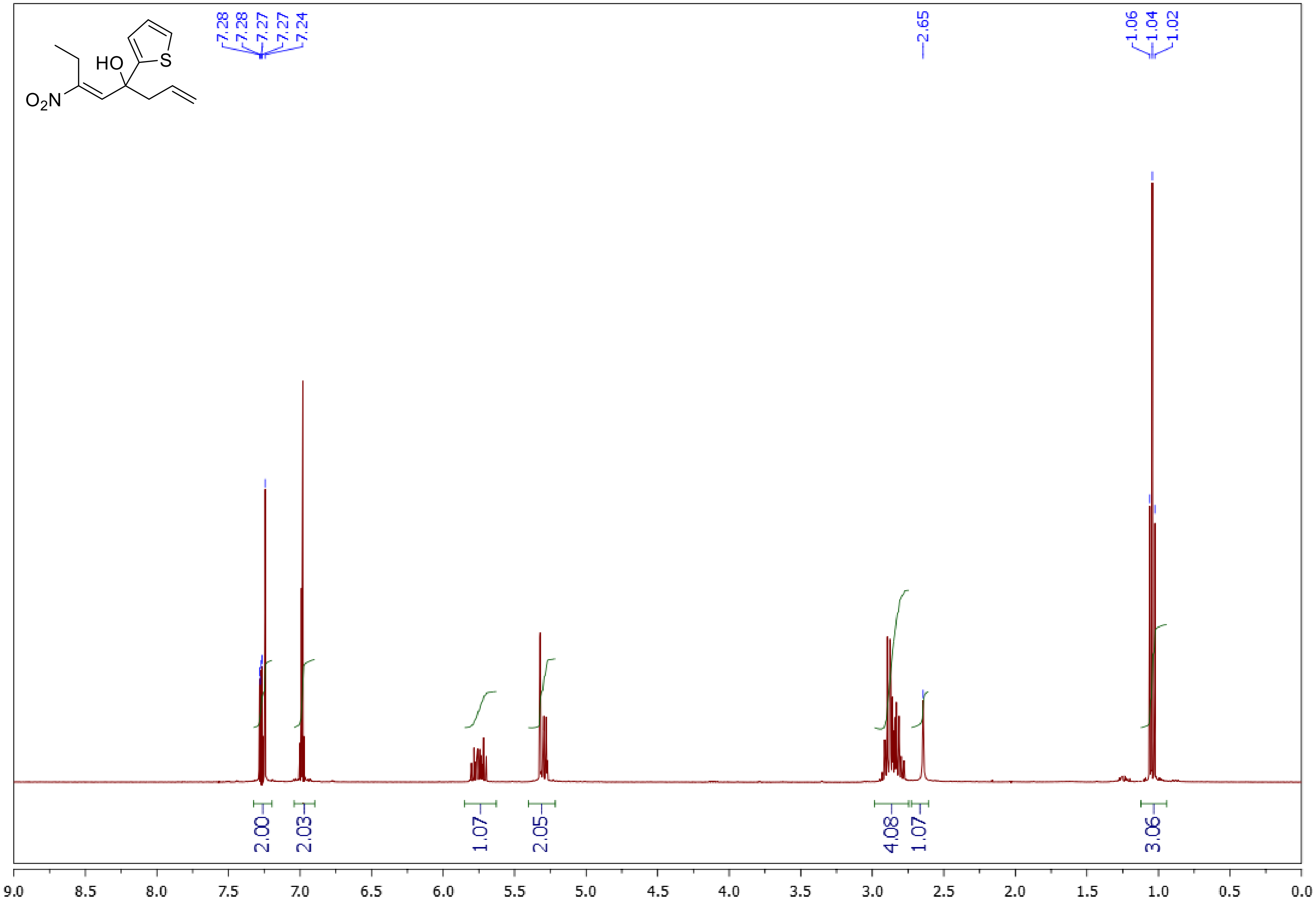

$^{13}C\{^{1}H\}$ NMR (100 MHz, $CDCl_3$) Compound **3c**.

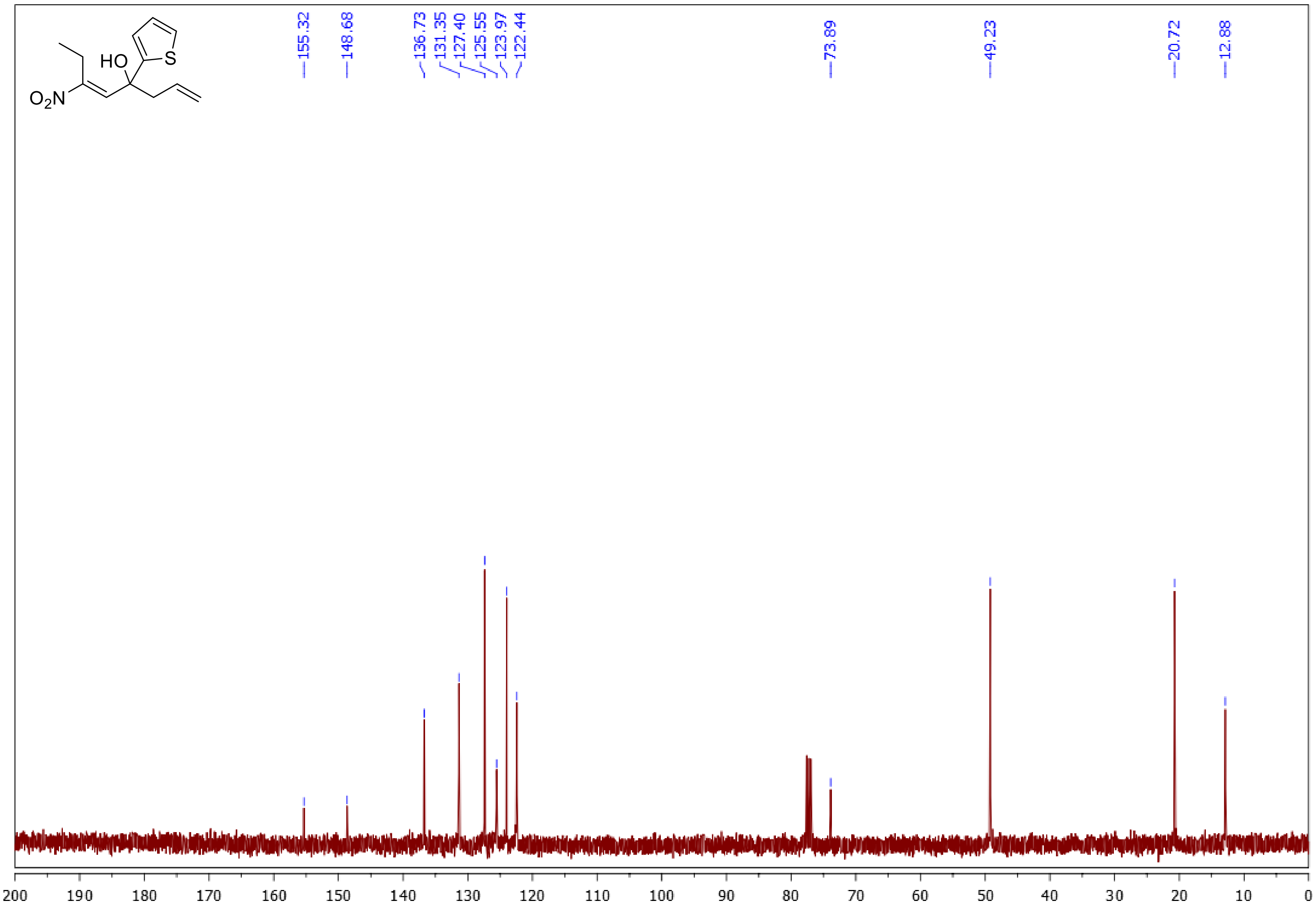

$^{1}H$ NMR (400 MHz, $CDCl_3$) Compound **3d**.

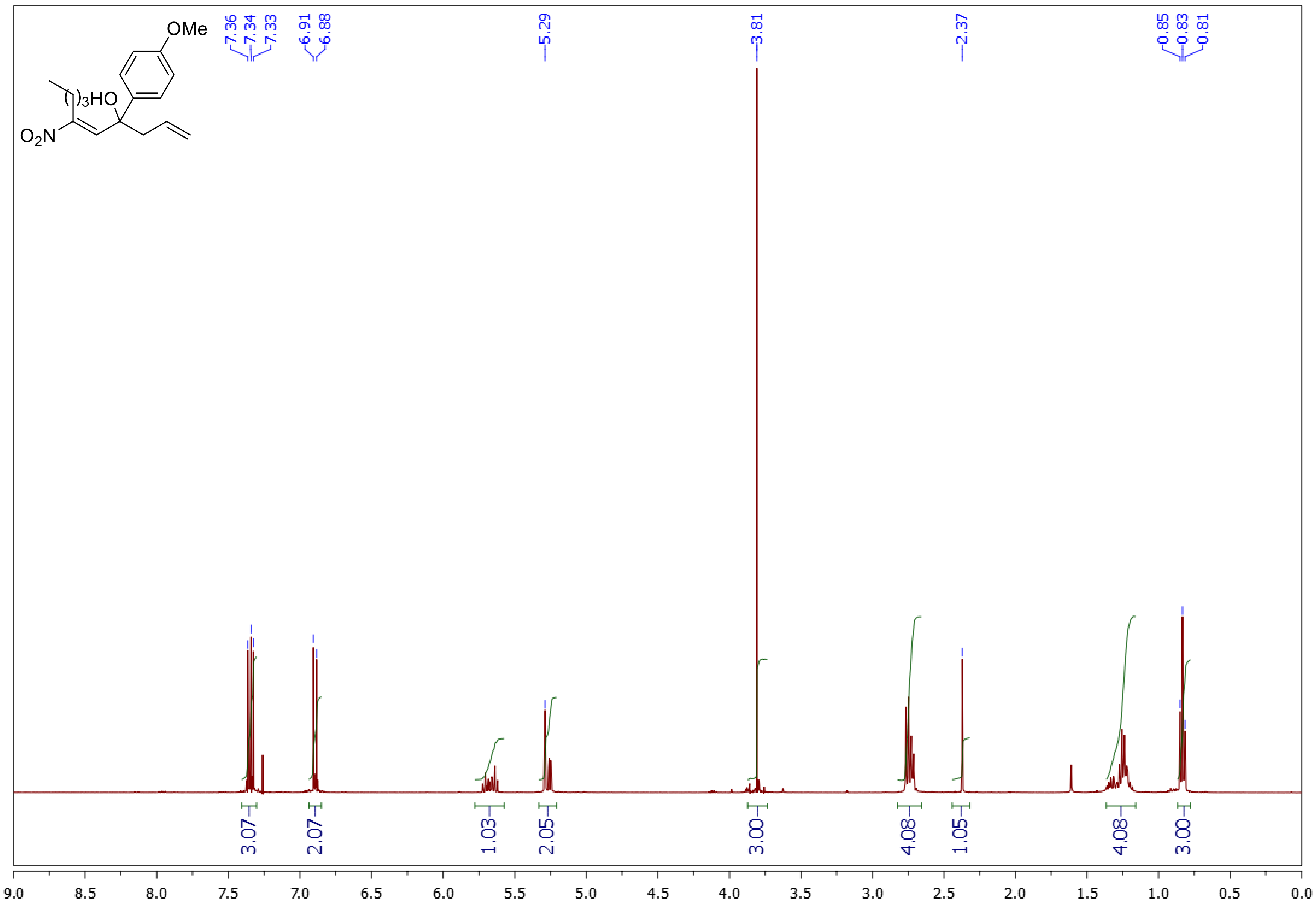

$^{13}C\{^1H\}$ NMR (100 MHz, $CDCl_3$) Compound **3d**.

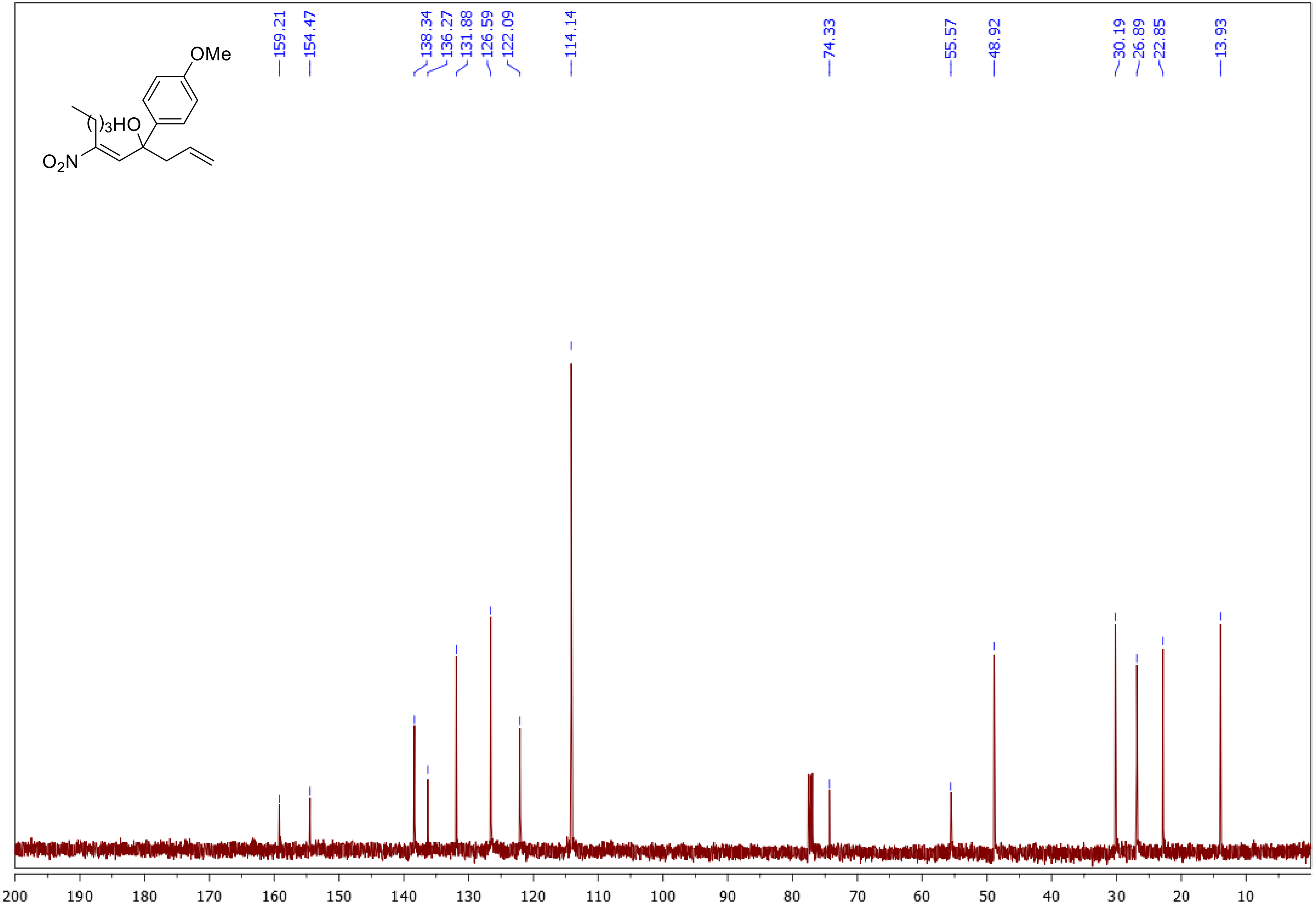

$^{1}$H NMR (400 MHz, $CDCl_3$) Compound **3e**.

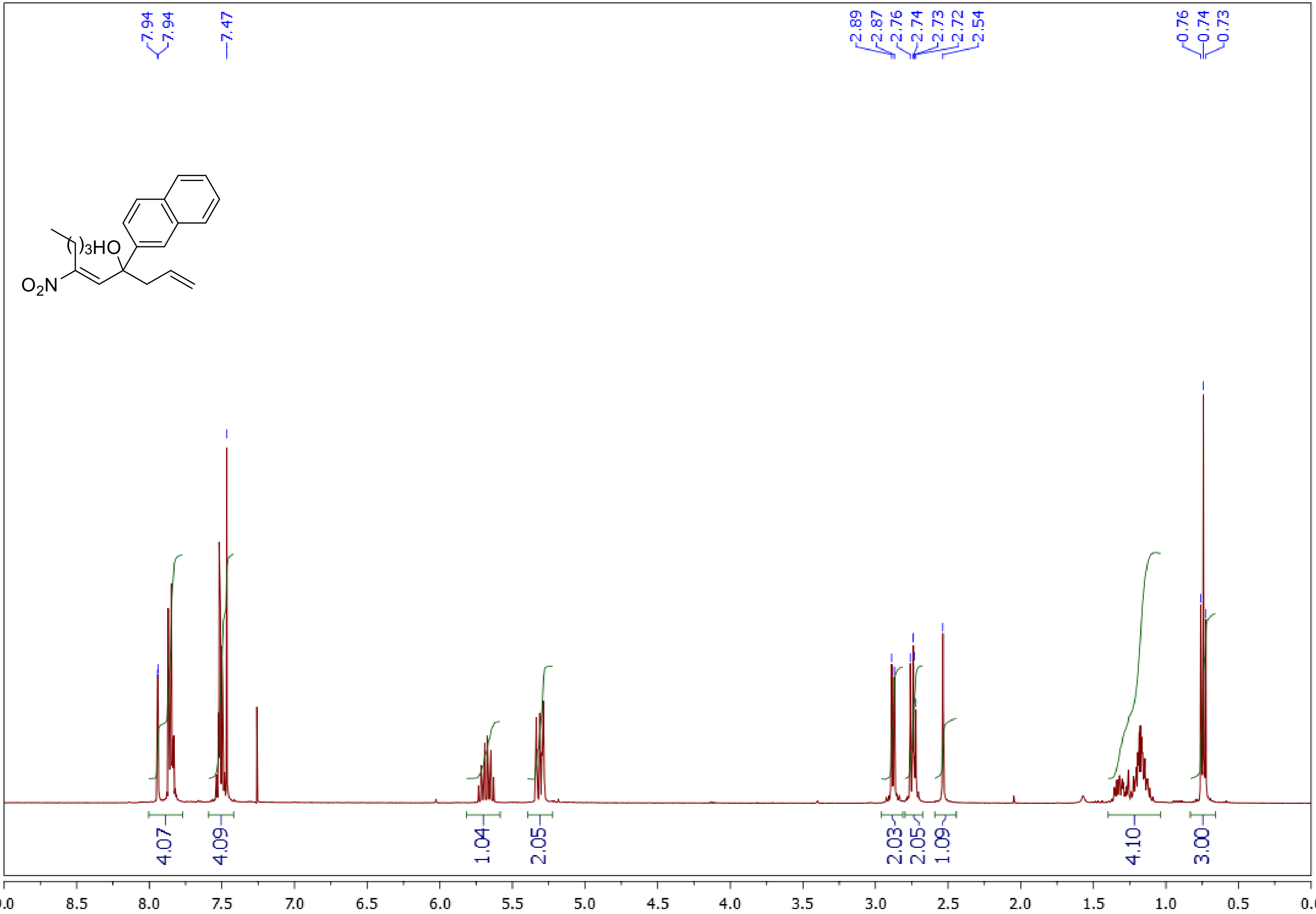

$^{13}C\{^{1}H\}$ NMR (100 MHz, $CDCl_3$) Compound **3e**.

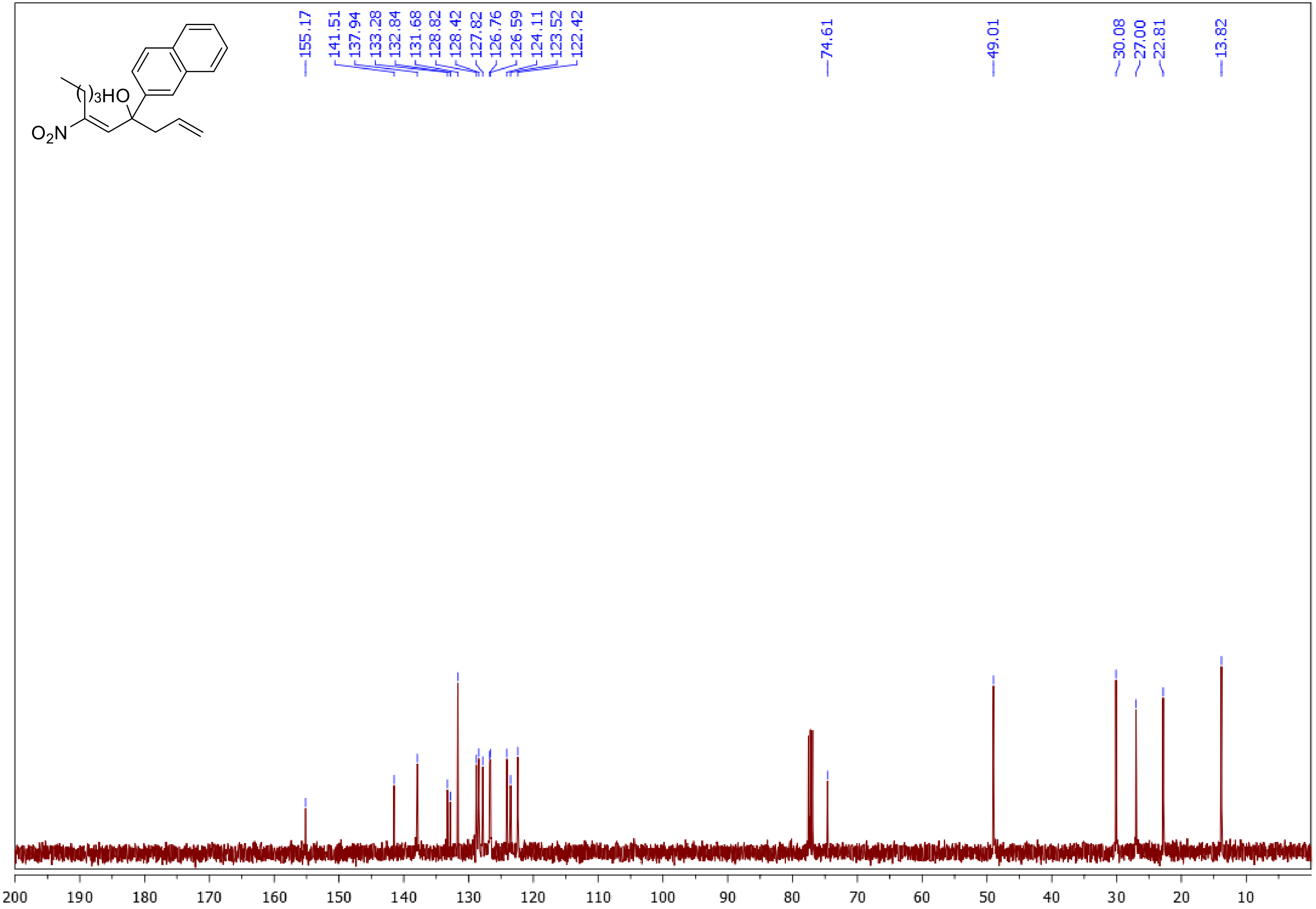

$^1$H NMR (400 MHz, $CDCl_3$) Compound **3f**.

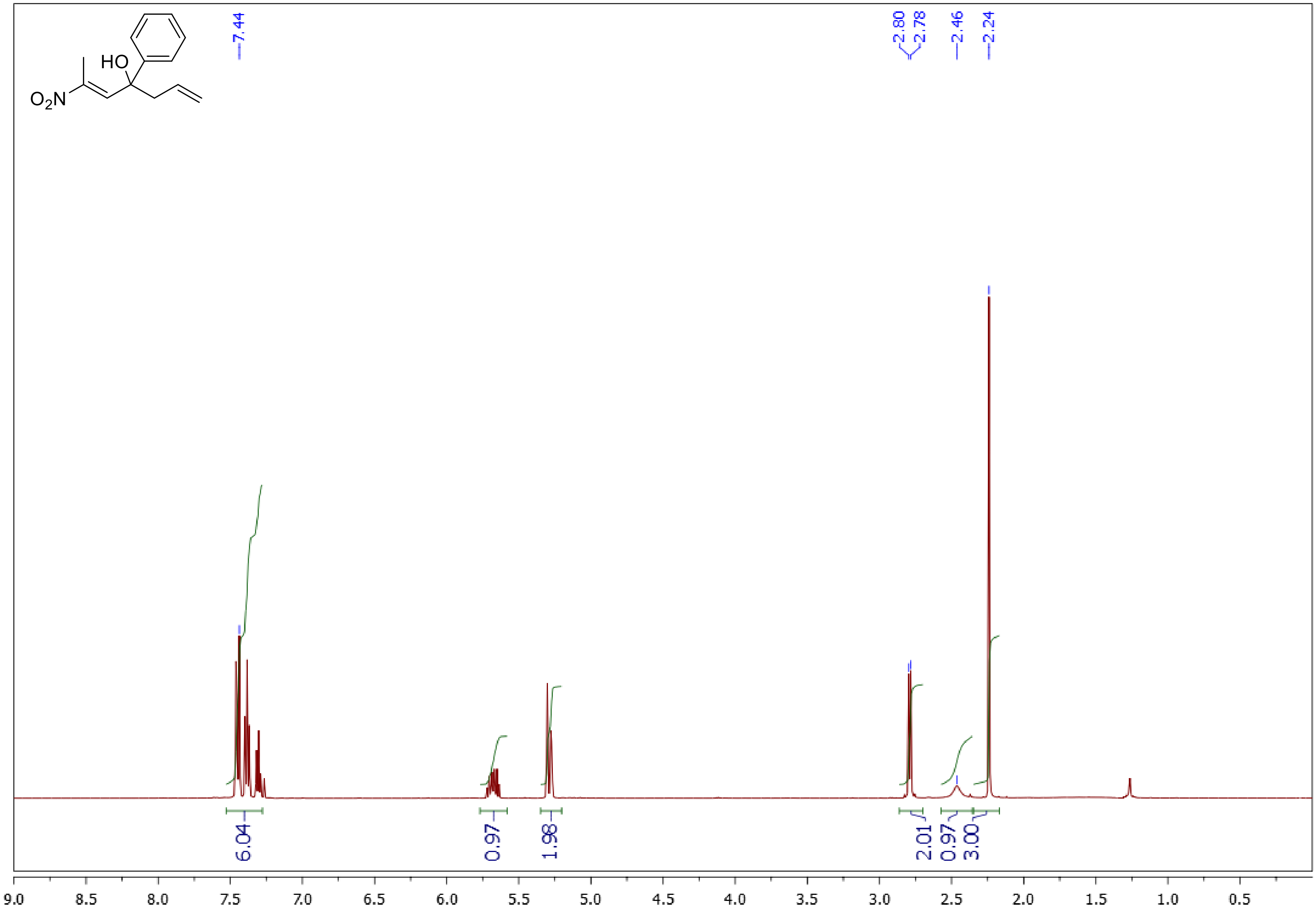

$^{13}C\{^1H\}$ NMR (100 MHz, $CDCl_3$) Compound **3f**.

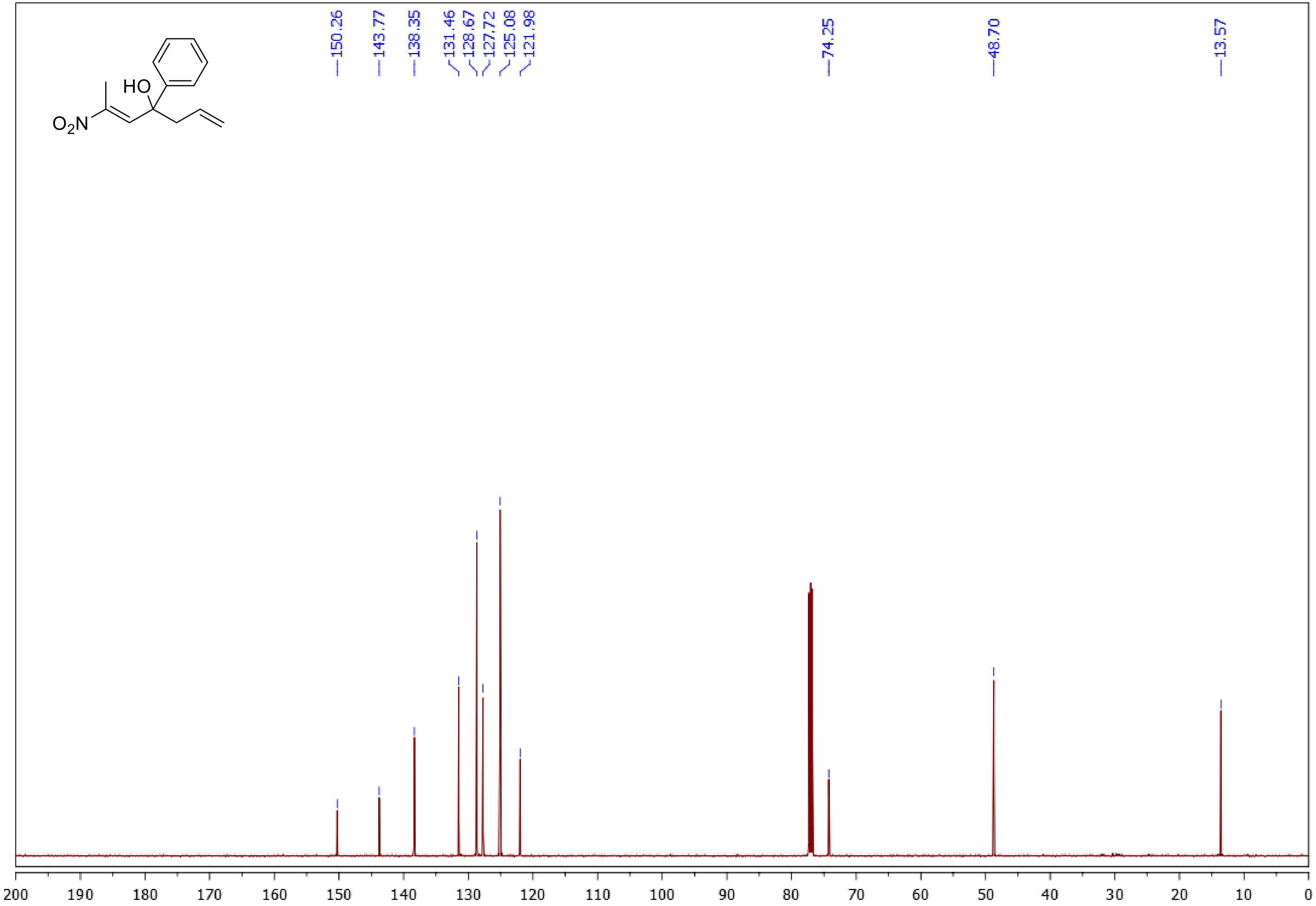

$^{1}$H NMR (400 MHz, $CDCl_3$) Compound **3g**.

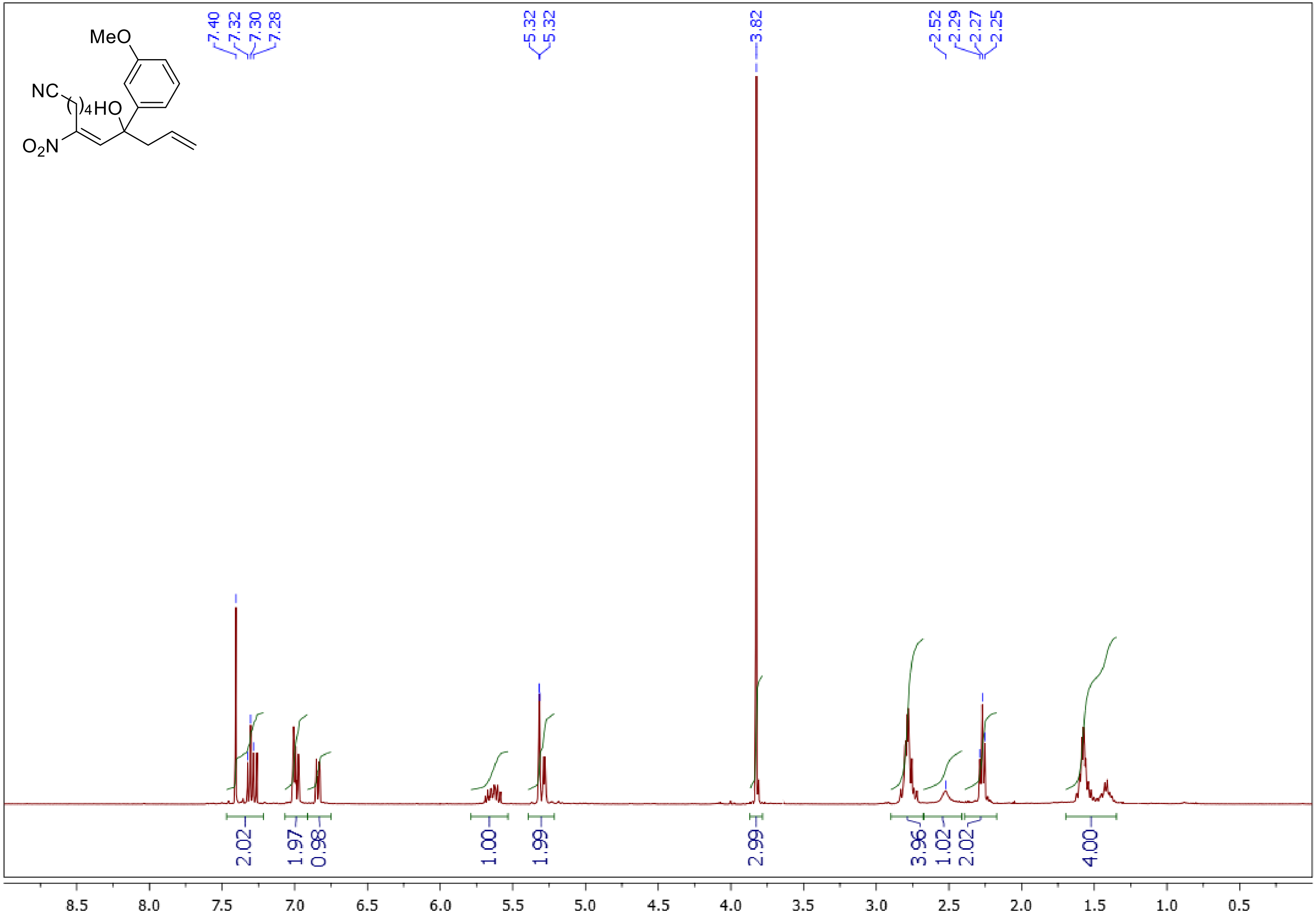

$^{13}C\{^1H\}$ NMR (100 MHz, $CDCl_3$) Compound **3g**.

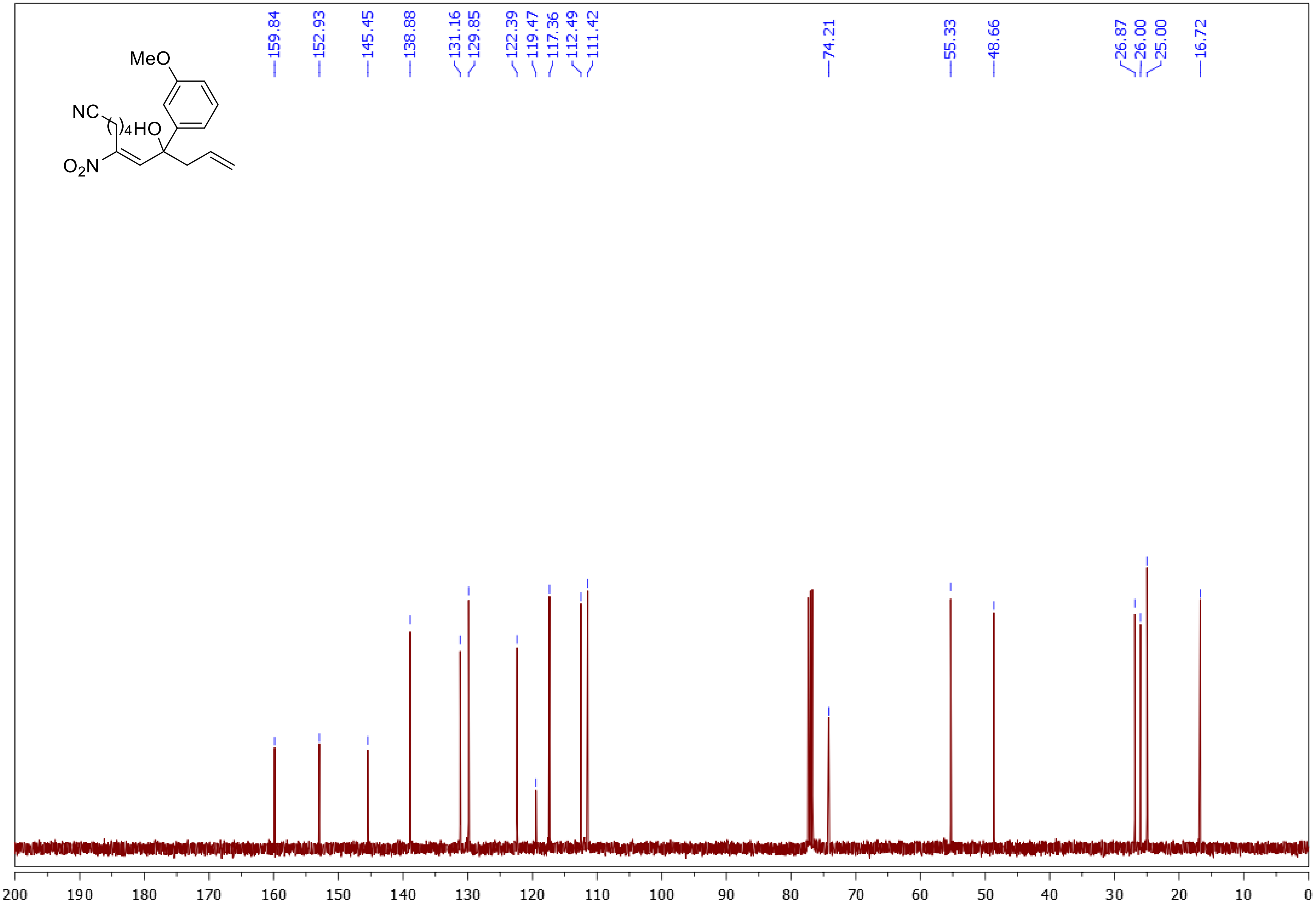

$^{1}H$ NMR (400 MHz, $CDCl_3$) Compound **3h**.

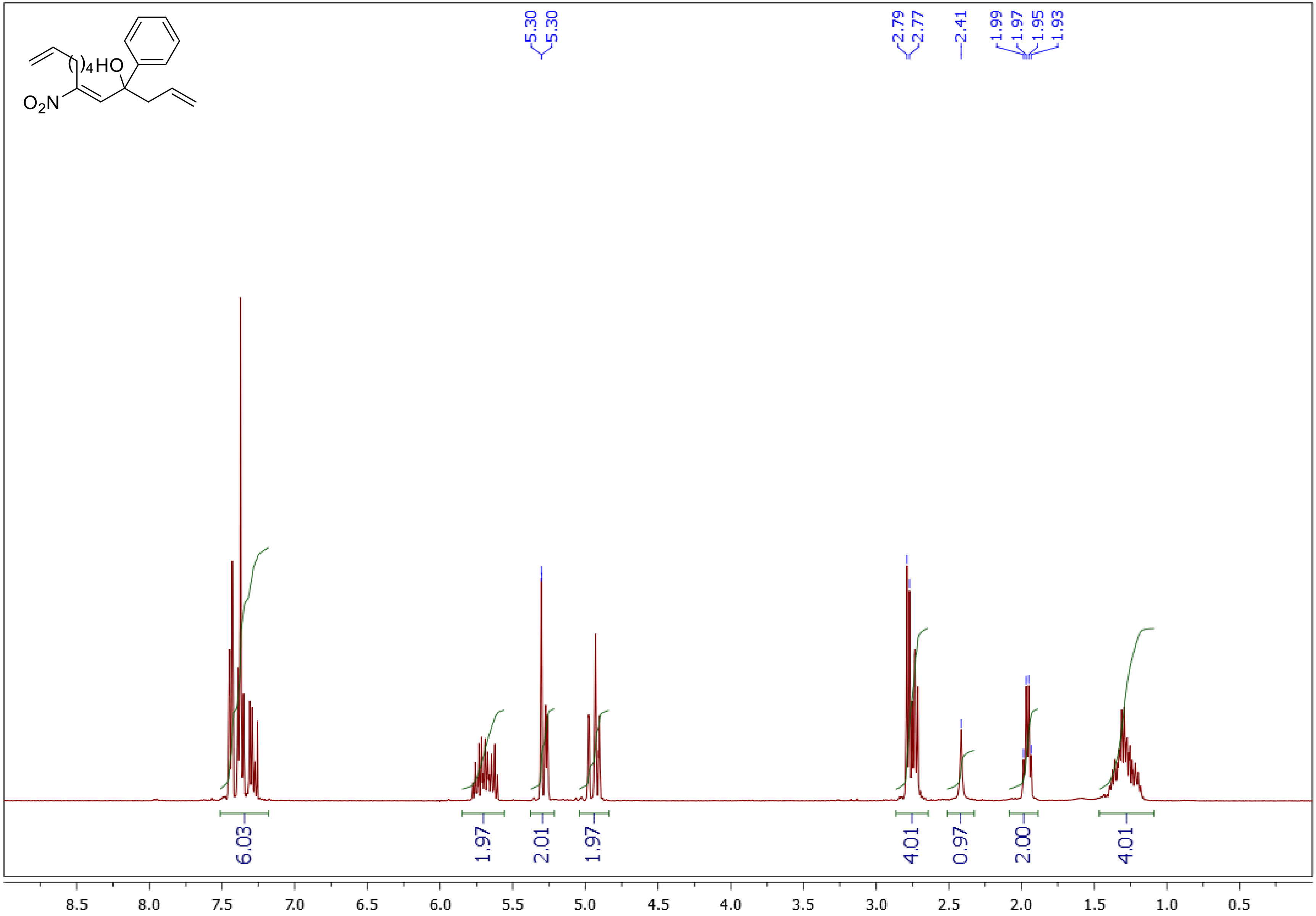

$^{13}C\{^1H\}$ NMR (100 MHz, $CDCl_3$) Compound **3h**.

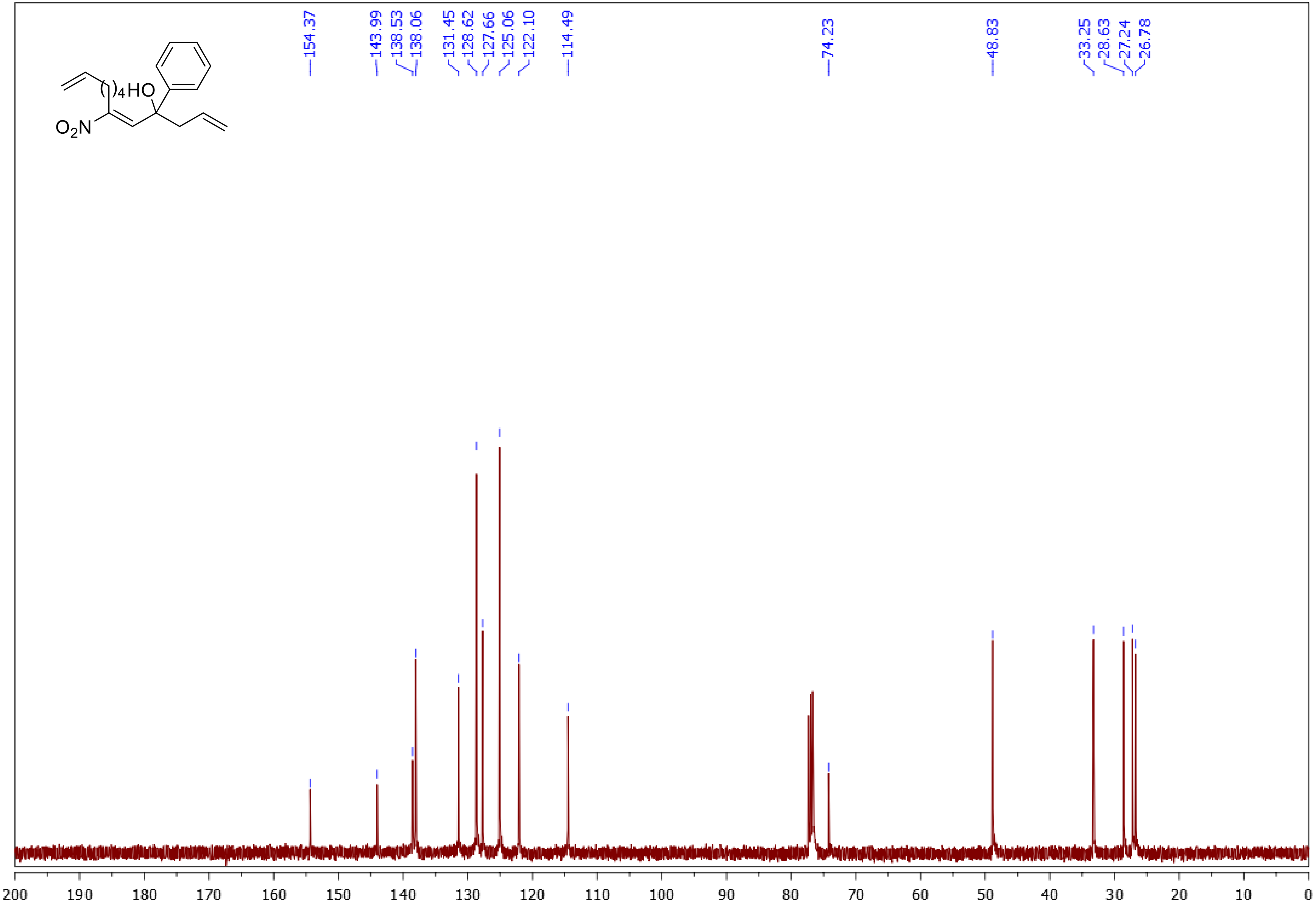

$^{1}$H NMR (400 MHz, $CDCl_3$) Compound **3i**.

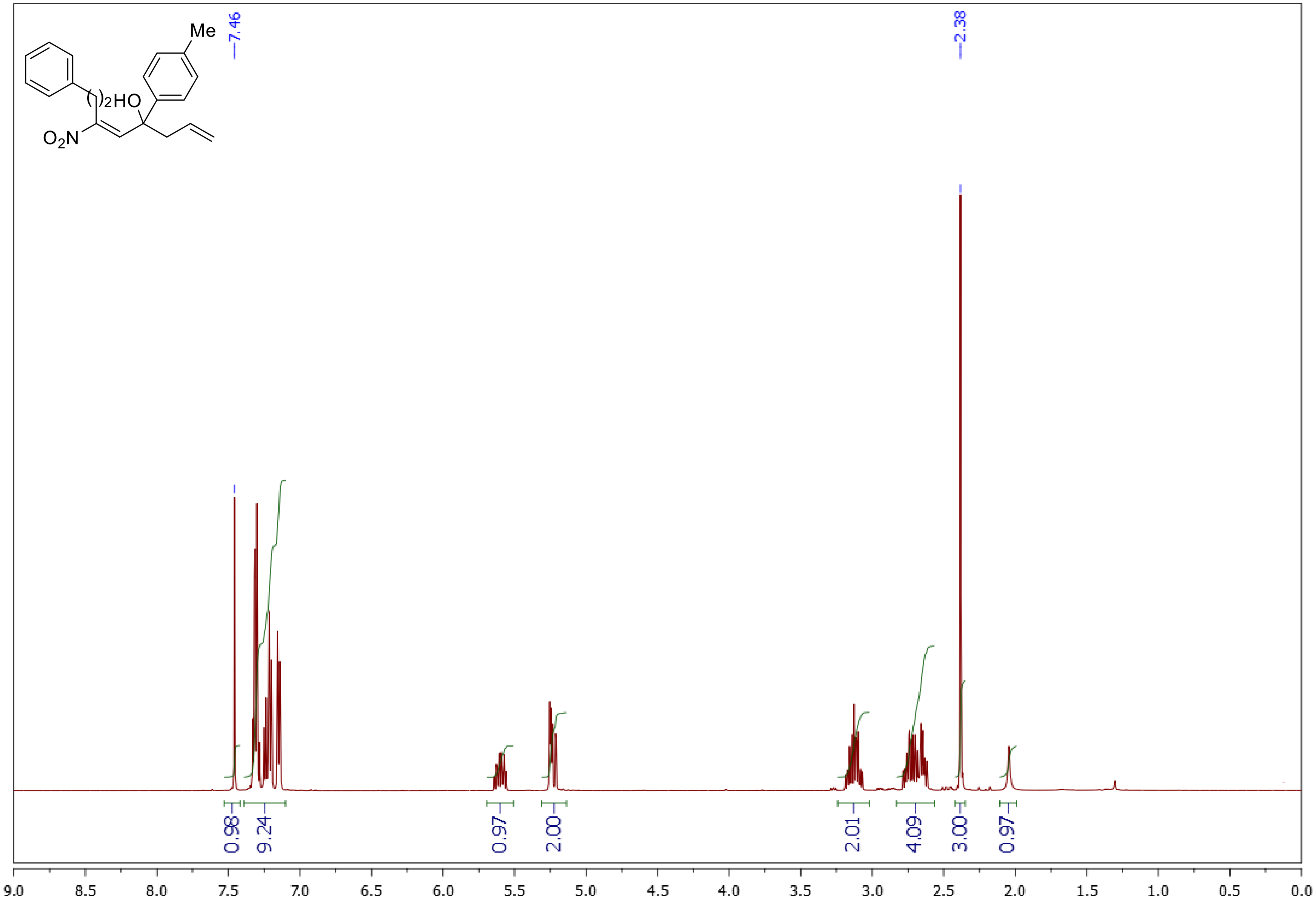

$^{13}C\{^{1}H\}$ NMR (100 MHz, $CDCl_3$) Compound **3i**.

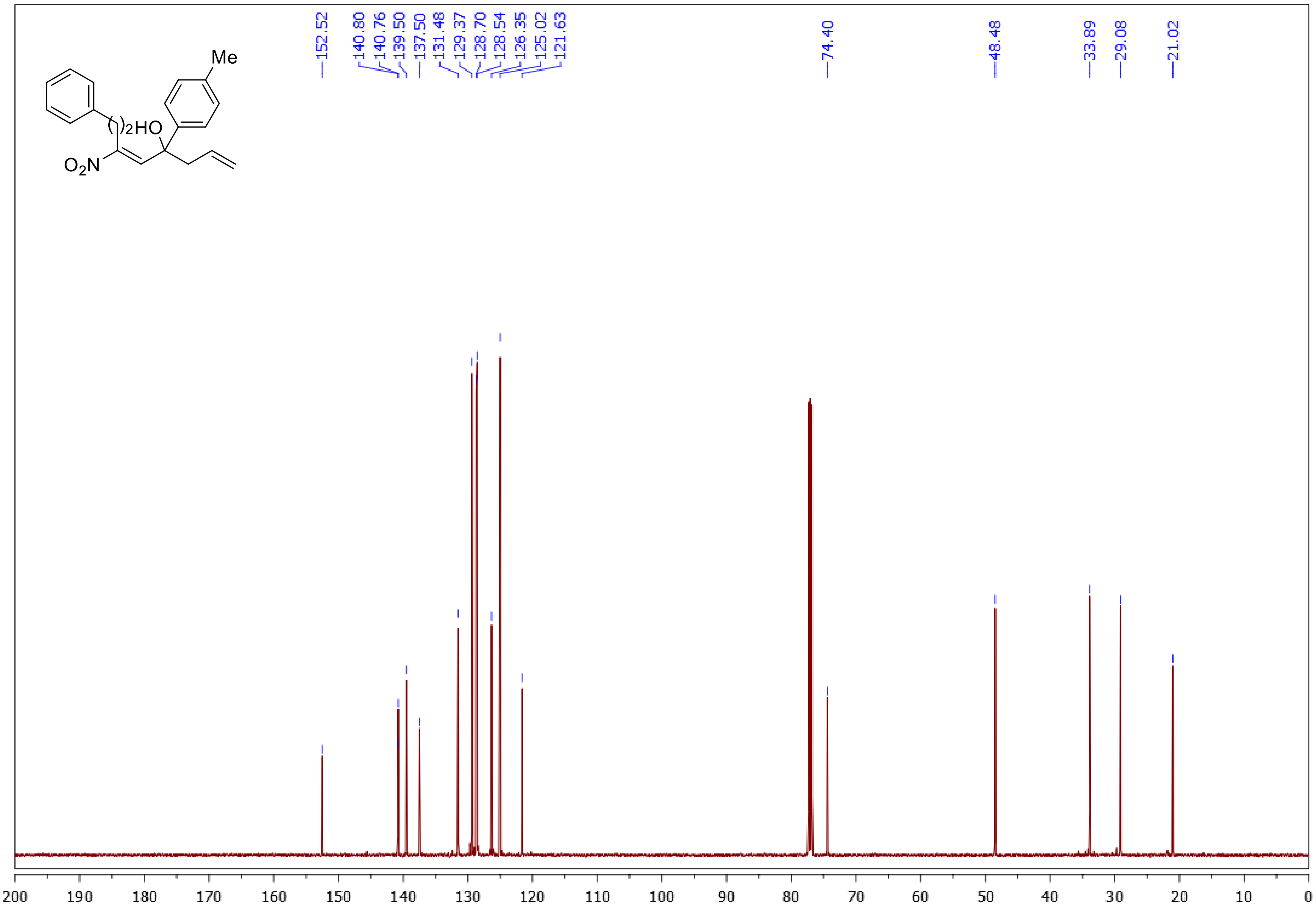

$^1H$ NMR (400 MHz, $CDCl_3$) Compound **3j**.

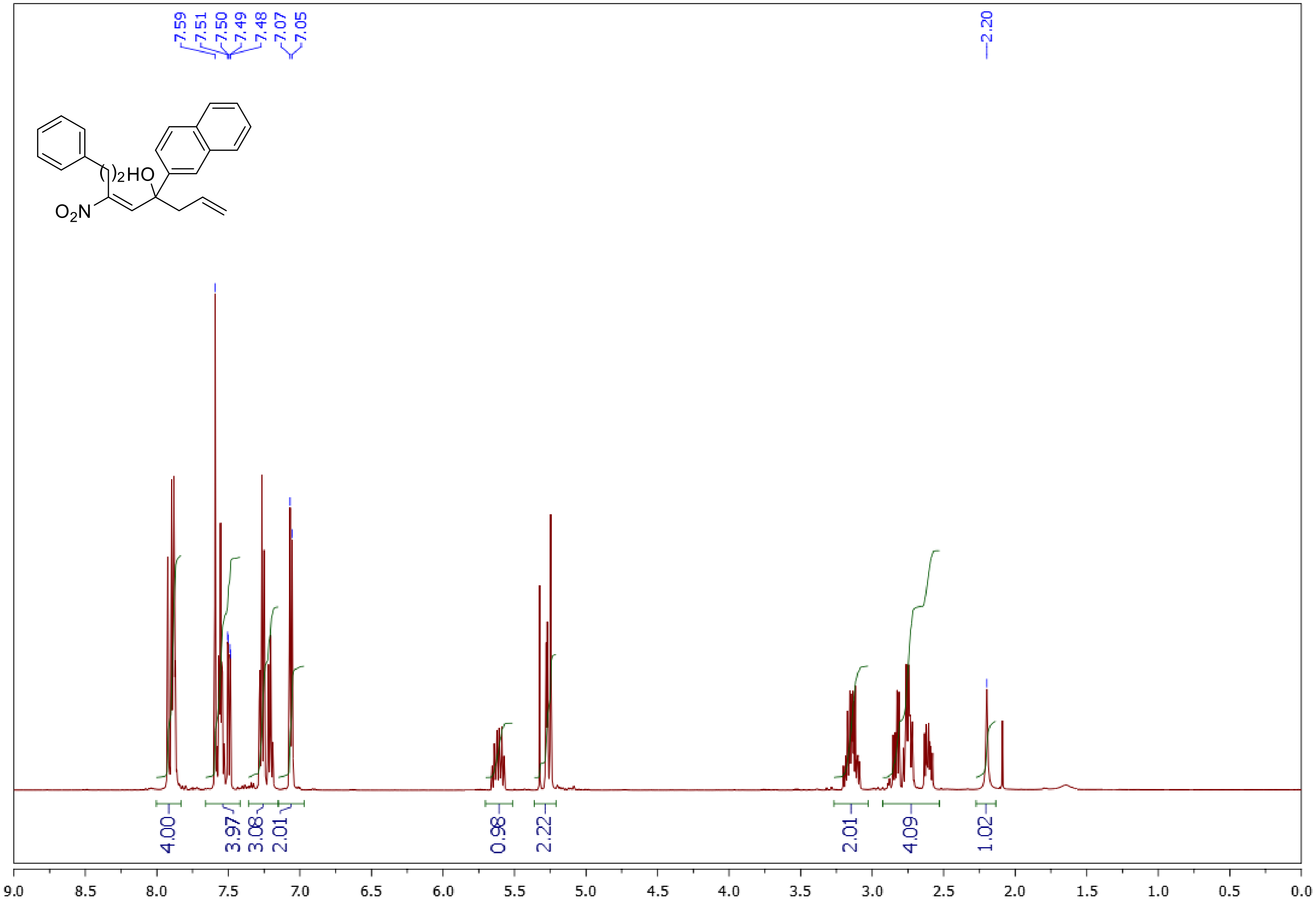

$^{13}C\{^{1}H\}$ NMR (100 MHz, $CDCl_3$) Compound **3j**.

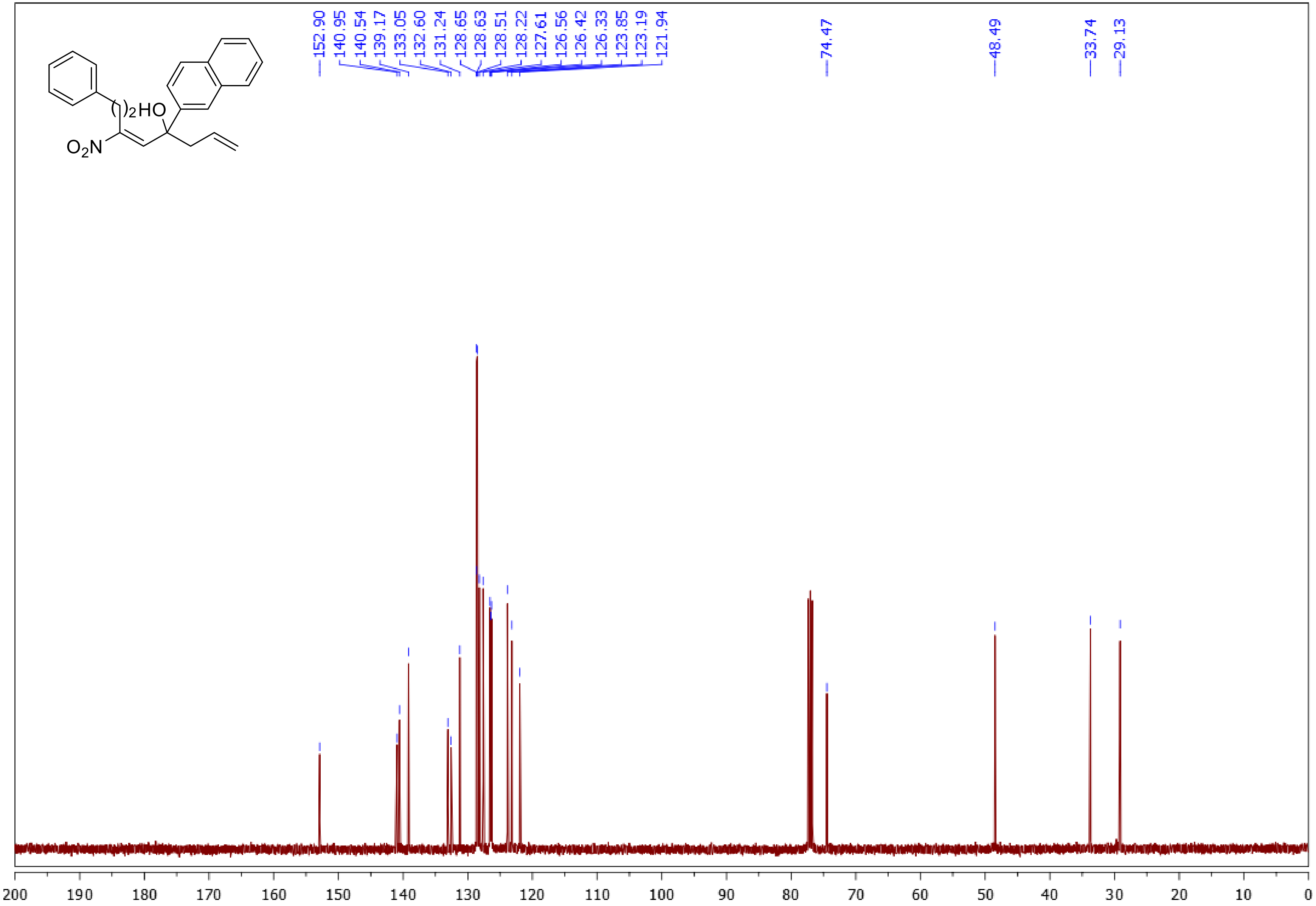

$^{1}H$ NMR (400 MHz, $CDCl_3$) Compound **3k**.

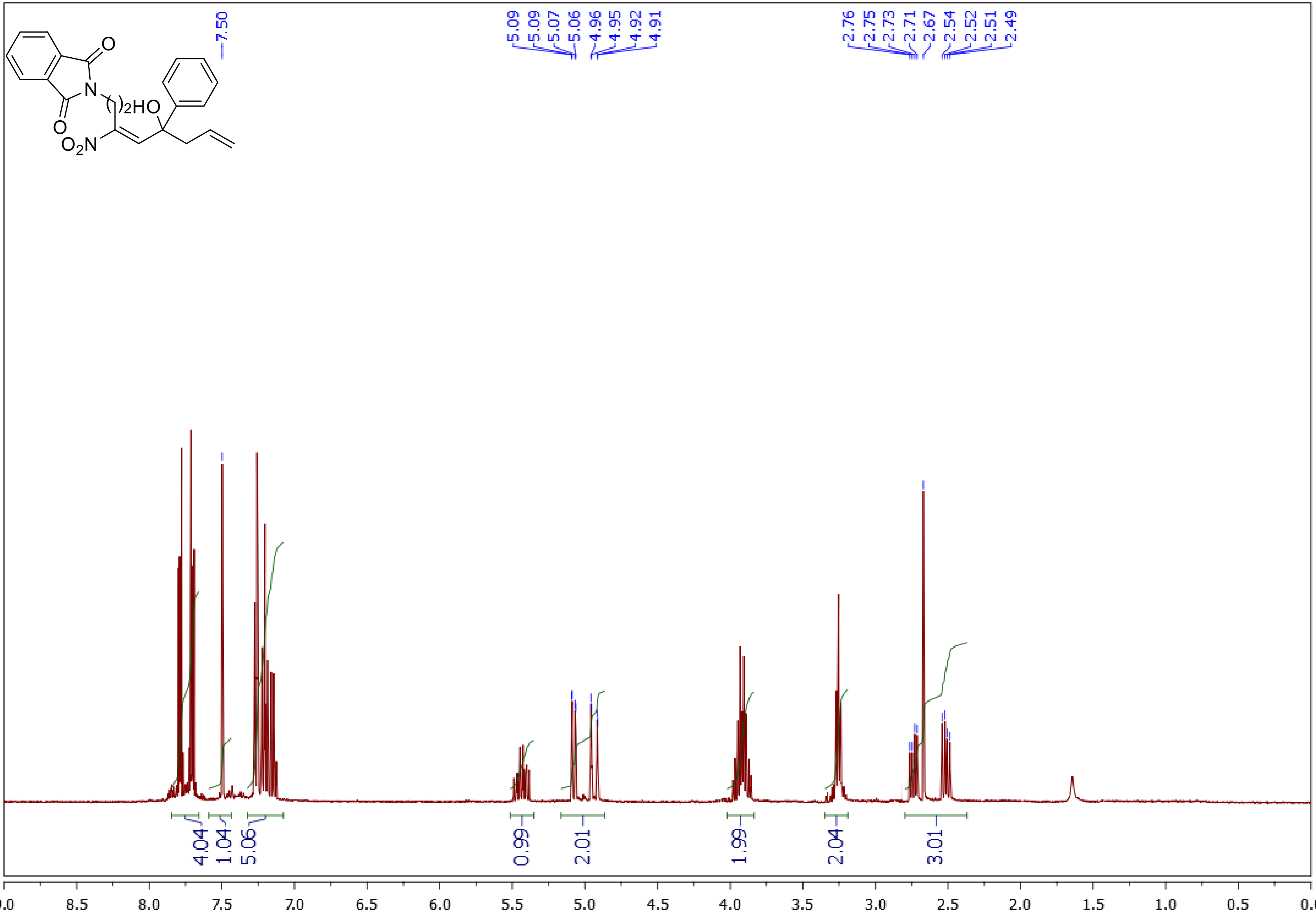

$^{13}C\{^1H\}$ NMR (100 MHz, $CDCl_3$) Compound **3k**.

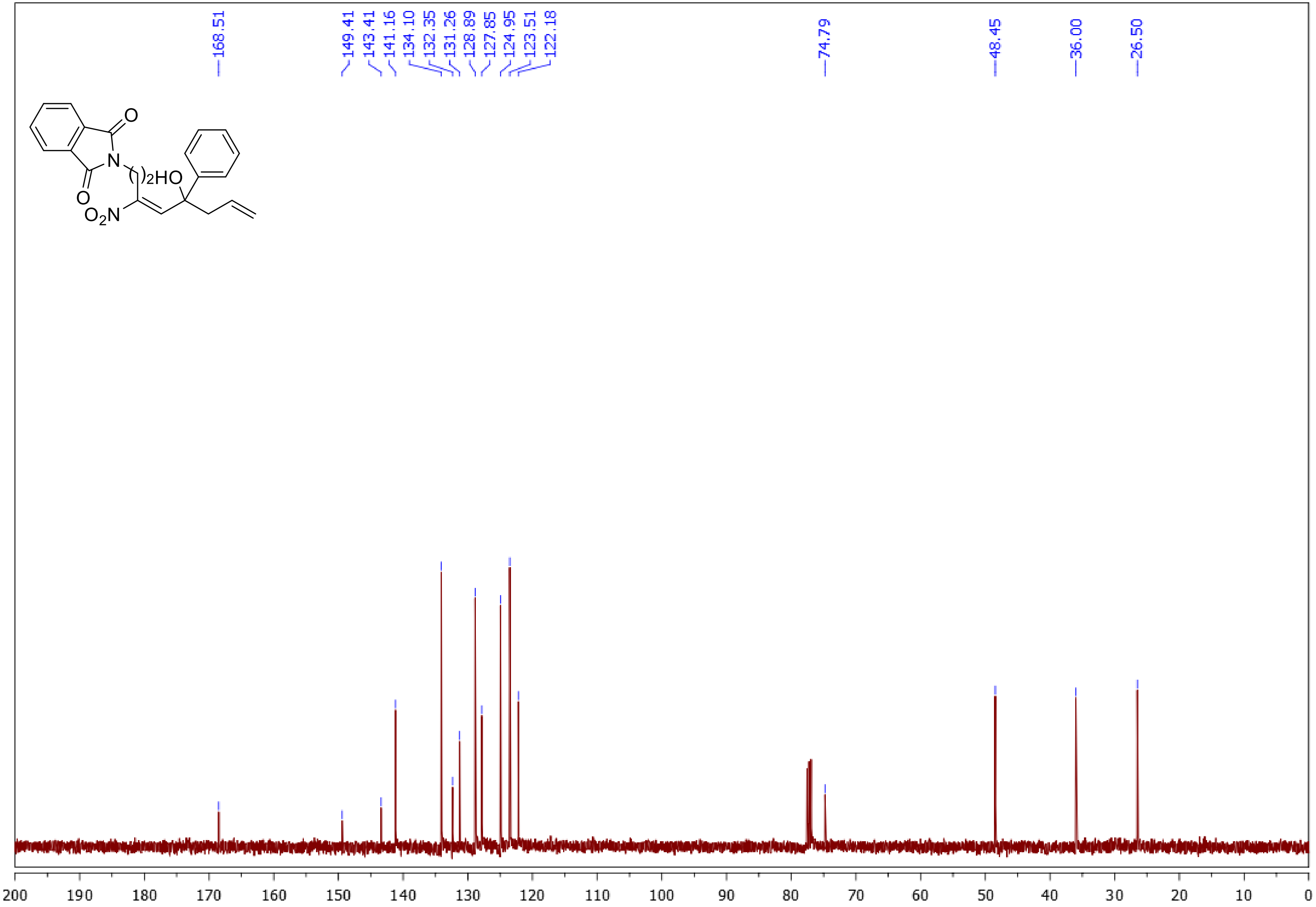

$^{1}$H NMR (400 MHz, $CDCl_3$) Compound **3l**.

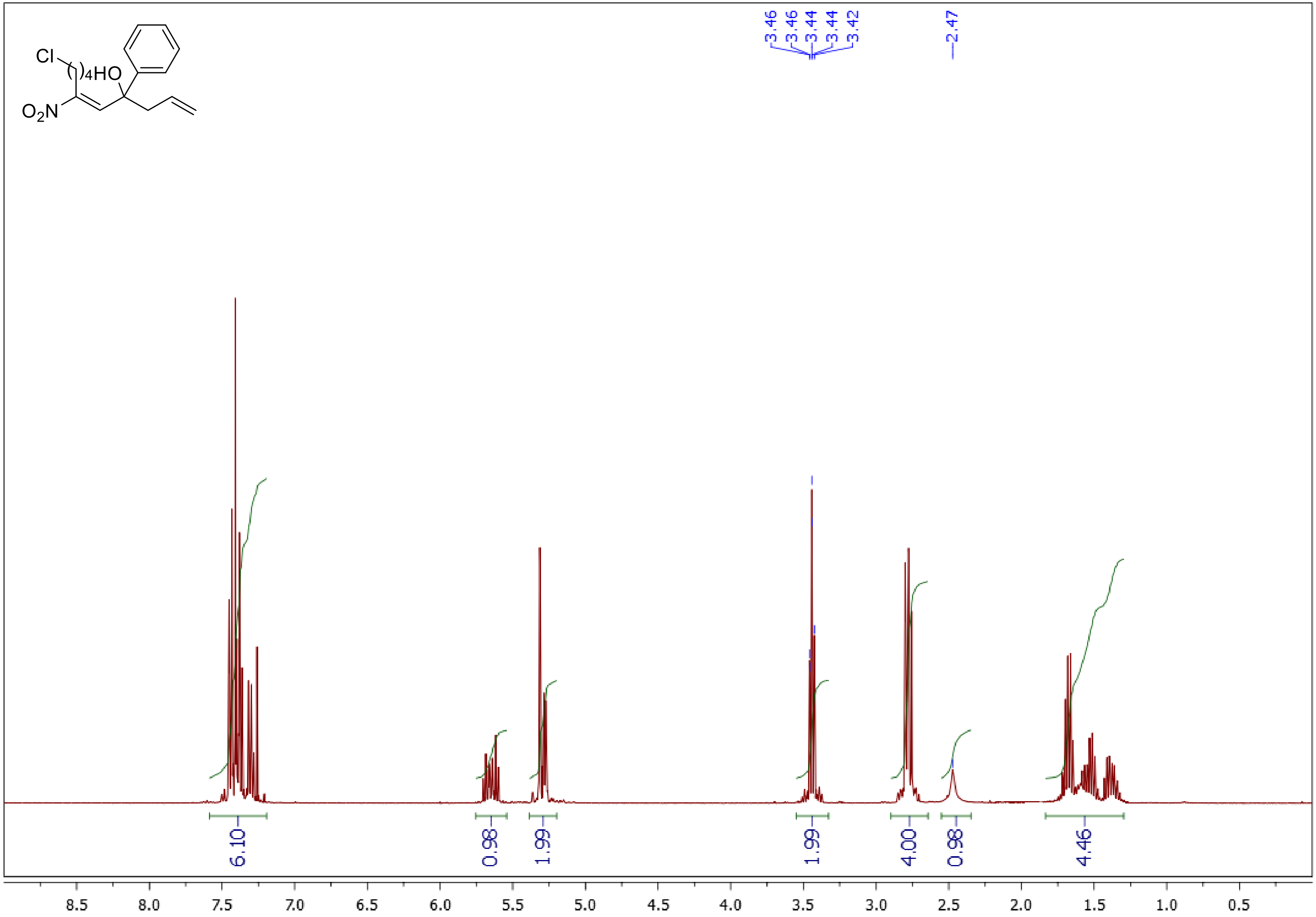

$^{13}C\{^1H\}$ NMR (100 MHz, $CDCl_3$) Compound **3l**.

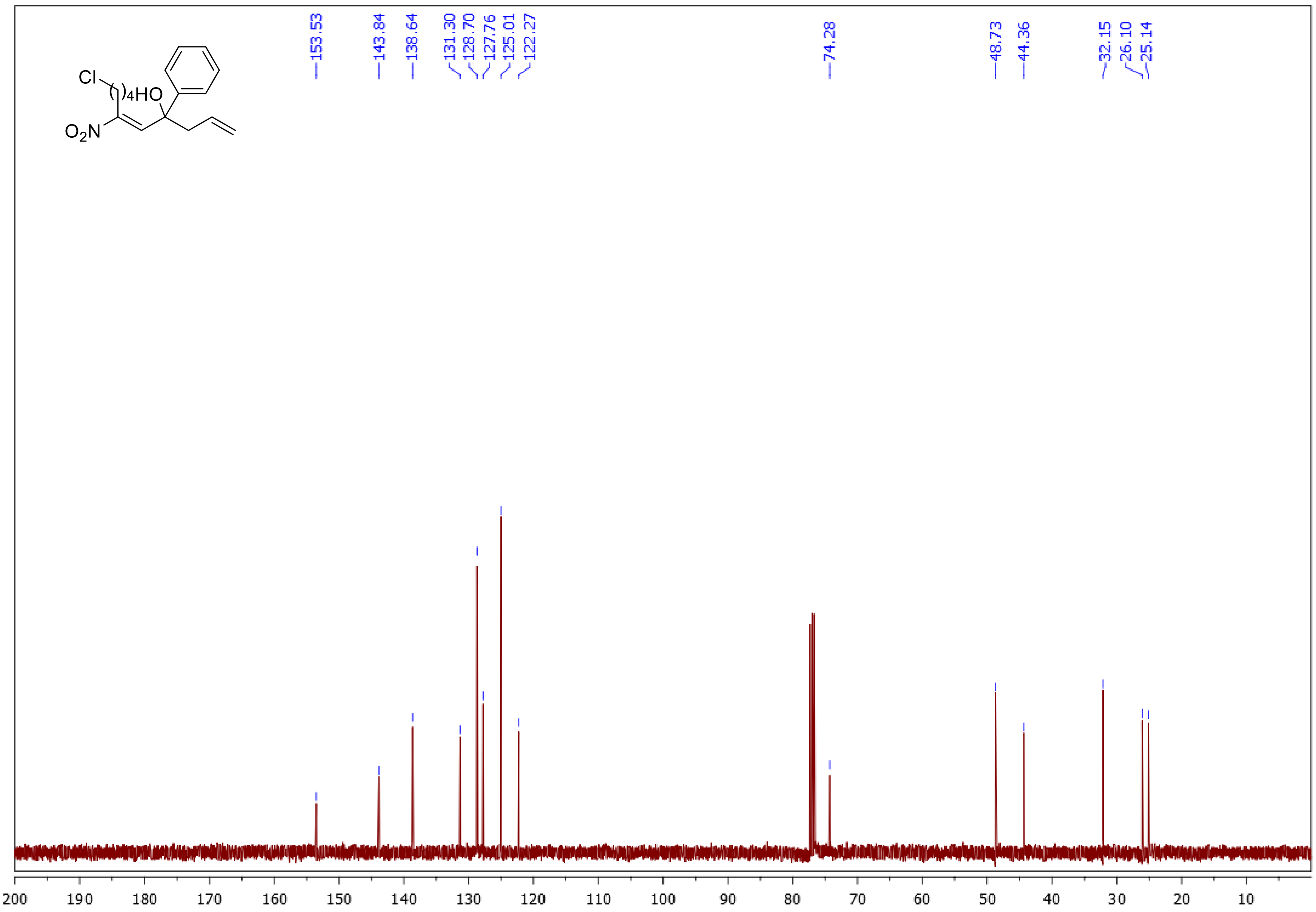

$^{1}$H NMR (400 MHz, $CDCl_3$) Compound **3m**.

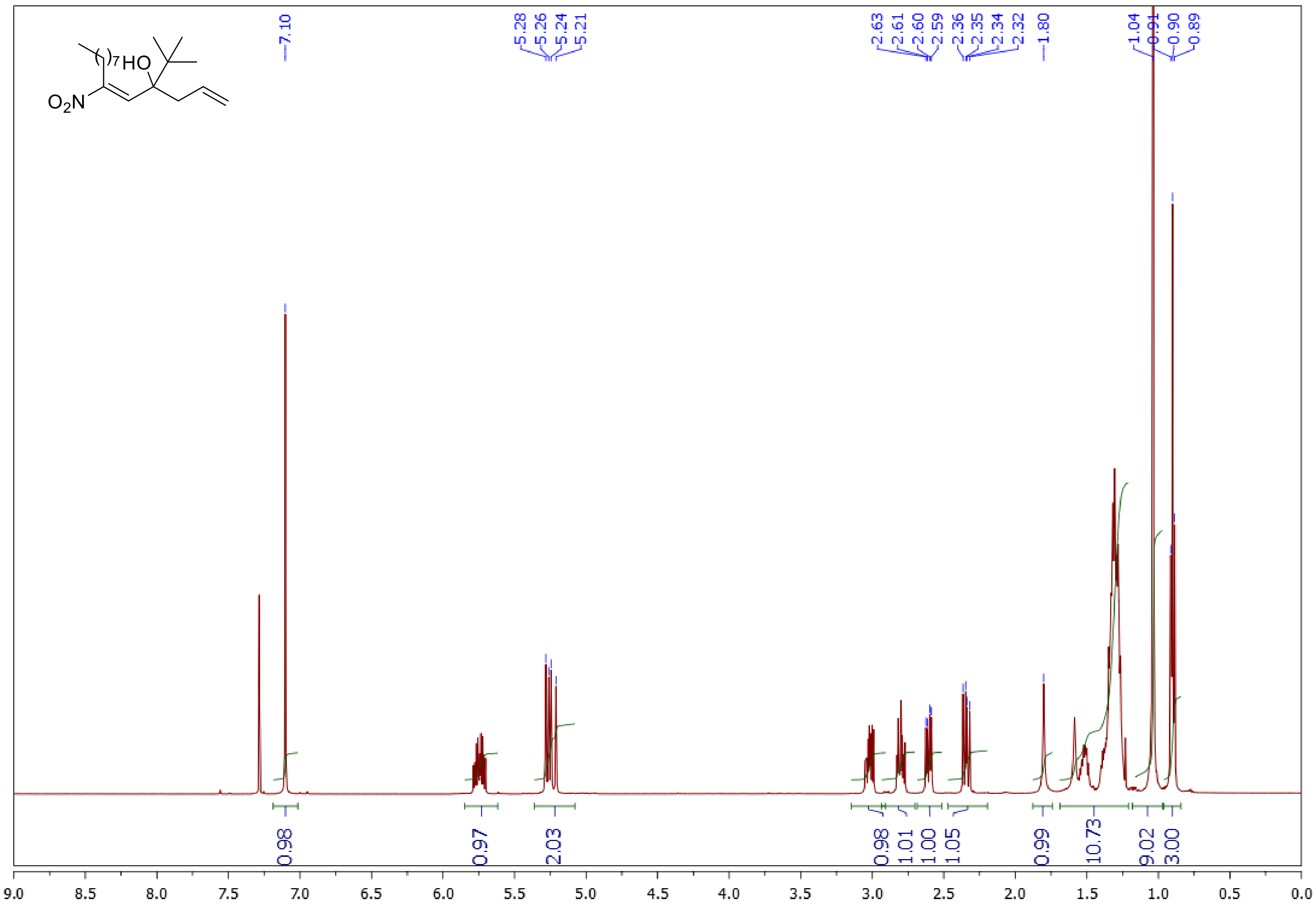

$^{13}C\{^{1}H\}$ NMR (100 MHz, $CDCl_3$) Compound **3m**.

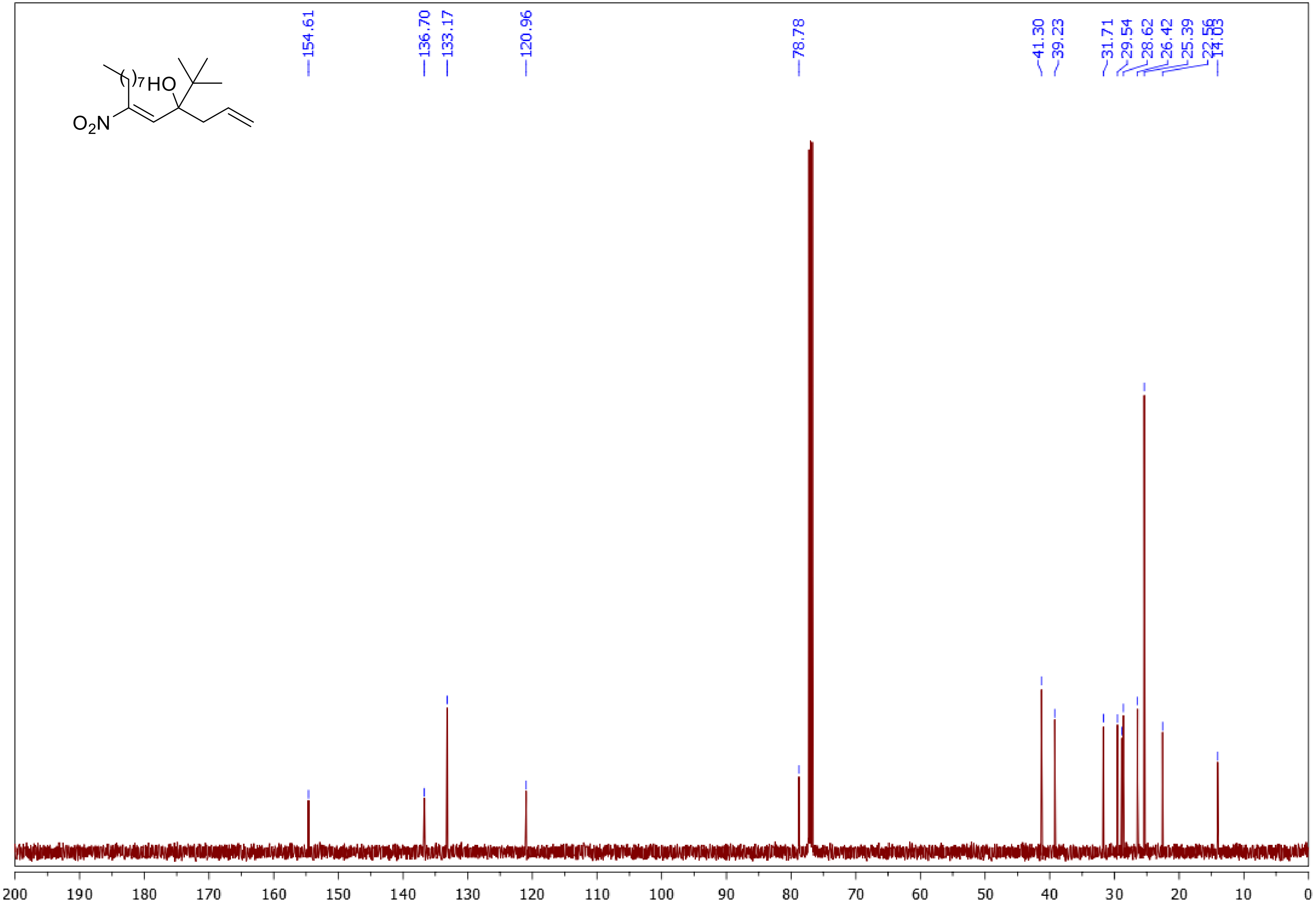

$^1$H NMR (400 MHz, $CDCl_3$) Compound **3n**.

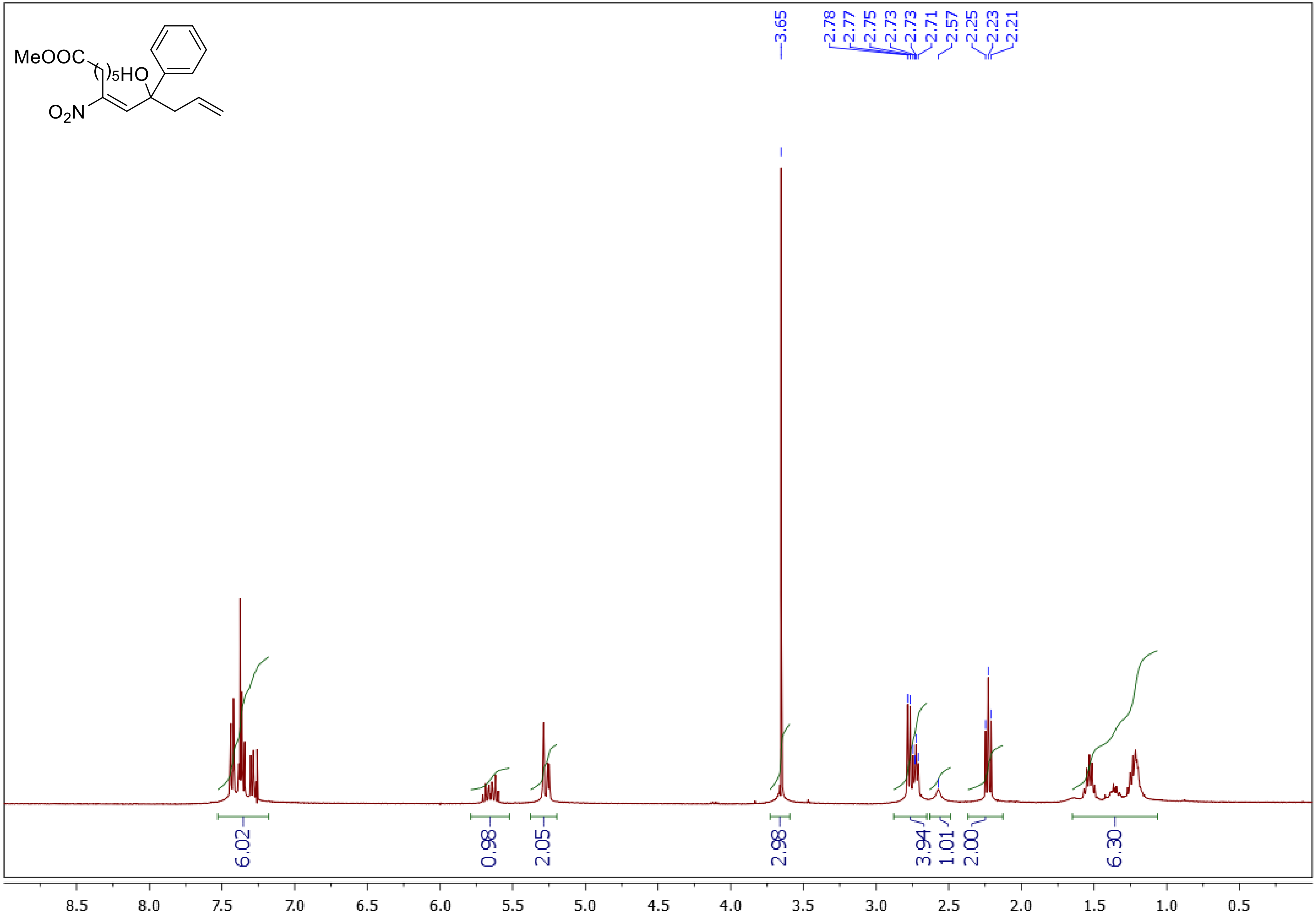

$^{13}C\{^{1}H\}$ NMR (100 MHz, $CDCl_3$) Compound **3n**.

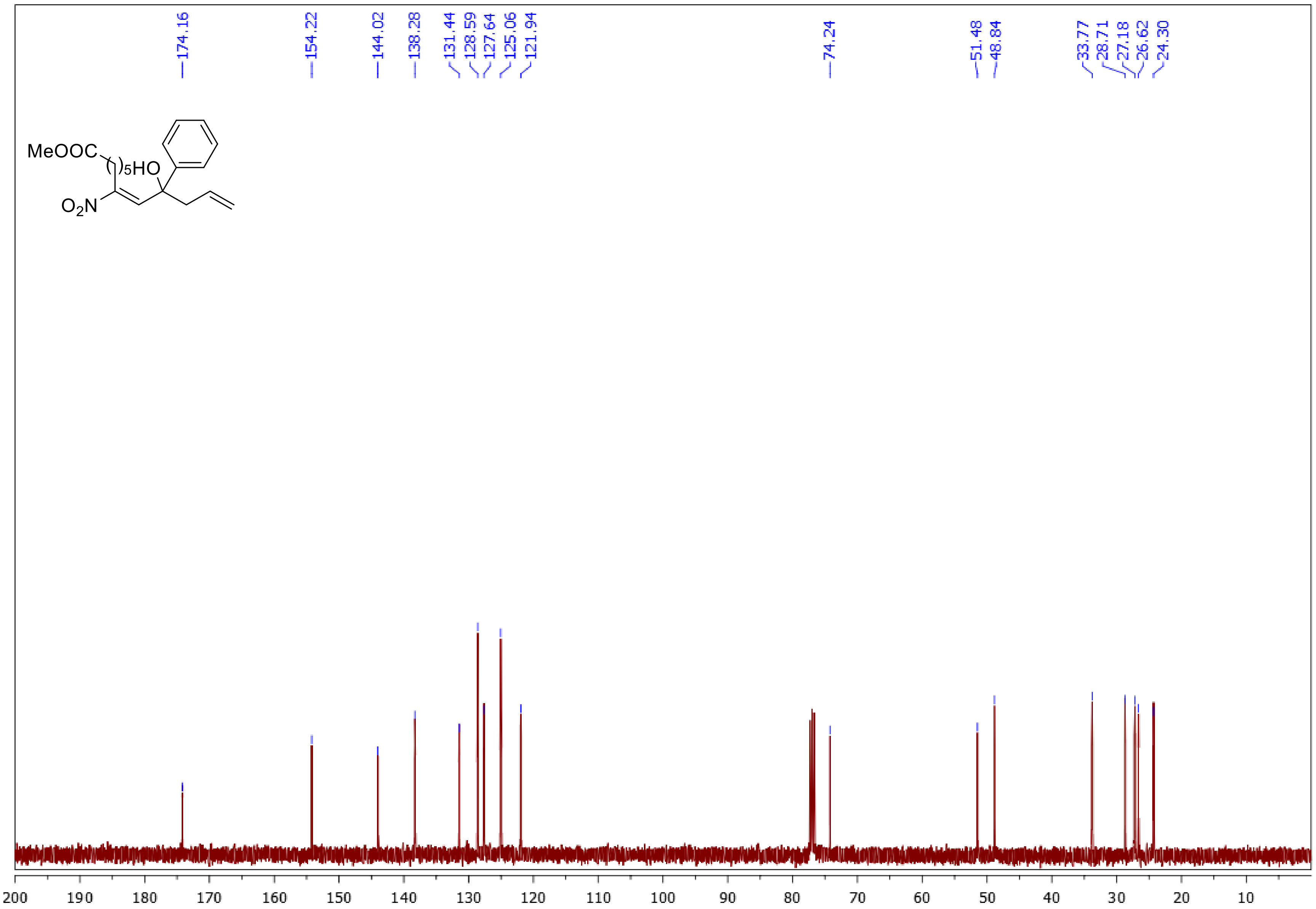

$^{1}$H NMR (400 MHz, $CDCl_3$) Compound **3o**.

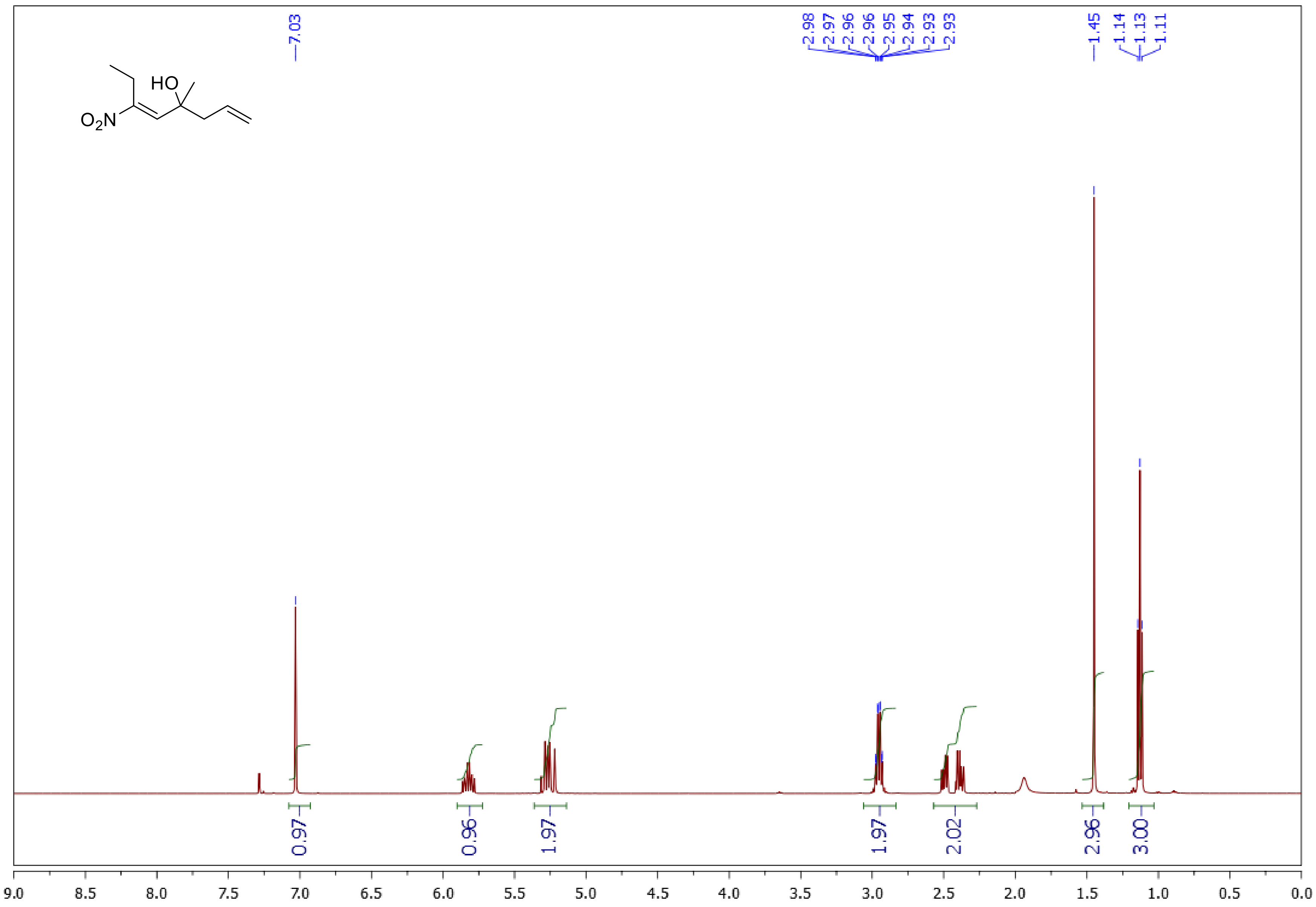

$^{13}C\{^{1}H\}$ NMR (100 MHz, $CDCl_3$) Compound **3o**.

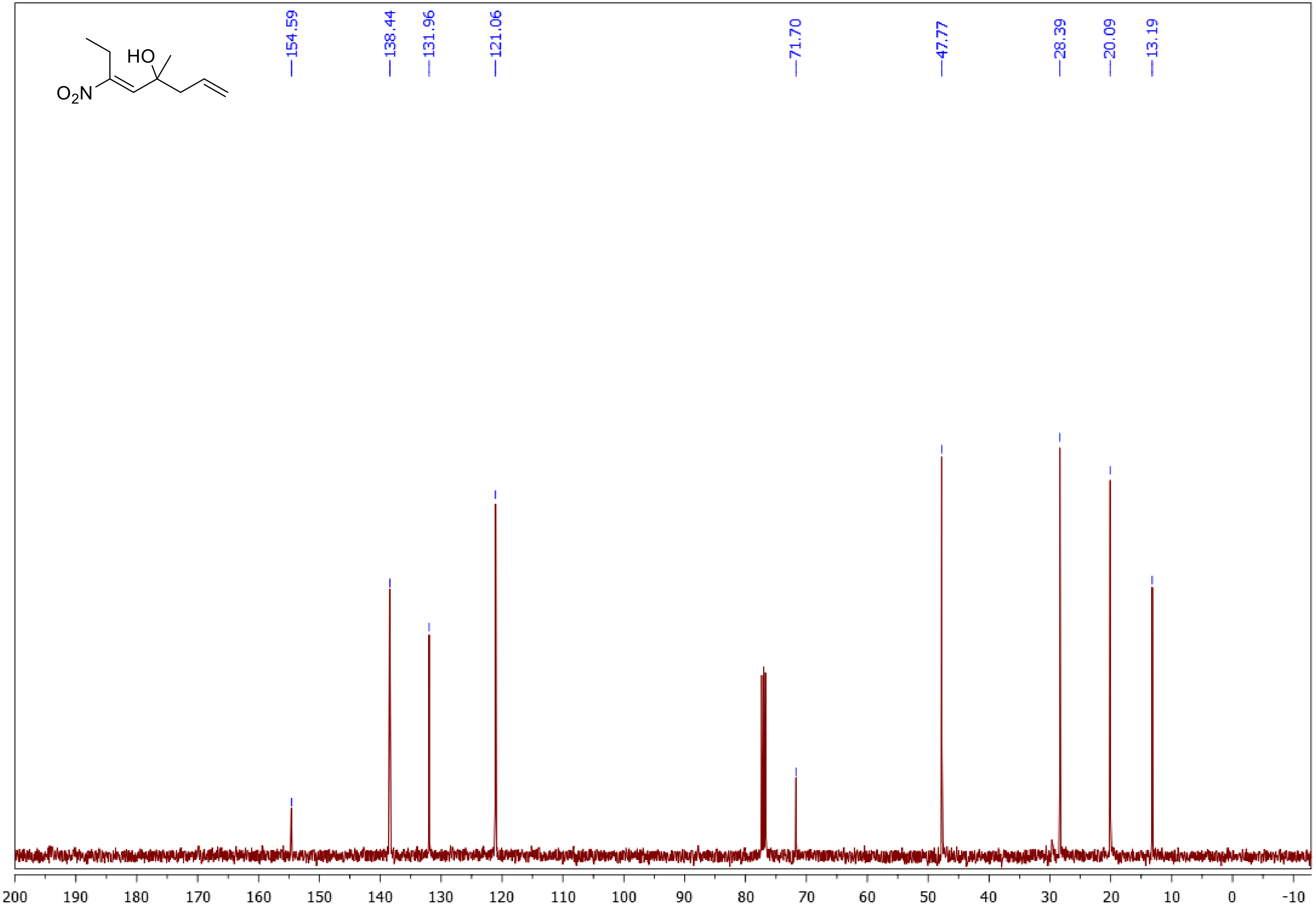

## 5. Copy of $^{1}H$ NMR and $^{13}C\{^{1}H\}$ NMR of compounds **4**.

$^{1}H$ NMR (400 MHz, $CDCl_3$) Compound **4a**.

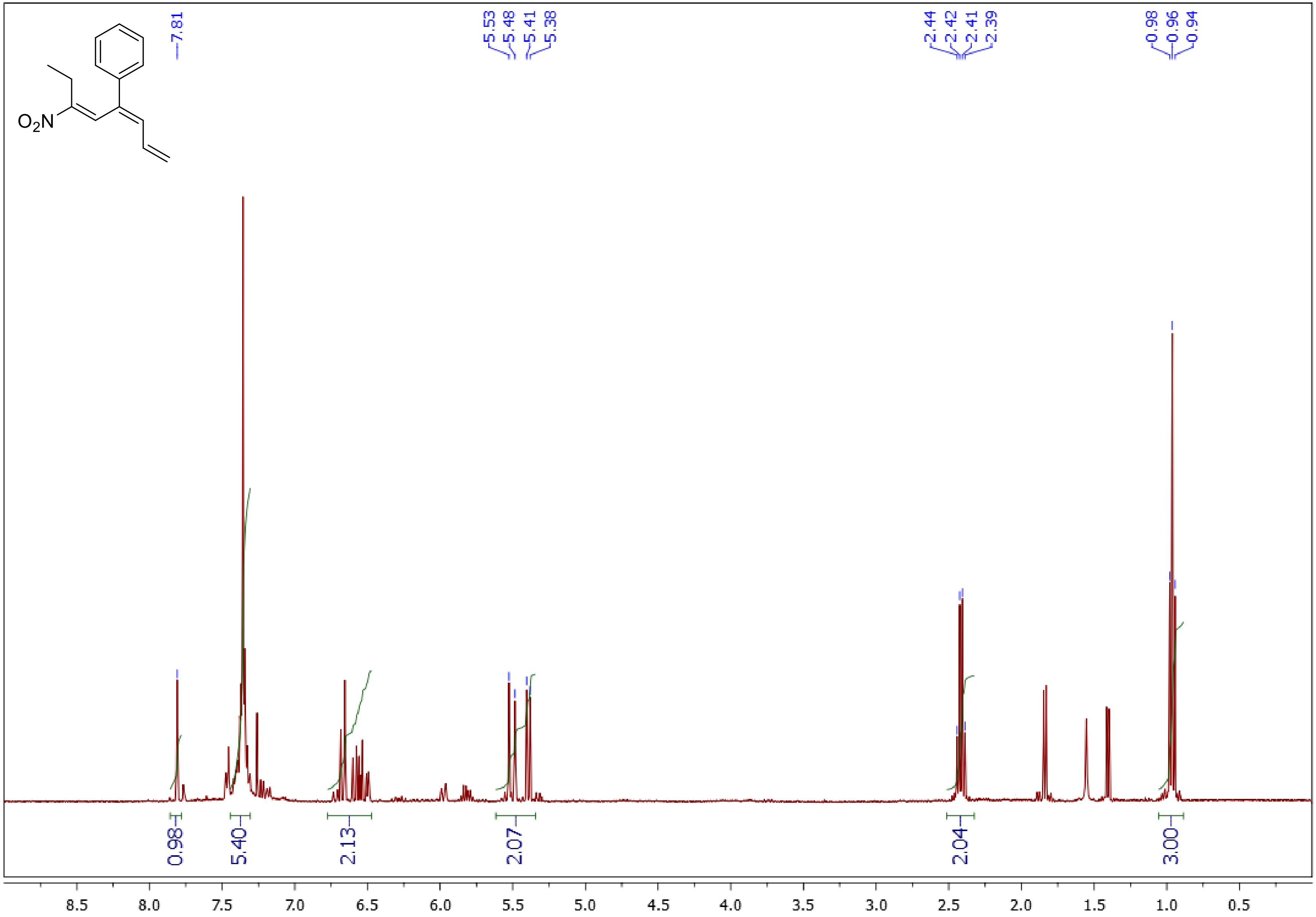

$^{13}C\{^1H\}$ NMR (100 MHz, $CDCl_3$) Compound **4a**.

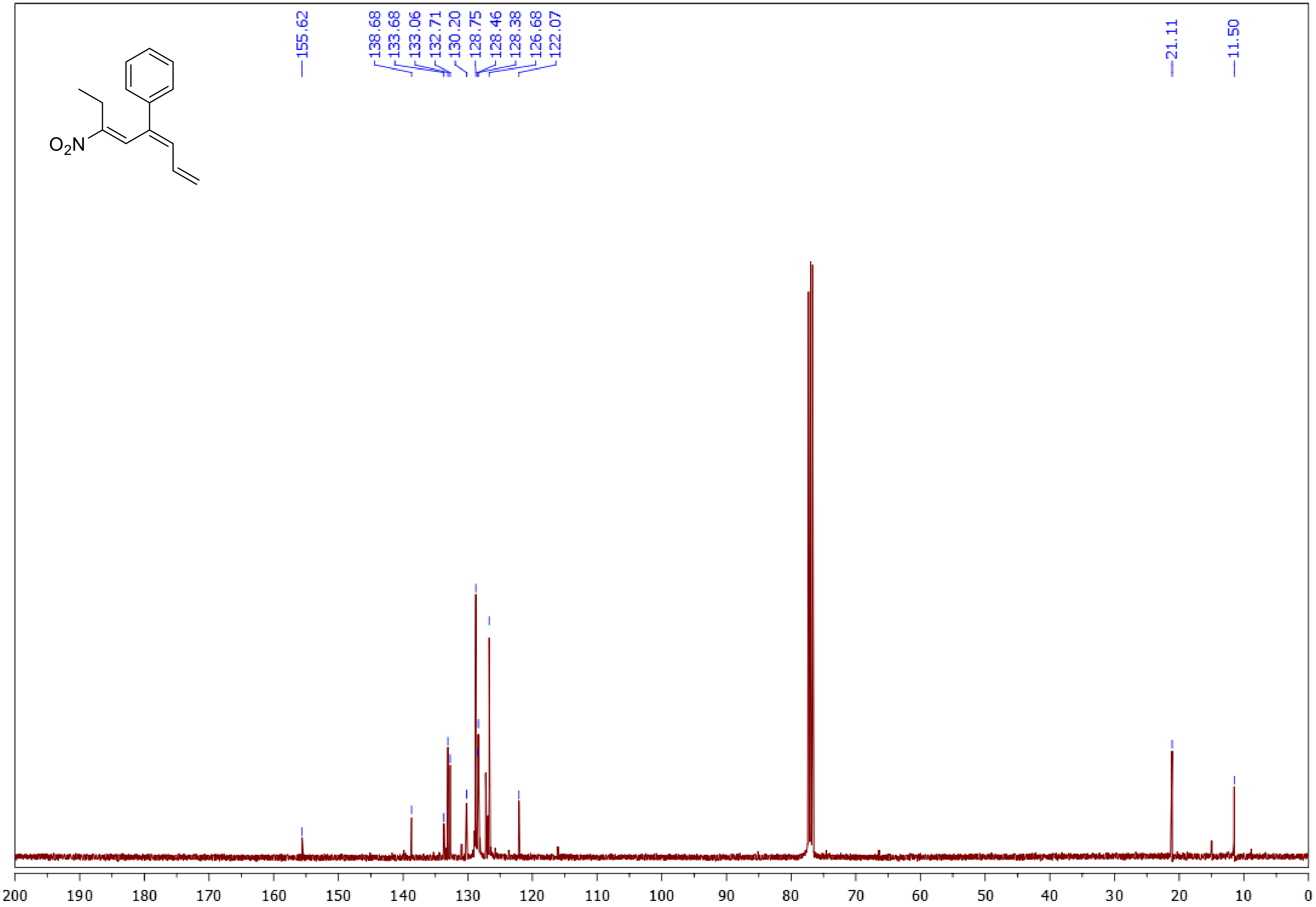

$^{1}$H NMR (400 MHz, $CDCl_3$) Compound **4b**.

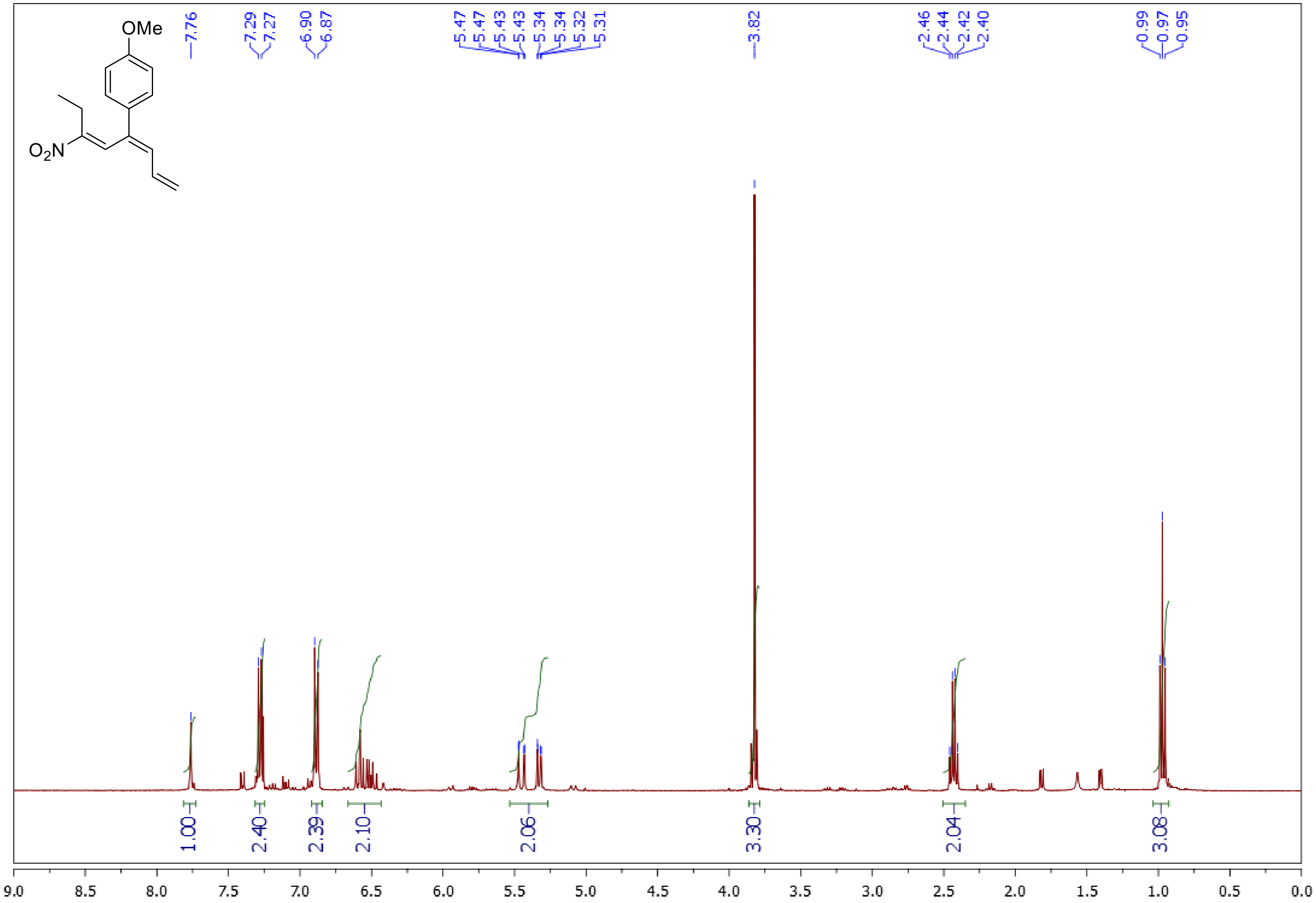

$^{13}C\{^{1}H\}$ NMR (100 MHz, $CDCl_3$) Compound **4b**.

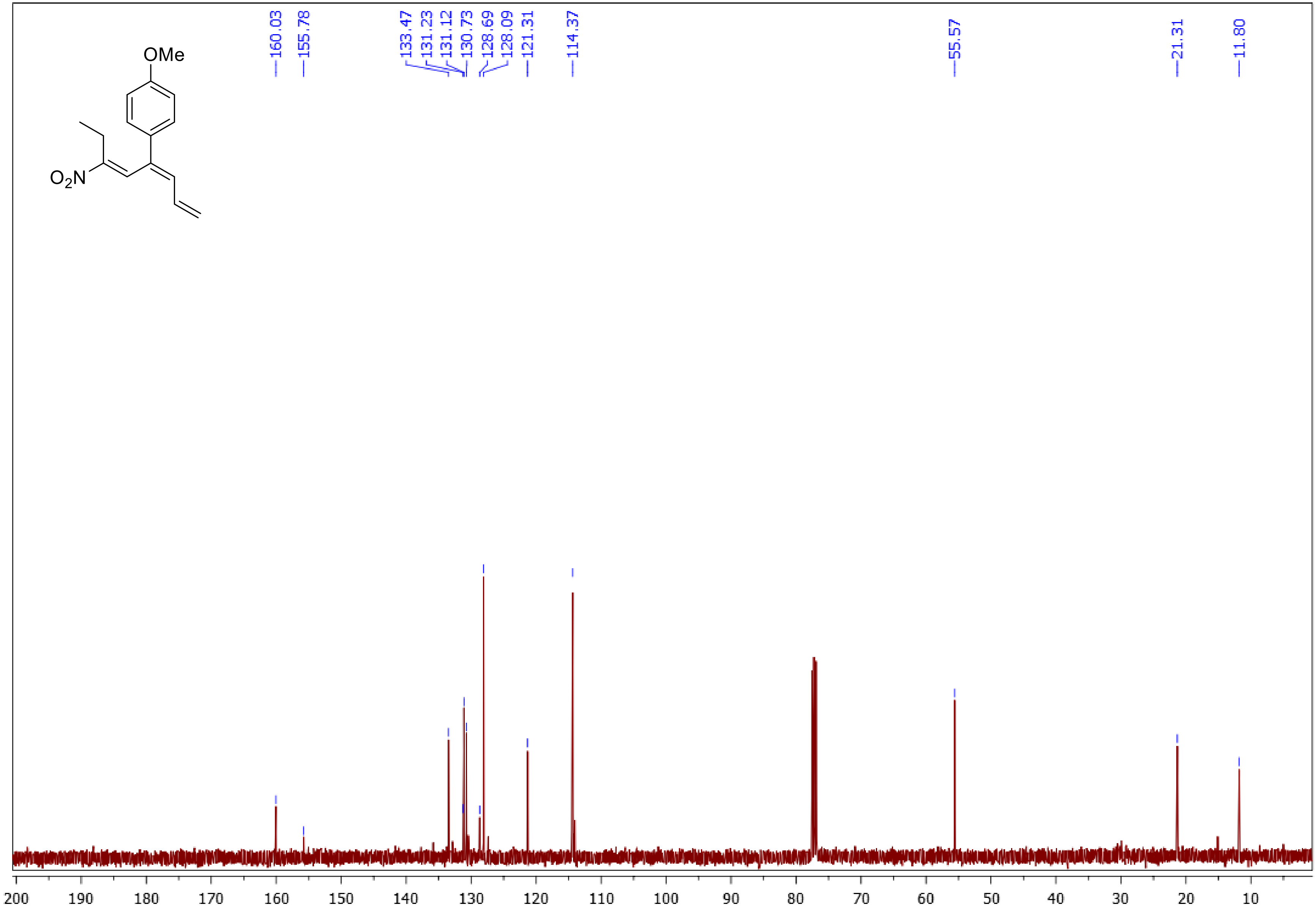

$^{1}$H NMR (400 MHz, $CDCl_3$) Compound **4c**.

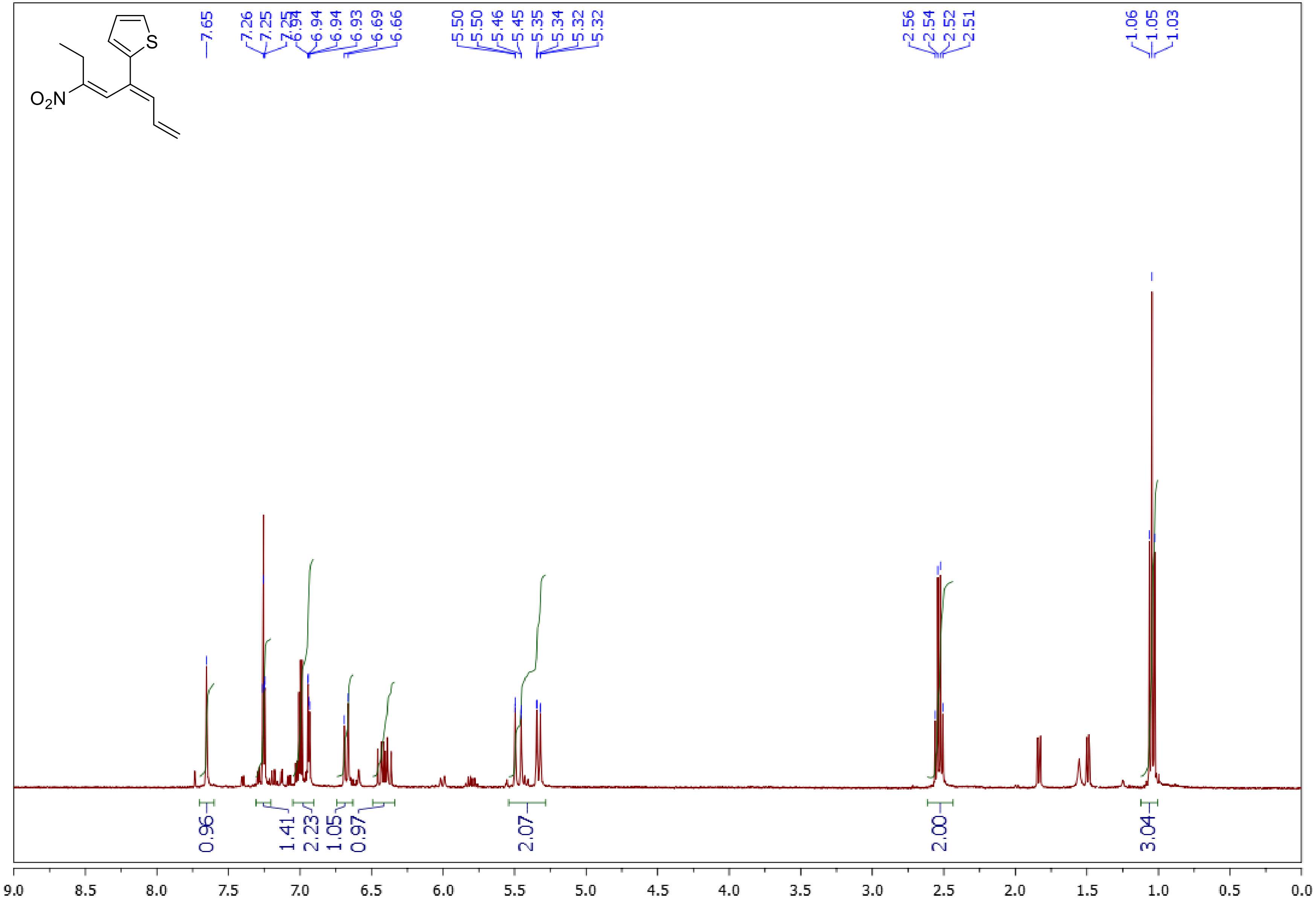

$^{13}C\{^{1}H\}$ NMR (100 MHz, $CDCl_3$) Compound **4c**.

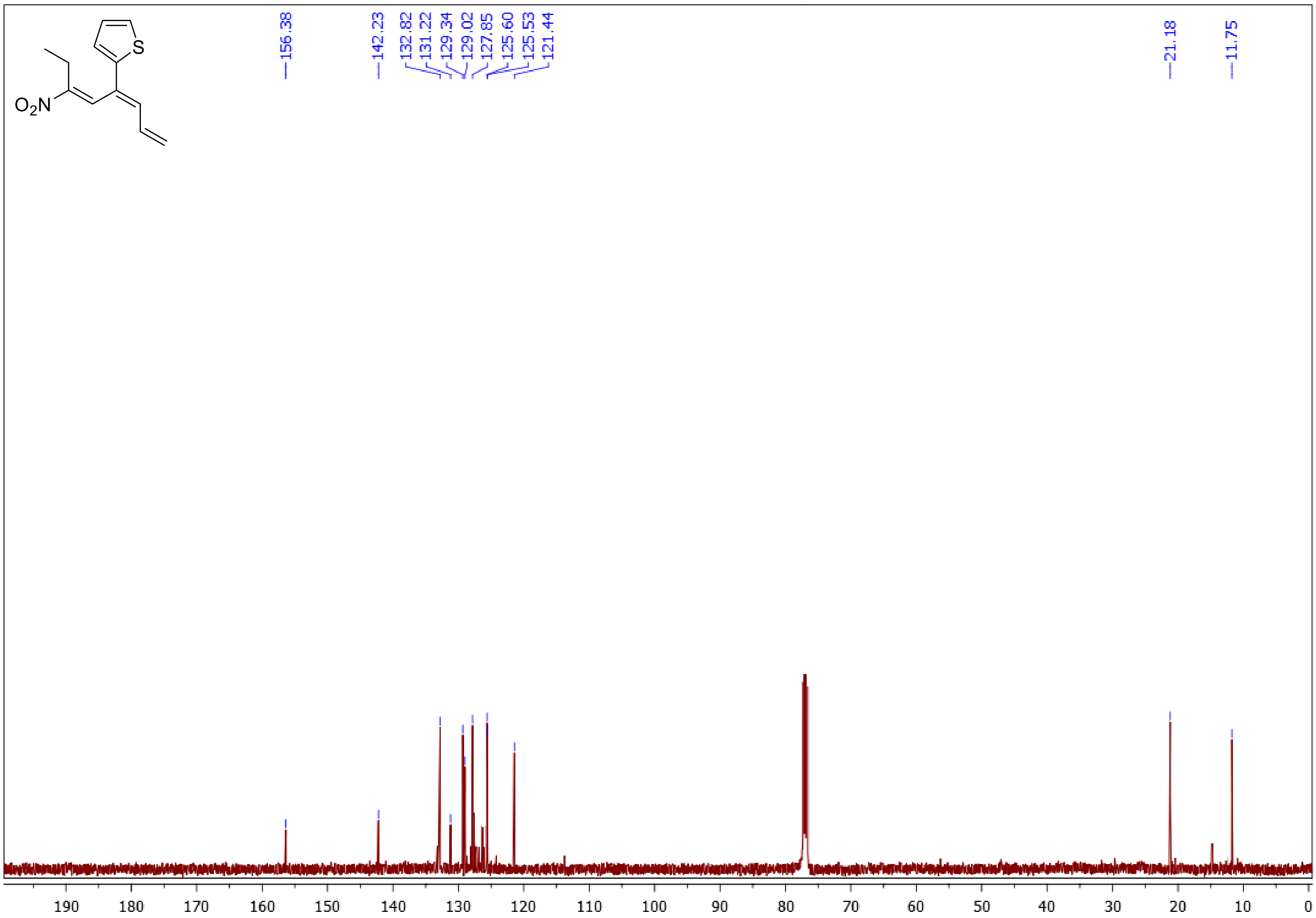

$^{1}$H NMR (400 MHz, $CDCl_3$) Compound **4e**.

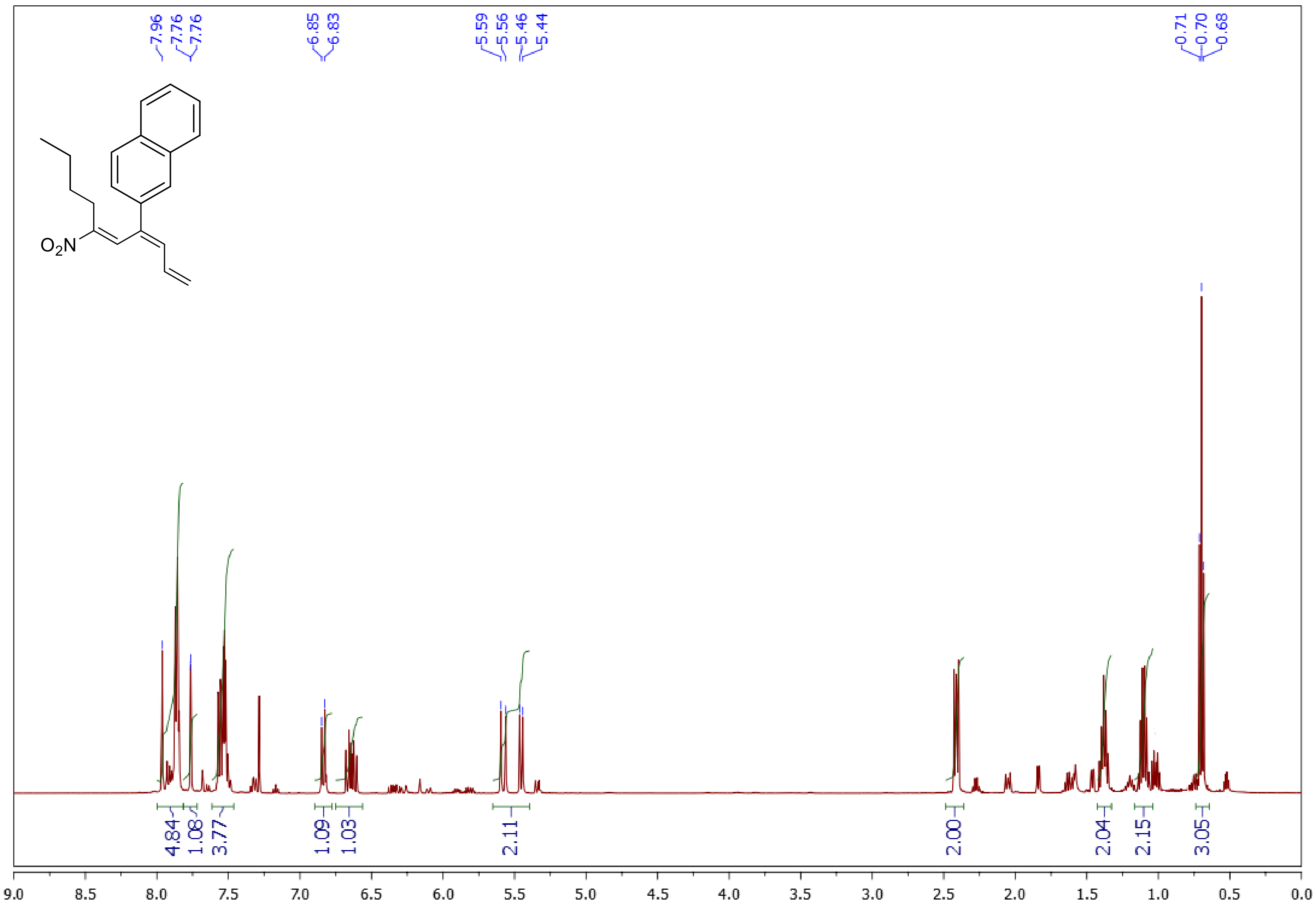

$^{13}C\{^1H\}$ NMR (100 MHz, $CDCl_3$) Compound **4e**.

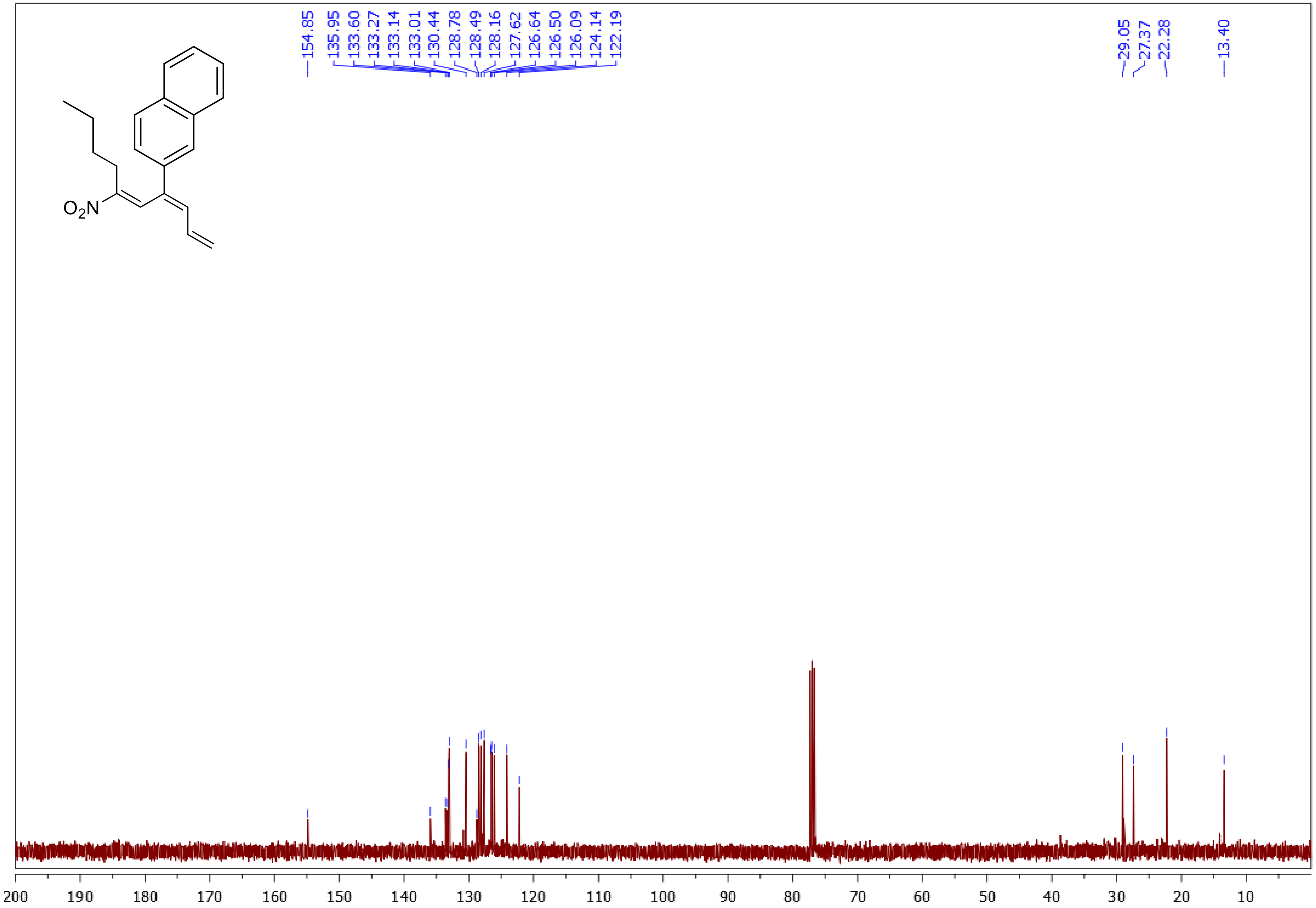

$^{1}H$ NMR (400 MHz, $CDCl_3$) Compound **4i**.

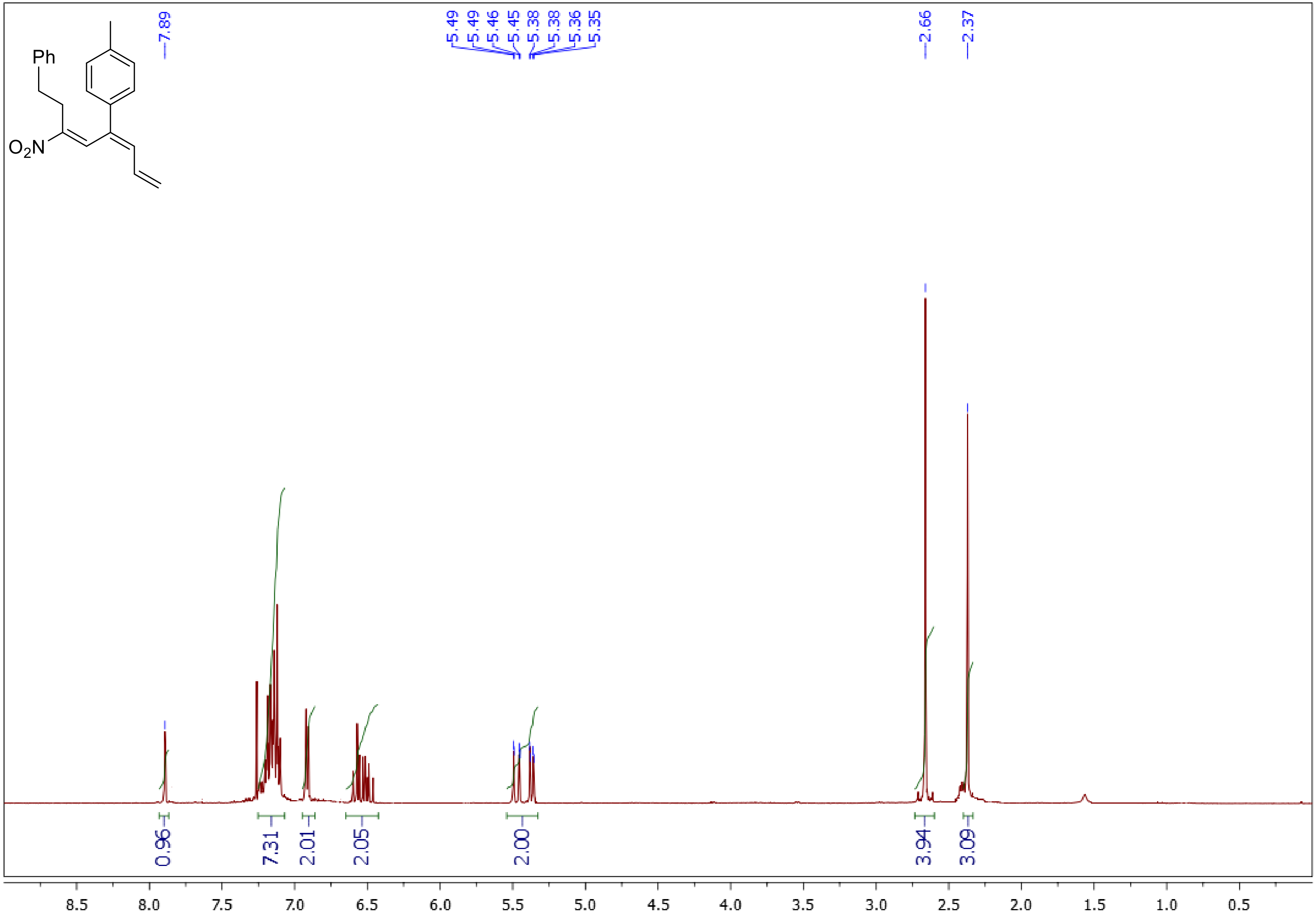

$^{13}C\{^1H\}$ NMR (100 MHz, $CDCl_3$) Compound **4i**.

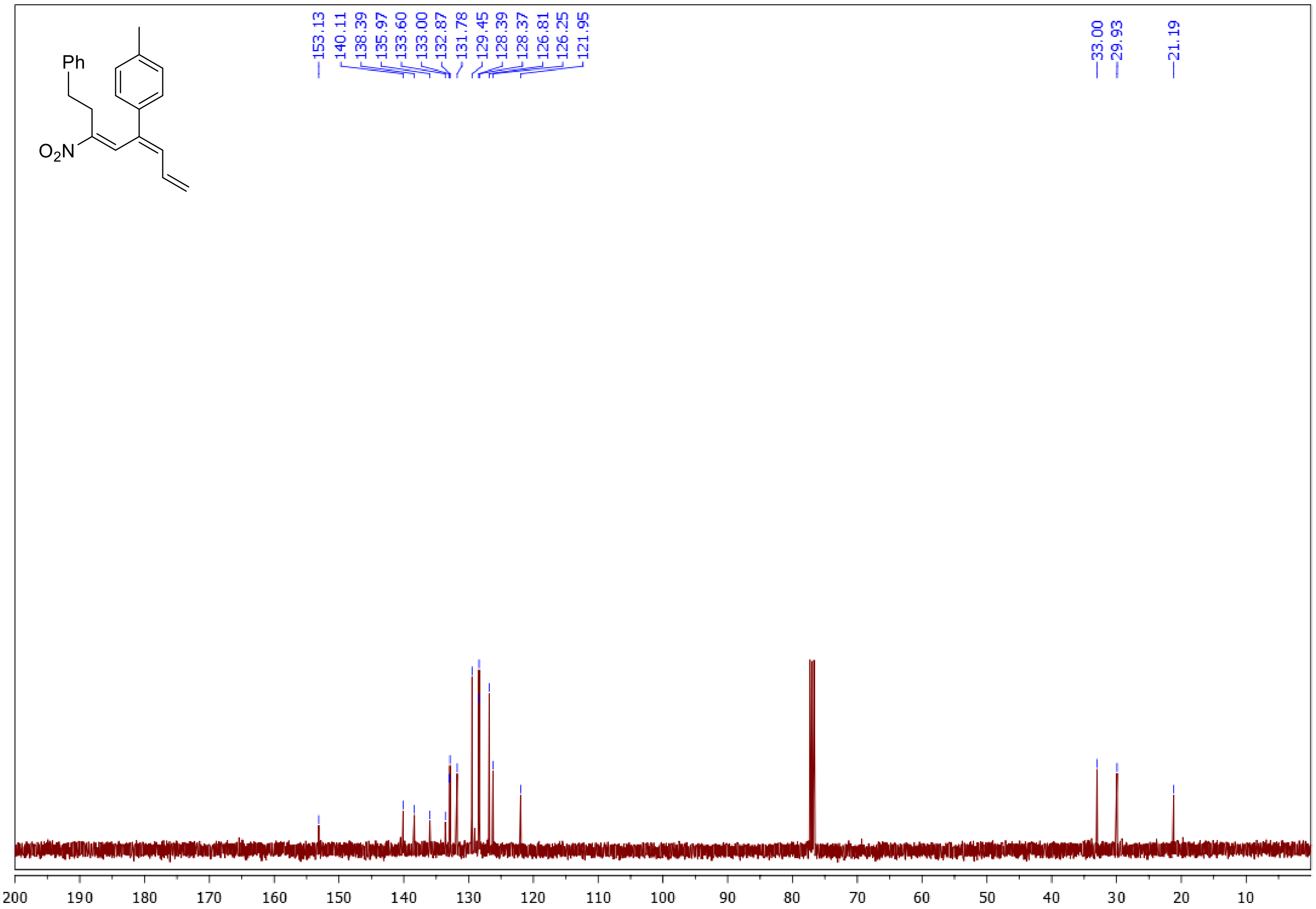

$^{1}H$ NMR (400 MHz, $CDCl_3$) Compound **4l**.

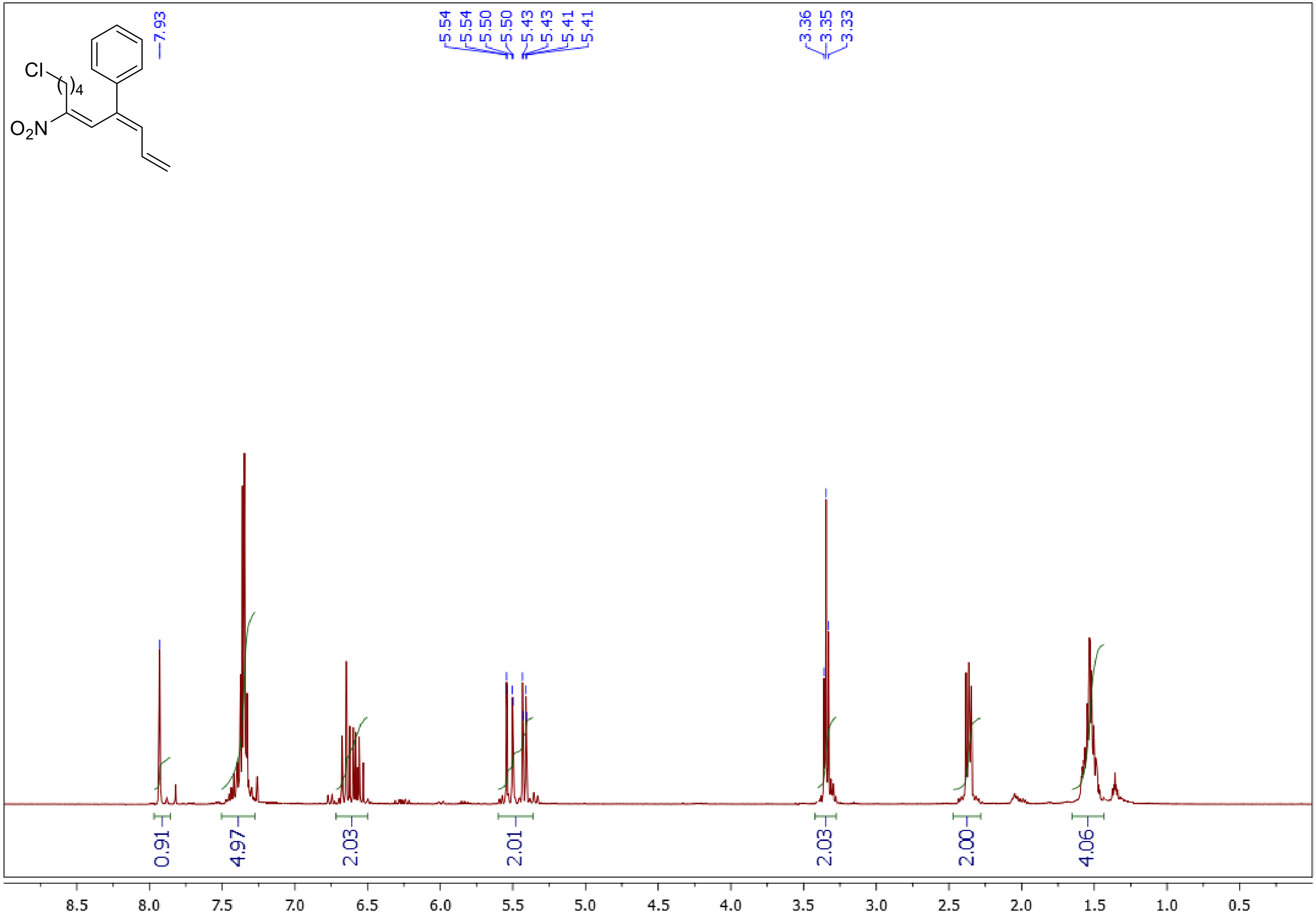

$^{13}C\{^{1}H\}$ NMR (100 MHz, $CDCl_3$) Compound **4l**.

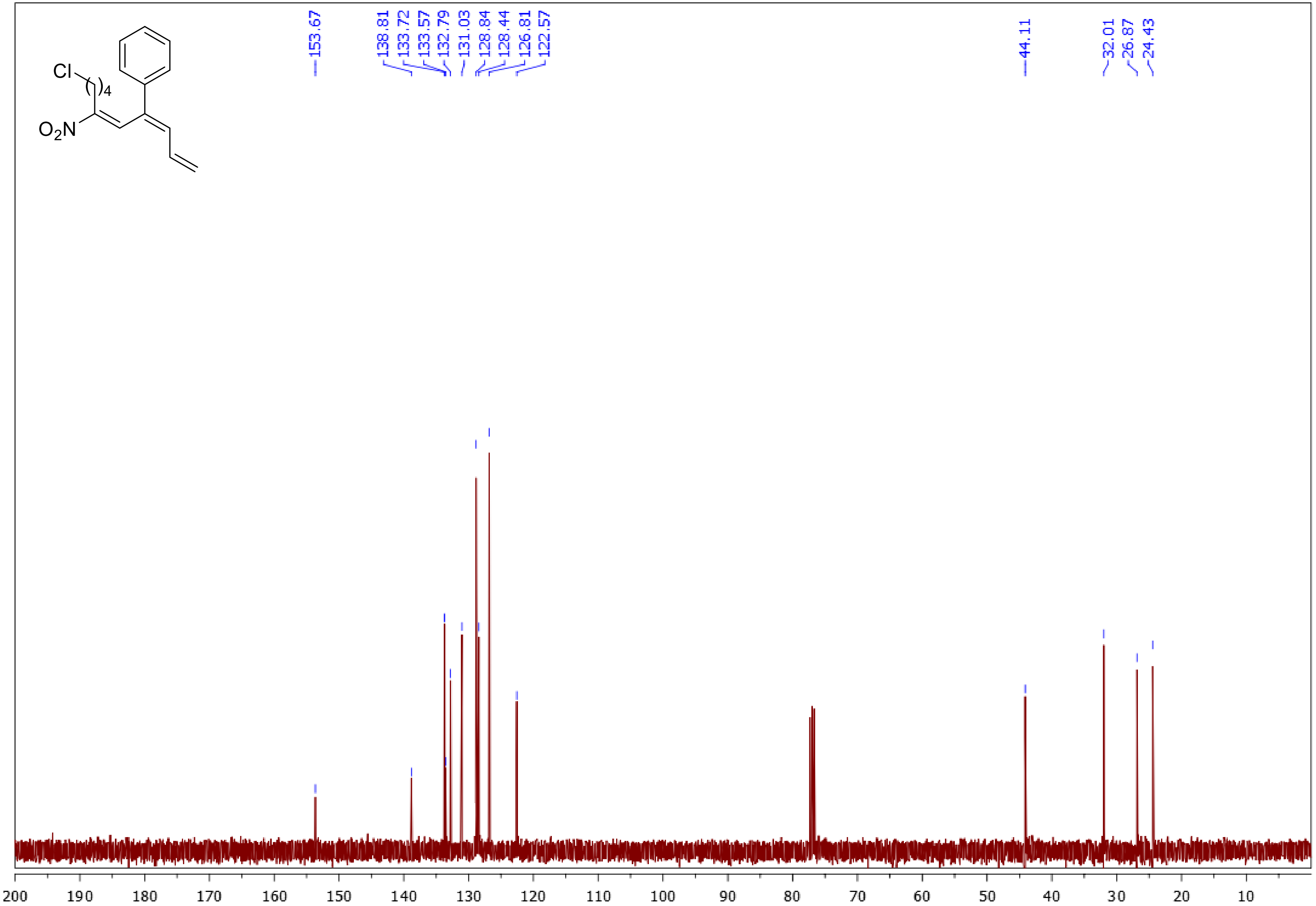

$^{1}$H NMR (400 MHz, $CDCl_3$) Compound **4n**.

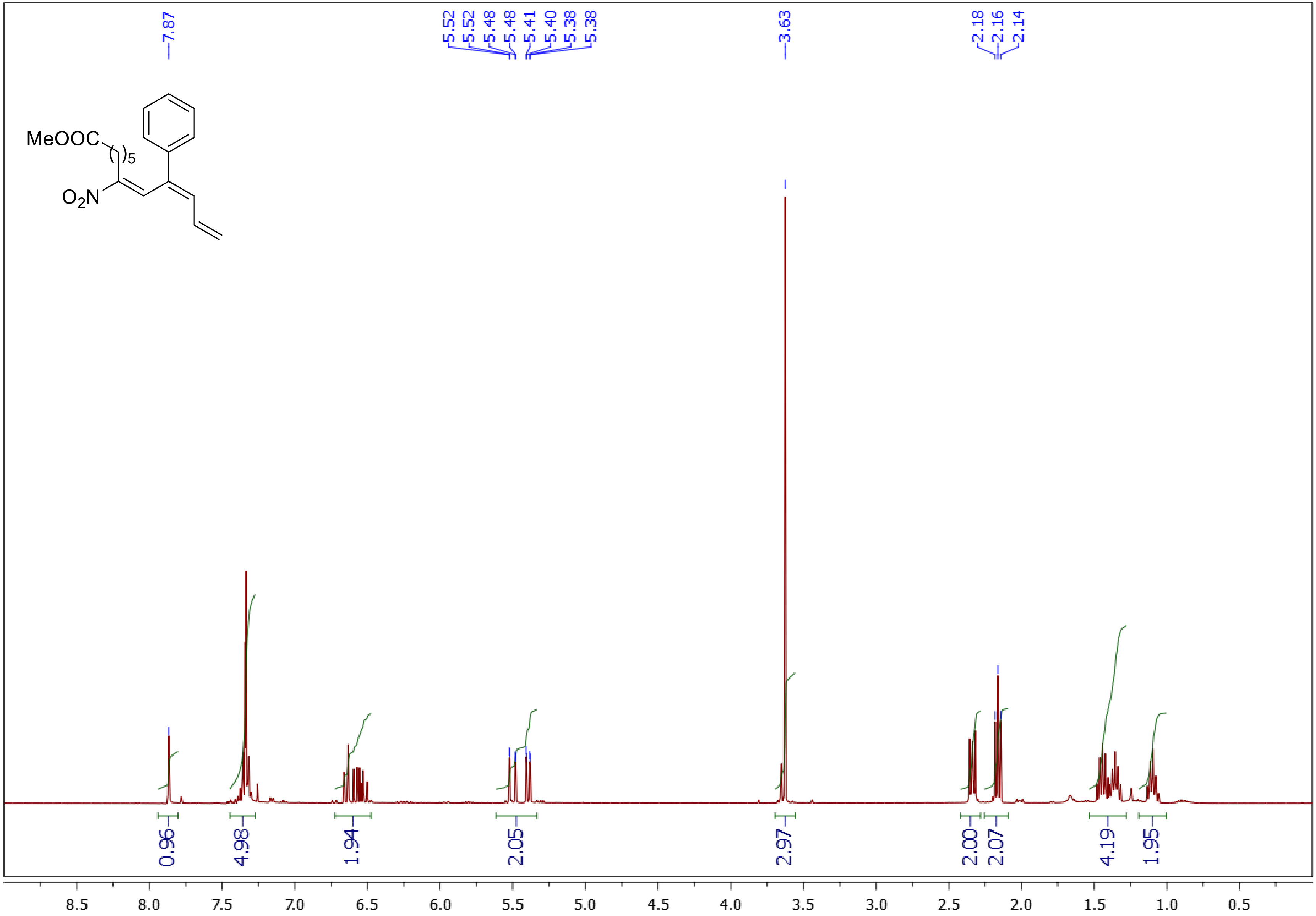

$^{13}C\{^1H\}$ NMR (100 MHz, $CDCl_3$) Compound **4n**.

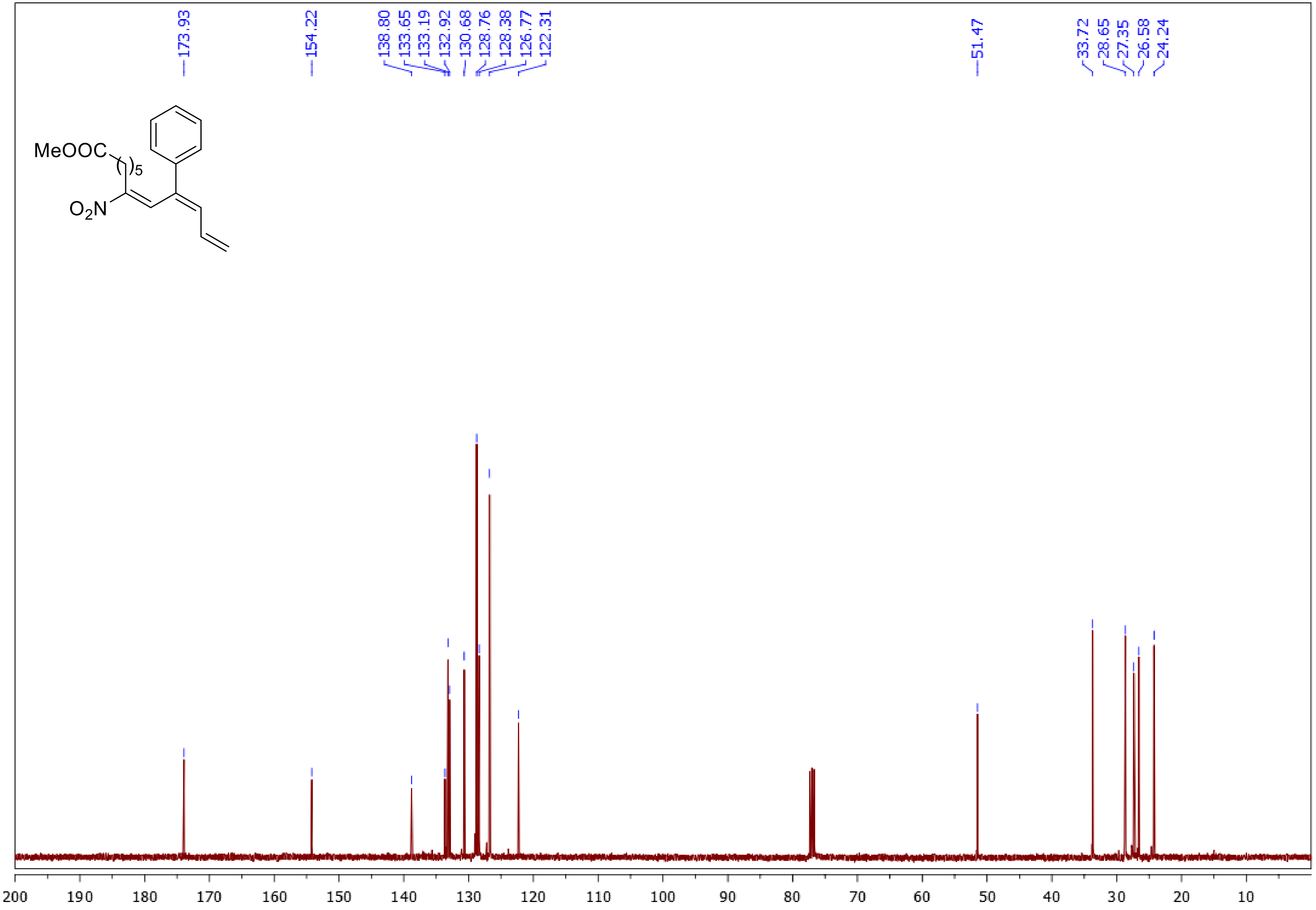

6. NOESY in $CDCl_3$ (400 MHz) of Compound **4a**.

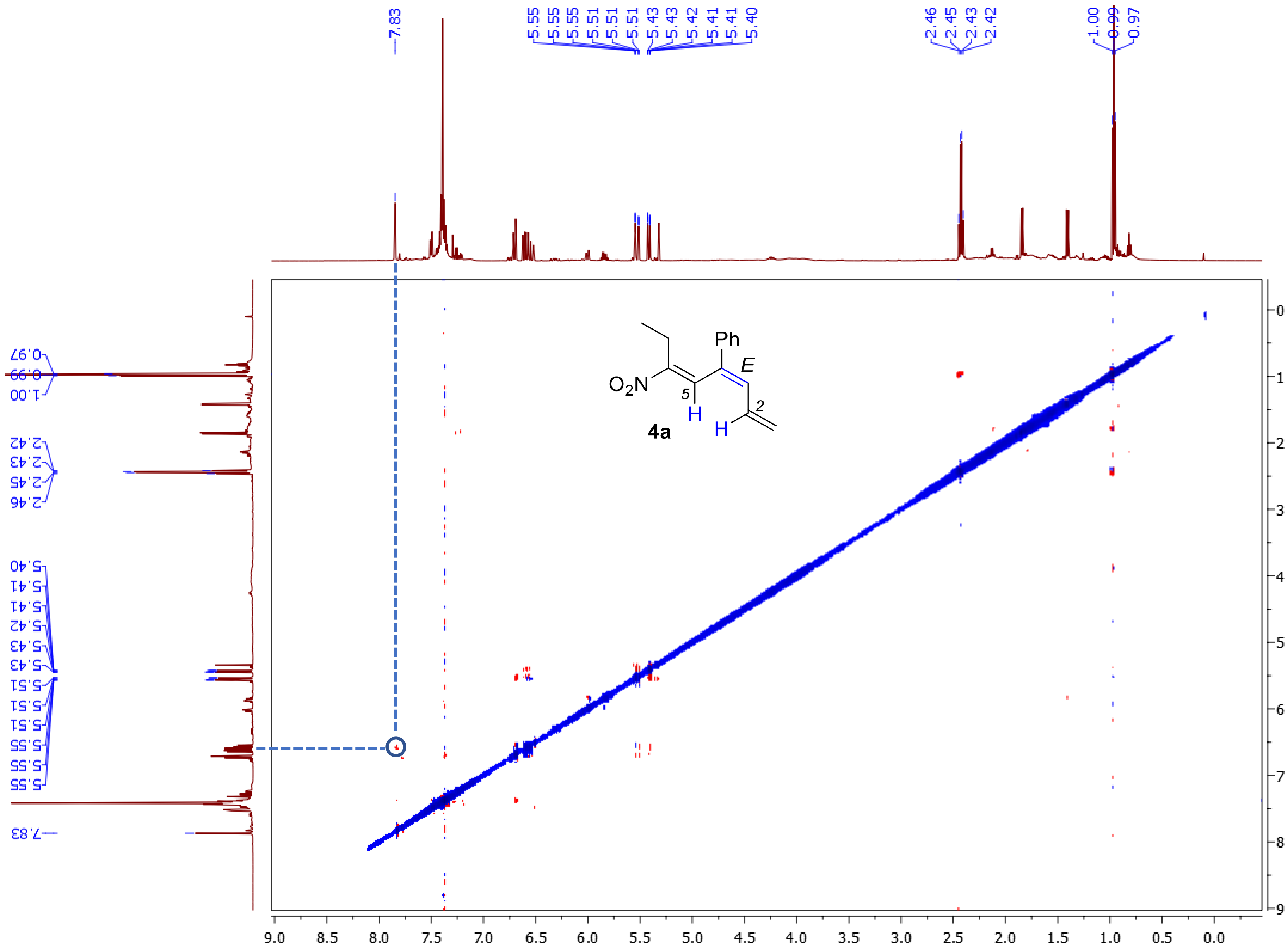

Supplement: Supplementary file 1 — jo2c02669_si_001.pdf [file jo2c02669_si_001.pdf]
